# Site‐Selective Tyrosine Reaction for Antibody‐Cell Conjugation and Targeted Immunotherapy

**DOI:** 10.1002/advs.202305012

**Published:** 2023-12-03

**Authors:** Hongfei Chen, Hong‐Chai Fabio Wong, Jiaming Qiu, Biquan Li, Dingdong Yuan, Hao Kong, Yishu Bao, Yu Zhang, Zhiyi Xu, Ying‐Lung Steve Tse, Jiang Xia

**Affiliations:** ^1^ Department of Chemistry The Chinese University of Hong Kong Shatin Hong Kong SAR China

**Keywords:** antibody reaction, antibody‐cell conjugation, antibody‐drug conjugates, cancer immunotherapy, photoaddition, tyrosine reaction

## Abstract

Targeted immunotherapies capitalize on the exceptional binding capabilities of antibodies to stimulate a host response that effectuates long‐lived tumor destruction. One example is the conjugation of immunoglobulins (IgGs) to immune effector cells, which equips the cells with the ability to recognize and accurately kill malignant cells through a process called antibody‐dependent cellular cytotoxicity (ADCC). In this study, a chemoenzymatic reaction is developed that specifically functionalizes a single tyrosine (Tyr, Y) residue, Y296, in the Fc domain of therapeutic IgGs. A one‐pot reaction that combines the tyrosinase‐catalyzed oxidation of tyrosine to *o*‐quinone with a subsequent [3+2] photoaddition with vinyl ether is employed. This reaction installs fluorescent molecules or bioorthogonal groups at Y296 of IgGs or the C‐terminal Y‐tag of an engineered nanobody. The Tyr‐specific reaction is utilized in constructing monofunctionalized antibody‐drug conjugates (ADCs) and antibody/nanobody‐conjugated effector cells, such as natural killer cells or macrophages. These results demonstrate the potential of site‐selective antibody reactions for enhancing targeted cancer immunotherapy.

## Graphical abstract

Achieving site‐selective reactions at a single tyrosine residue, while leaving others untouched within the same protein, can be challenging. Here, a one‐pot chemoenzymatic reaction is developed to install fluorescent or bioorthogonal groups at Y296 on the Fc domain of IgGs or a Y‐tag of an engineered nanobody, and applied it to construct monofunctionalized antibody‐drug conjugates and antibody/nanobody‐conjugated effector cells.

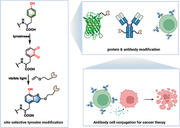

## 1 Introduction

Targeted immunotherapies rely on the specific recognition of cancer cells for the precise killing of tumors.^[^ ^1^, ^2^, ^3^, ^4^, ^5^ ^]^ For example, antibodies bring immune effector cells to the malignant cancer cells and eradicate the latter, an effect called antibody‐dependent cellular cytotoxicity (ADCC).^[^ ^6^, ^7^ ^]^ The antibody here serves as an engager to mediate cell‐cell contacts in ADCC: The Fab domain of an Immunoglobulin G (IgG) antibody binds to the antigen expressed on the surface of cancer cells, and the Fc domain binds to the Fc receptor on the surface of immune effector cells. Multiple methods have been developed to harness the engaging mechanism for cancer treatments. Clinicians directly inject monoclonal antibodies (mAbs) as engagers into patients to recruit immune effector cells to cancer cells.^[^ ^1^, ^2^, ^3^, ^4^, ^5^ ^]^ Alternatively, synthetic engagers, i.e., antibody‐derived designer molecules, including bispecific antibodies, biTE, *etc*., have also been devised.^[^ ^8^, ^9^, ^10^ ^]^ The synthesis of these new‐generation engagers often utilizes antibody reactions or protein engineering techniques to modify antibodies or antibody‐like molecules such scFv, nanobody, Fab, etc.^[^ ^8^, ^9^, ^10^ ^]^ The third method is to directly embed, display, or conjugate the engagers on the surface of immune effector cells and use the cells for therapeutic treatments.^[^ ^11^, ^12^ ^]^ For example, transfecting patient‐derived T cells with plasmids expressing CAR results in CAR‐T therapy; success of CAR‐T has been widely achieved in blood cancers, although solid tumors remain difficult to be treated.^[^ ^12^ ^]^

Instead of engineering immune cells through plasmid transfection, chemists traveled another path: chemically conjugating antibodies on the surface of immune effector cells is a convenient way to install engaging antibodies on effect cells. Antibody‐directed cellular cytotoxicity toward target cancer cells has been successfully observed using antibody‐conjugated effector cells. For example, Francis and coworkers developed a tyrosinase‐mediated method to conjugate tyrosine‐tagged nanobodies with nucleophilic residues on the surface of NK cells, and synthesized nanobody‐cell conjugates.^[^ ^13^ ^]^ Antibody‐functionalized capsules or exosomes realized effective targeting of tumor cells and induction of antitumor T‐cell responses.^[^ ^14^, ^15^ ^]^ Meyer and coworkers conjugated 5′ NHS ester ssDNA linker 1 with the cell surface proteins of effector cells and 5′ NHS ester ssDNA linker 2 with an antibody. Hybridization of ssDNA linker 1 and ssDNA linker 2 results in antibody‐conjugated cells.^[^ ^16^ ^]^ Li and coworkers utilized a similar strategy to trastuzumab to oNK cell line.^[^ ^17^ ^]^ Wei and coworkers designed a chimeric antibody‐nucleic acid T‐cell engager system with sophisticated programmable DNA nanoassemblies to bridge cancer cells and T cells.^[^ ^18^ ^]^ Wu and coworkers used a fucosyltransferase to transfer a GDP‐modified IgG antibody to the glycocalyx on the surfaces of live cells. This method modifies the carbohydrates of IgG and glycocalyx on the cell surface.^[^ ^19^ ^]^ However, in the reactions used for conjugating antibodies to cells, most researchers used the NHS‐amine reaction to chemically functionalize IgGs, which resulted in random orientations of IgGs on cell surface. Some of the orientations may shield the antigen binding sites. In this regard, site‐selective antibody reactions at the Fc region will provide precise control of the orientation.

In the pursuit of site‐selective antibody reactions, bioorthogonal reactions provide us a rich toolkit.^[^ ^20^, ^21^ ^]^ Protein reactions are most conveniently done at lysine residues with commercially available *N*‐hydroxy succinimide (NHS)‐activated esters, sulfonyl chlorides, or iso(thio)cyanates, or at cysteine residues with disulfide bond exchangers or maleimides.^[^ ^22^, ^23^ ^]^ Reactions at other amino acids have also been reported, but with less popular use in real applications.^[^ ^24^, ^25^, ^26^, ^27^, ^28^ ^]^ Among these natural amino acids, the phenol group on the side chain of tyrosine (Tyr, Y) can undergo a variety of reactions such as Mannich‐type reaction, diazonium reaction, sulfur fluoride exchange chemistry (SuFEx), triazolinedione reaction, transition metal‐catalyzed reaction, etc.^[^ ^29^, ^30^, ^31^, ^32^, ^33^, ^34^, ^35^, ^36^, ^37^, ^38^, ^39^, ^40^, ^41^, ^42^ ^]^ The most recent tyrosine reactions include electrochemical reactions with urazoles^[^ ^43^ ^]^ or phenothiazine derivatives^[^ ^44^ ^]^ and photocatalytic reactions catalyzed by tris(2,2′‐bipyridyl) complex ([Ru(bpy)_3_]^2+[^ ^45^ ^]^ and with dinitroimidazole reagents,^[^ ^46^ ^]^ and photoreactions.^[^ ^47^, ^48^, ^49^ ^]^ For example, Macmillan and coworkers used lumiflavin, a water‐soluble photocatalyst, to drive the reaction of tyrosine with formyl groups, for the synthesis of structurally defined fluorescent conjugates from native proteins.^[^ ^50^ ^]^ These examples manifested the versatility of tyrosinyl phenol in protein reactions.

**Figure 1:**
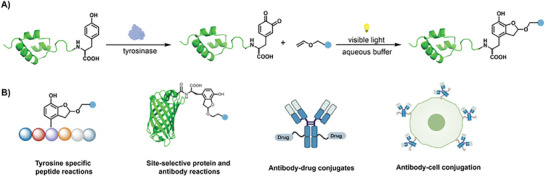
Site‐selective Tyr reaction by light‐induced *o*‐quinone photoaddition. A) Reaction scheme of Tyr‐specific protein modification via tyrosinase oxidation and *o*‐quinone visible‐light‐induced photoaddition in aqueous solutions. B) Applications of the Tyr reaction including functionalization of peptides, recombinant proteins, therapeutic antibodies, construction of antibody‐drug conjugates, and antibody‐cell conjugation, etc.

Enzymatic catalysis can convert the side chain phenol to new functional groups, introducing new Tyr modification strategies. Tyrosinases (EC 1.14.18.1) are rate‐limiting enzymes in melanin biosynthesis that catalyze the *o*‐hydroxylation of monophenols to catechols and quinones. Although the natural substrate of tyrosinases – for example, the commercially available tyrosinase from the mushroom *Agaricus bisporus* – is small molecule phenols, Tyr residues at certain positions of peptides and proteins can also be the substrate. Tyrosinase‐catalyzed oxidation thereby introduces reactive *o*‐quinone in proteins under mild conditions. Subsequently, *o*‐quinone can undergo a bioconjugation reaction with cysteines of another protein^[^ ^51^ ^]^ or a strain‐promoted [4 + 2] cycloaddition (SPOCQ) reaction with various strained cyclic alkynes such as bicyclo[6.1.0]nonyne (BCN) derivatives.^[^ ^52^, ^53^, ^54^, ^55^ ^]^ For example, Tirelli and coworkers restricted the oxidation reaction to catechols and “clicked” catechols with boronic acid‐containing hyaluronic acid to derive protein‐carbohydrate conjugates.^[^ ^56^ ^]^ Besides the commonly used mushroom tyrosinase, Francis and coworkers introduced a new bacterial tyrosinase for the derivatization of *o*‐quinone with amine nucleophiles under mild conditions.^[^ ^57^ ^]^ Micklefiled and coworkers devised a tandem enzymatic reaction of a fungal tyrosinase and a mammalian catechol‐O‐methyltransferase (COMT) which gave *O*‐alkylated products.^[^ ^58^ ^]^ On another note, *o*‐quinone was reported to react with vinyl ethers (VEs) in organic solvents under photoactivation conditions but not yet with proteins in aqueous solutions. In 1989, Akio and Miho reported a photoaddition reaction between *o*‐quinone and vinyl ethers (VEs) under light irradiation (> 340 nm) in organic solvents (benzene or acetonitrile) (**Figure** **1**A).^[^ ^59^ ^]^ We envision that the [3+2] photoaddition reaction may also occur in the aqueous solution under visible lights, allowing electron‐rich VEs to add to *o*‐quinone‐containing proteins derived from tyrosinase oxidation (Figure 1B). Regarding the protein substrates, most tyrosinase‐catalyzed reactions took place at the terminal tyrosine on recombinant proteins; tyrosine‐containing protein tags such as the N‐terminal MYGG‐, and the C‐terminal ‐RRRRY, or ‐EEEEY, or ‐GGGGY, collectively known as Y‐tags, have been engineered for residue‐specific Tyr labeling.^[^ ^57^, ^60^, ^61^ ^]^ For example, van Delft and coworkers genetically engineered a tetra‐glycyltyrosine tag to the antibody Trastuzumab (Tras) on both light chains; the resultant Tras[LC]G_4_Y can be selectively oxidized by tyrosinase and derivatized.^[^ ^55^, ^62^, ^63^ ^]^ The same group recently reported a non‐genetic‐engineering approach to the chemoenzymatic tyrosine click reaction: Removal of the N297 glycan of Tras by PNGase F significantly increased the mobility of Y296 and Y300 residues,^[^ ^64^, ^65^ ^]^ which will make the antibody a substrate for mushroom tyrosinase. Taken together, here, we develop a chemoenzymatic [3+2] photoaddition reaction that selectively functionalizes a unique tyrosine of the Fc domain and showcases the use of this reaction in ADC construction and antibody‐cell conjugation for targeted cancer immunotherapy.

## 2 Results and Discussion

### 2.1 [3+2] Photoaddition of Vinyl Ether To O‐Quinone in Aqueous Solution

**Figure 2:**
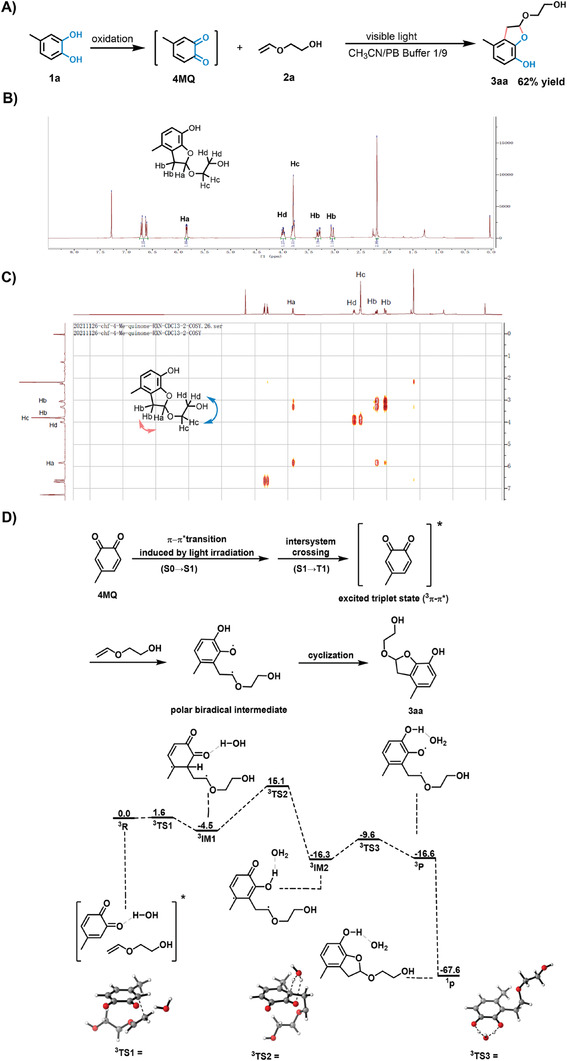
A model reaction of **4MQ** photoaddition in the aqueous solution. A) Oxidation of catechol **1a** followed by photoaddition of vinyl ether **2a** in an aqueous solution yields **3aa**. Reaction condition: **1a** (0.1 mmol), NaIO_4_ (0.1 mmol), and **2a** (0.5 mmol) were dissolved in MeCN/H_2_O (1 mL, 1:9). The reaction system was irradiated by blue light (456 nm, 30 mW cm^−2^) for 5 min at room temperature. B,C) ^1^H‐NMR and ^1^H‐^1^H COSY spectra of product **3aa** show the presence of a 5‐membered ring. Correlations between H_a_ and H_b_, H_c_ and H_d_ were indicated by arrows. D) Proposed mechanism of model reaction in aqueous buffer and the Free energy profile of [3+2]‐photoaddition of **4MQ** and **2a** in SMD water model.[cite SMD] Singlet and triplet states are labeled as superscripts “1” and “3”, respectively.

Diketone was known to undergo a photoaddition reaction with vinyl groups.^[^ ^66^ ^]^ Tyrosinase oxidizes the phenol group to a diketone. These two reactions inspired us to develop a Tyr‐specific reaction by conjugating the tyrosinase‐catalyzed *o*‐quinone with vinyl ether (VE). However, whether the photoaddition reaction can occur in the aqueous solution that proteins require is not reported. First, we set up a model reaction to explore the photoaddition reaction of VE to the diketone part of the *o*‐quinone in the aqueous solution. Oxidation of 4‐methylbenzene 1,2‐diol **1a** gave 4‐methyl‐*o*‐quinone (**4MQ**), which subsequently reacted with **2a** in a phosphate buffer (0.2 M, pH 6.5) with 10% acetonitrile under the irradiation of 456 nm light for 5 min (30 mW cm^−2^) (**Figure** **2**A). The reaction gave a five‐membered ring adduct **3aa** based on ^1^H‐NMR and ^1^H‐^1^H COSY NMR spectra analysis (Figure 2B,C; Figure S1 in the Supporting Information).

We propose that the reaction proceeds through a ^3^π‐π* process in the polar solvent (water): **4MQ** at the excited state conjugates with the vinyl ether to form a biradical intermediate, followed by an intramolecular cyclization step, and then the generation of a cycloaddition product. We have revealed more details of the mechanism by DFT calculations as summarized in Figure 2D (see also^[^ ^66^, ^67^ ^]^ for related mechanisms with two‐step excitations). 456 nm light source has been shown both experimentally and theoretically to be sufficient to excite ground state *o*‐benzoquinone (S0) to the first excited singlet state (S1).^[^ ^68^, ^69^ ^]^ Through intersystem crossing, S1 may then be converted into the first triplet state (T1), which is believed to be a ^3^π‐π* state for *o*‐quinones in polar solvents.^[^ ^70^ ^]^ Hence, we propose that upon radiation, **4MQ** will reach the ^3^π‐π* state to form a triplet exciplex with vinyl ether **2a**. Density Functional Theory (DFT) calculations (see the details in Supporting Information) were carried out to study the reaction mechanism and free energy barriers involved in the [3+2] photoaddition observed in the current study. Based on our computational results, the incident radiation allows a rapid radical addition between the reactants. After the exciplex formation, there exists a small barrier (1.6 kcal mol^−1^) between the quinone and the olefin to yield a triplet biradical intermediate **^3^IM1**. For the conversion from **^3^IM1** to **^3^P**, we propose that the reaction involves intramolecular proton transfers and is water‐assisted. The explicit water molecule present in our modeled system can act as a base to abstract the proton at C3 position of **^3^IM1**. Simultaneously, the hydronium‐like species in **^3^TS2** transfers a proton to the closest quinone oxygen to give **^3^IM2**. With a free energy barrier of 19.6 kcal mol^−1^, the first proton transfer is the rate determining step of the proposed mechanism. Subsequently, an interchange of a proton between the two quinone oxygens can proceed in a similar concerted manner with a free energy of 6.7 kcal mol^−1^. The cyclization of **^3^P** yields the ground state five‐membered ring product **3aa**, likely through non‐radiative transitions. One plausible pathway is that the resultant triplet biradical undergoes intersystem crossing to form an unstable singlet biradical, in which the two unpaired electrons with opposite spins couple to form a new sigma bond.^[^ ^71^ ^]^

### 2.2 Tyr Reactions of Peptides and Proteins

**Figure 3:**
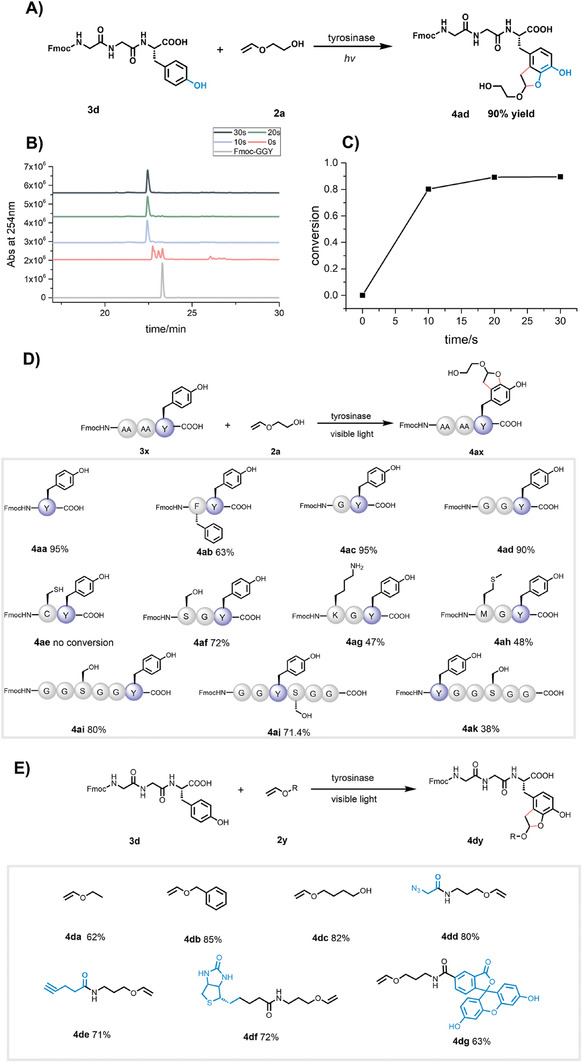
Tyrosine reaction in model peptides. A) Chemoenzymatic reaction of Fmoc‐GGY peptide. Reaction condition: Fmoc‐GGY‐OH (10 mM) was incubated with tyrosinase (1.68 µM) in the phosphate buffer (0.2 M, pH 6.5) at 4 °C for 30 min, and **2a** (100 mM) was added to the solution and irradiated by 456 nm blue light (30 mW cm^−2^, Kessil Lamp) for 5 min at room temperature. B) HPLC traces of the photoaddition reaction after enzyme oxidation. C) Kinetics of the normalized product formation. D) Substrate scope analysis using different Tyr‐containing peptides. E) The reaction of VE‐like molecules. Reaction conditions: peptide (10 mM) was incubated with tyrosinase (1.68 µM) in PB buffer (0.2 M, pH 6.5) at 4 °C for 30 min, then VE (100 mM) was added and irradiated at 456 nm light for 5 min. The reaction yield was detected by integrated area of the product in HPLC spectrum.

We next explored the Tyr reaction in short peptides that contain a C‐terminal Tyr in a phosphate buffer. Fmoc‐GGY‐OH (G: Gly, Y: Tyr, 10 mM) was incubated with tyrosinase (1.68 µM) in the phosphate buffer (0.2 M, pH 6.5) at 4 °C for 30 min, and then **2a** (100 mM) was added to the solution together with photo‐irradiation by 456 nm blue light (30 mW cm^−2^, Kessil Lamp) for 5 min at room temperature (**Figure** **3**A). Fmoc‐GGY‐OH peptide was quantitatively converted to a new peak in the HPLC chromatogram (Figure 3B). The kinetics of the reaction was measured by following the peak shift on the HPLC chromatograms at different time points (the end of the 30‐min tyrosinase incubation time was set as time zero). Tyrosinase‐oxidation resulted in a set of four peaks at time zero, and following the addition of vinyl ether, these peaks converted to a single peak within 10 s (Figure 3C). The apparent rate constant of the product formation, *k*_app_, was measured to be 0.06 s^−1^, and the second‐order rate constant *k*_2_ 6.0 M^−1^s^−1^ was equivalent to or higher than the reaction rate of strain‐promoted alkyne‐azide cycloaddition (SPAAC) reaction.^[^ ^72^ ^]^ The conjugation product showed high stability in both acidic and basic solutions. These data showed that the *o*‐quinone reaction could also take place in a peptide in an aqueous solution following enzymatic oxidation of terminal tyrosines.

Next, we expanded the substrate scope to peptides with different sequences and vinyl ether derivatives. First, we showed that catechol does not react with vinyl ether under visible light irradiation, indicating that *o*‐quinone (instead of catechol) is the substrate of the photoaddition reaction (Figure S2 in the Supporting Information). Lights of different wavelengths, such as 365, 405, and 456 nm, all promoted the reaction (Figure S3 in the Supporting Information). All the Tyr‐containing peptides can react, albeit giving different yields (Figure 3D, entries **4aa**–**4ad**, and Figures S4—S7 in the Supporting Information). However, CY‐containing dipeptide (Fmoc‐CY‐OH, C: Cys) cannot be oxidized by tyrosinase (Figure 3D, entry **4ae**, and Figure S8 in the Supporting Information), as Cys may inhibit or interfere with the activity of tyrosinase. The reaction was not affected by the presence of serine, lysine, methionine, or other amino acids (Figure 3D, entries **4af**–**4ah**, and Figures S9–S11 in the Supporting Information). We also explored the regioselectivity, i.e., the position of Tyr in peptides. Hexapeptides with Tyr at the N‐terminus or in the middle seem to show decreased product yields under the same condition (Figure 3D, entries **4ai**–**4ak**, and Figures S12–S14 in the Supporting Information), suggesting that the mushroom tyrosinase prefers C‐terminal Tyr residue, possibly due to steric hindrance. VE derivatives containing ethyl group, benzyl group, and butanol group, gave good yields (Figure 3E, entries **4da–4dc**, and Figures S15–S17 in the Supporting Information). The other end of the VE‐containing molecule can accommodate alkyne, azide, fluorescent groups, or biotin without jeopardizing the addition reaction (Figure 3E, entries **4dd**–**4dg**, and Figures S18–S21 in the Supporting Information). These results showcase the potential of this reaction in introducing fluorescent, affinity, or bioorthogonal reactive groups to Tyr‐containing synthetic peptides.

For the use of the chemoenzymatic photoaddition for protein modification, GFP with a Y‐tag (‐GGY) at the C‐terminus (GFP‐GGY) was used as a model protein. GFP‐GGY (1 *eq*.) was incubated with tyrosinase (0.01 *eq*.) and different Ves (100 *eq*.) in pH 6.5 phosphate buffer (PB) at 4 °C under 456 nm photo irradiation for 60 min. VE‐conjugated GFP‐GGY was detected by LC‐MS, showing a molecular weight increase that corresponds to the addition reaction. Under the same condition, **VE‐N_3_** was conjugated to GFP‐GGY successfully (Figures S22 and S23 in the Supporting Information). **VE‐biotin** also reacted with different proteins with the Y‐tag under the same reaction condition; no biotin‐conjugated product was formed if light or tyrosinase was absent, showing that light irradiation and tyrosinase are essential for this reaction. Next, the Y‐tag was genetically fused at the C‐terminus of a HER2‐specific nanobody (nbHER2) to give nbHER2‐GGY. NbHER2‐GGY can be readily biotinylated, but nbHER2‐GGF, with the C‐terminal Y in the Y‐tag mutated to Phe (F) was not biotinylated (Figures S22 and S23 in the Supporting Information). Besides this, VE‐conjugated and **VE‐N_3_**‐conjugated nbHER2‐GGY were analyzed by LC‐MS and LC‐MS/MS to confirm the reaction site was the C‐terminal Y (Figure S24–S31 in the Supporting Information). Notably, nbHER2 itself contains 6 Tyr in the sequence, but none of them seems to be the substrate of the chemoenzymatic reaction. This data shows that the chemoenzymatic tyrosine reaction could achieve a site‐selective and residue‐specific Tyr reaction at the Y‐tag in recombinant proteins.

### 2.3 Site‐Selective Tyr Reaction of IgGs

**Figure 4:**
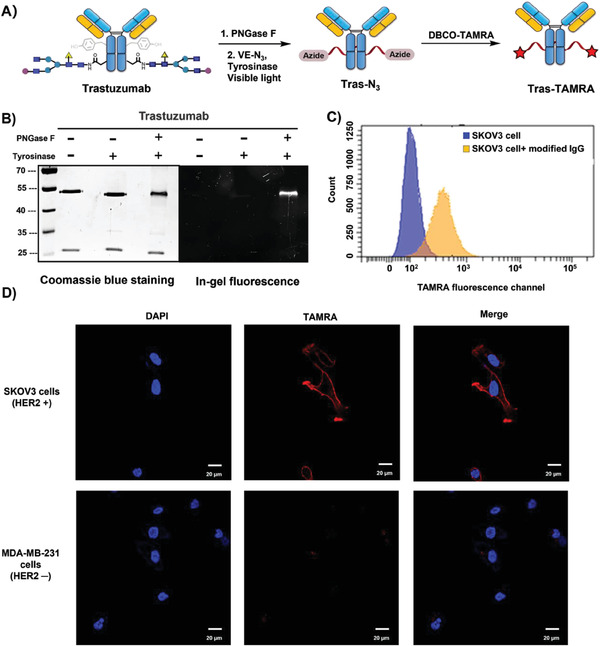
Selective modification of Tras. A) Schematic demonstration of the two‐step fluorescent labeling of Tras by **VE‐N_3_** and DBCO‐TAMRA. Reaction condition: 5.0 µM IgG (deglycosylated), 500 µM **VE‐N_3_**, 2.0 µM tyrosinase, PB buffer (0.2 M, pH 6.5), at 4 °C for 8 h with 456 nm irradiation. The reactions were quenched by 1% sodium dodecyl‐sulfate (SDS), and DBCO‐PEG_4_‐TAMRA was added at room temperature for 1.5 h. The solutions were then resolved by denaturing SDS−polyacrylamide gel electrophoresis (SDS‐PAGE) and imaged by in‐gel fluorescence scanning and Coomassie blue staining. B) SDS‐PAGE analysis of labeled Tras‐TAMRA. Left, Coomassie stain; right, fluorescent image, Ex, 365 nm. The raw figure can be found in Figure S39 in the Supporting Information. C) Flow cytometric analysis of SKOV3 cells incubated with Tras‐TAMRA. Briefly, SKOV3 cells were incubated with the PBS solution containing 200 nM Tras‐TAMRA for 30 min at room temperature before analysis. D) Fluorescent images of SKOV3 cells labeled with Tras‐TAMRA. Scale bar, 20 µm.

The chemical functionalization of IgGs brought new therapeutics for treating various diseases. For example, antibody‐drug conjugates (ADCs) with toxins (payloads) attached to IgGs through various bioconjugation techniques have emerged as a new class of clinical drugs for targeted cancer treatment.^[^ ^73^, ^74^ ^]^ Site‐selective functionalization of IgGs at the Fc domain is considered optimal as it avoids interfering with the antigen‐binding regions. We next subjected Trastuzumab (Tras), a therapeutic monoclonal antibody clinically used to treat breast cancer and stomach cancer under the brand name Herceptin, to the photoenzymatic reaction with **VE‐N_3_**. Prior to the reaction, Tras was pretreated with PNGase F enzyme to remove the N297 glycan to expose Y296 in the Fc‐domain, because this region is known to be highly accessible to tyrosinases.^[^ ^64^, ^65^ ^]^ Deglycosylated Tras was incubated with **VE‐N_3_** (100 *eq*.) and tyrosinase (0.4 *eq*.) at 4 °C for 8 h under 456 nm blue light irradiation (**Figure** **4**A). After installing the azide group, we introduced a fluorophore, 6‐carboxytetramethylrhodamine (TAMRA)**_,_** through a SPAAC reaction between DBCO and N_3_; the photoaddition reaction thus can be confirmed by the covalent linkage of a fluorescent dye. SDS‐PAGE analysis showed that the heavy chain of Tras was successfully labeled with DBCO‐TAMRA, but not the light chain (Figure 4B). The same reaction occured to Atezolizumab (Atezo), Daratumumab, and Cetuximab (Atezo did not have the N297 glycan, but the latter two needed to be deglycosylated before the reaction) (Figures S32 and S33 in the Supporting Information). Kinetic analysis showed that the modification reaction reached 50% in 16 h (Figure S34 in the Supporting Information). The chemical modification did not cause significant degradation or aggregation of the antibody (Figure S35 in the Supporting Information). Based on an ELISA assay, Tras and Tras‐VE‐N_3_ bound with the antigen with EC_50_ values of 0.06 and 0.21 nM, respectively, suggesting that chemical modification caused only a slight loss of the binding affinity (Figure S36 in the Supporting Information). Notwithstanding, Tras‐TARMA effectively labeled the HER2‐positive SKOV3 cells but not the HER2‐negative MDA‐MB‐231 cells (Figures 4C and D). LC‐MS/MS analysis of the digested fragments of the labeled Atezo showed that the reaction occurred at Y296, echoing the requirement of deglycosylation of N297 (Figures S37 and S38 in the Supporting Information). Altogether, these results show that chemoenzymatic photoaddition reaction can site‐selectively label therapeutic antibodies at Y296 (reactions at other Tyr residues were not detected), providing a site‐selective way to functionalize IgGs.

### 2.4 Monovalent Tras‐Vc‐MMAE ADC

**Figure 5:**
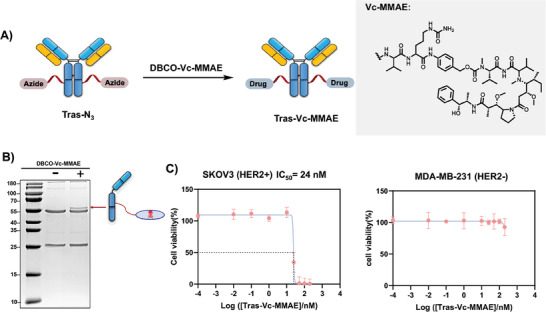
Synthesis and cytotoxicity of site‐selective Tras‐Vc‐MMAE. A) Synthetic scheme. B) SDS‐PAGE analysis of the Tras mixture after conjugation reaction. C) Concentration‐dependent cytotoxicity of Tras‐Vc‐MMAE to HER2+ SKOV3 cells and HER2‐ MDA‐MB‐231 cells. Note here [Tras‐Vc‐MMAE] represents the concentration of the total Tras in the mixture of antibodies, and Tras‐Vc‐MMAE is only a fraction of the total concentration.

The formation of monovalent Tras‐N_3_ motivated us to construct a monovalent ADC by further derivatizing the azide group. Monomethyl‐auristatin E (MMAE), a potent anti‐mitotic agent commonly, was chosen as the cytotoxic drug to be conjugated with Tras‐N_3_.^[^ ^75^, ^76^, ^77^, ^78^, ^79^ ^]^ A functionalized MMAE, **DBCO‐Vc‐MMAE**, was synthesized. A dipeptide linker, valine‐citrulline (Vc), was inserted between the azide‐reactive DBCO group and MMAE. The Vc linker can be selectively cleavaged by cathepsin B to release MMAE.^[^ ^80^ ^]^ Tras‐Vc‐MMAE was then synthesized by reacting Tras‐N_3_ with **DBCO‐Vc‐MMAE** by incubating both reactants at room temperature for 1 h (**Figure** **5**A). Excess **DBCO‐Vc‐MMAE** was removed by a desalting column. In SDS‐PAGE gel, a band slightly higher than the heavy chain, indicating that **DBCO‐Vc‐MMAE** was covalently tethered to the heavy chain only and likely only one Vc‐MMAE molecule was attached (Figure 5B). The cellular cytotoxicity of HER2‐specific ADC was subsequently evaluated in MDA‐MB‐231 cells (HER2 antigen negative) and SKOV3 cells (HER2 antigen positive). Tras‐Vc‐MMAE significantly reduced the viability of SKOV3 cells at the total Tras concentration as low as 30 nM (IC_50_ ≈24 nM, calculated based on the total Tras concentration), whereas it did not cause noticeable toxicity to MDA‐MB‐231 cells at concentrations as high as 200 nM (Figure 5C). Tras antibody alone did not cause toxicity (Figure S40 in the Supporting Information). Notably, the listed concentrations are those of the total Tras antibody, a mixture including Tras‐Vc‐MMAE, unreacted Tras‐N_3_, and unreacted Tras. Estimably, the Tras‐Vc‐MMAE population constitutes ≈24% of the total Tras antibody populations, based on the band thickness in Figure 5B.

### 2.5 Antibody‐Cell Conjugation for Immunotherapy

**Figure 6:**
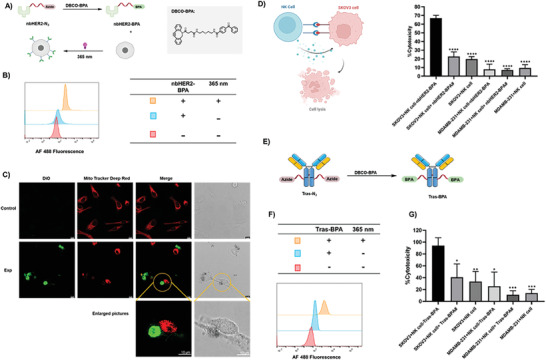
Antibody‐cell conjugation enhanced ADCC. A) Synthetic route toward nbHER2‐BPA. B) Flow cytometry results of UV light‐induced conjugation of nbHER2‐BPA/AF 488 to THP1 cells. C) Confocal images showing the interaction of THP1‐nbHER2 with HER2(+) SKOV3. D) Cell cytotoxicity promoted by the cell‐cell interaction. E) Synthetic route toward Tras‐BPA. F) Flow cytometry results of UV light‐induced conjugation of Tras‐BPA/AF 488 to THP1 cells. G) Cell cytotoxicity promoted by the cell‐cell interaction. Label # indicates groups that did not receive light irradiation. Data are presented as the mean ± s.d. of n = 3 independent experiments. *, P <0.05. **, P <0.01. ***, P < 0.001.****, P <0.0001.

Lastly, site‐selectively modified nanobody or antibody were used to covalently conjugate with immune effector cells. HER2‐specific nanobody nbHER2 with a C‐terminal Y‐tag GGY first underwent the tyrosine‐selective reaction to give nbHER2‐N_3_, which was subsequently converted to nbHER2‐BPA (BPA represents benzophenone‐3‐carboxylic acid) by reacting with a bifunctional DBCO‐BPA molecule to conjugate the photocrosslinker BPA at the C‐terminus of the nanobody. Then nbHER2‐BPA was fluorescently labeled with Alexa Fluor 488‐NHS ester to give nbHER2‐BPA/AF488. THP1 cells, a human leukemia monocytic cell line with features of macrophages, were incubated with 20 µM nbHER2‐BPA/AF488 and irradiated by a 365‐nm UV light at 20 mW cm^−2^ for 20 min (**Figure** **6**A). After extensive washing to remove unreacted reagents, THP1 cells were analyzed by flow cytometry. Compared to cells without treatment by nbHER2‐BPA/AF488, or treated with nbHER2‐BPA/AF488 but no light irradiation, photocrosslinking of nbHER2‐BPA/AF488 led to fluorescent labeling of THP1 cells (Figure 6B). Next, nbHER2‐BPA‐conjugated THP1 cells (labeled with the green dye DIO) were mixed and incubated with HER2‐positive SKOV3 cells (labeled with the red dye Mito Tracker Red) for 8 hours. THP1 cells without conjugation with nbHER2‐BPA were used as a control. Because SKOV3 cells are adherent and THP1 cells suspend in the culture, after washing, no THP1 cells could be found on the coverslip in the control group. In contrast, nbHER2‐BPA‐conjugated THP1 cells (nbHER2‐THP1) attached to SKOV3 cells, showing that nbHER2 conjugated on the surface of THP1 cells guided THP1 to SKOV3 and formed contacts between the two types of cells (Figure 6C). Next, we utilized the same protocol to covalently conjugate nbHER2‐BPA with NK‐92 cells, a type of interleukin‐2 (IL‐2)‐dependent natural killer cell line that has cellular cytotoxicity, but without targeting capability. Based on the release of lactate dehydrogenase (LDH), at an effector cells/target cells (E/T) ratio of 2:1, nbHER2‐BPA‐conjugated NK‐92 cells (nbHER2‐NK‐92) showed significantly higher cytotoxicity than NK‐92 cells without conjugation with nbHER2 (Figure 6D). Such an enhanced ADCC effect was found to be HER2‐dependent, because HER2‐negative MDA‐MB‐231 cells were not killed by nbHER2‐NK‐92 cells. Furthermore, we applied the same reaction to HER2‐specific mAb Trastuzumab. Site‐selectively functionalized Tras‐BPA/AF488 conjugates to THP1 cells, as shown in the flow cytometry experiment (Figures 6E and F). Tras‐BPA‐conjugated NK‐92 cells (Tras‐NK‐92) also showed significantly higher cytotoxicity to SK‐OV‐3 cells than NK‐92 cells without conjugation with Tras (Figure 6G). We also synthesized a randomly conjugated version, Tras‐BPA (random) by reacting the Trastuzumab antibody with an NHS‐N_3_ bifunctional molecule, followed by reacting with a DBCO‐BPA molecule. Comparing with Tras‐BPA(random)‐conjugated NK‐92 cells, NK‐92 cells conjugated with site‐selectively modified Tras‐BPA showed equivalent cytotoxicity (Figure S41 in the Supporting Information). Taken together, our experiments show that covalent conjugation of nanobodies or antibodies, enabled by the site‐selective tyrosine reaction, to immune effector cells promoted the contact between immune effector cells and the target cancer cells, and enhanced the killing of the cancer cells.

## 3 Conclusion

Our study presents a site‐selective, efficient, and mild Tyr reaction coupling the tyrosinase oxidation and visible‐light‐induced [3+2] photoaddition reaction. This conjugation reaction has outstanding chemo‐ and site‐selectivity and functional group tolerance, allowing for site‐selective functionalization of peptides, recombinant proteins, and IgGs. The reaction rate is comparable to the traditional SPAAC reaction. The conjugate linkage is stable in physiological, acidic, and basic solutions. Bruins et al. showed that after deglycosylation by PNGase F, up to two Tyr residues can be oxidized by tyrosinase.^[^ ^64^, ^65^ ^]^ Here, we showed that only Y296 was modified in our method according to extensive mass spectrometric analysis.

Site‐selective monofunctionalization of IgGs enables the construction of ADCs carrying a single cytotoxic drug at the heavy chain for targeted cancer therapy.^[^ ^78^, ^79^ ^]^ The synthesis of ADCs is mostly based on cysteine reactions and often lacks control of the reaction sites or stoichiometry. ADCs with a 1:1 antibody‐to‐drug ratio at the residue‐specific location would reduce the heterogeneity of the product and may give better pharmacokinetics in vivo. Compared with multivalent ADCs, which showed an IC_50_ of 72 pM,^[^ ^79^ ^]^ the monovalent Tras‐Vc‐MMAE we synthesized seemed to have lower cytotoxicity (24 nM based on total Tras). However, as Tras‐Vc‐MMAE only constitutes 24% of the total Tras in the mixture added to the cells, the actual toxicity of Tras‐Vc‐MMAE may be underestimated. Considering the number of MMAE molecules in the multivalent Tras‐Vc‐MMAE, we estimate that for the activity of each MMAE molecule, our monovalent Tras‐Vc‐MMAE is equivalent to the multivalent counterpart.^[^ ^65^, ^79^ ^]^ We also demonstrated the application of this reaction in antibody‐cell conjugation: Covalent conjugation of monofunctionalized nanobodies and antibodies to immune effector cells enables precise control of the orientation of these antigen‐binding molecules on the cell surface, thus ensuring maximizing the contact between immune effector cells and target cancer cells. Comparing the site‐selectively functionalized Tras‐BPA and randomly functionalized Tras‐BPA (random), we showed that they had equivalent activity to functionalize NK‐92 cells (Figure S41 in the Supporting Information). Compared to the commonly used cysteine conjugation strategy in ADC preparation, the Tyr conjugation reaction based on our chemoenzymatic method provides an alternative, which yields homogenous populations of ADCs without compromising the therapeutic effects. A limitation of the current method is the modest yield, which needs to be improved through optimization. Taken together, here we demonstrate a chemoenzymatic photoaddition reaction for Tyr derivatization, site‐selective Tyr reactions in tagged recombinant proteins and native IgGs, the construction of monovalent ADCs, and antibody functionalization of immune effector cells for targeted cancer immunotherapy.

## Conflict of Interest

The authors declare no conflict of interest.

## Author Contributions

H.C. and J.X. performed conceptualization; H.C., J.Q., B.L., D.Y., H.K., Y.B., Y.Z., Z.X. performed methodology, data curation; H.C. wrote the original draft preparation. H.‐C.F.W. and Y.‐L.S.T. performed calculation; J.X. performed supervision. J.X. wrote ‐ review and edited the final draft.

## Supporting information

Supporting Information

## Data Availability

The data that support the findings of this study are available in the supplementary material of this article.

## References

[1] A. F. U. H. Saeed , R. Wang , S. Ling , S. Wang , Front Microbiol. 2017, 8, 495. 28400756 10.3389/fmicb.2017.00495 PMC5368232

[2] S. Zinn , R. Vazquez‐Lombardi , C. Zimmermann , P. Sapra , L. Jermutus , D. Christ , Nat. Cancer. 2023, 4, 165. 36806801 10.1038/s43018-023-00516-z

[3] S. Jin , Y. Sun , X. Liang , X. Gu , J. Ning , Y. Xu , S. Chen , L. Pan , Sig. Transduct. Target. Ther. 2022, 7, 39. 10.1038/s41392-021-00868-x PMC8821599 35132063

[4] R. S. Goydel , C. Rader , Oncogene 2021, 40, 3655. 33947958 10.1038/s41388-021-01811-8 PMC8357052

[5] A. M. Scott , J. D. Wolchok , L. J. Old , Nat. Rev. Cancer. 2012, 12, 278. 22437872 10.1038/nrc3236

[6] V. R. Gómez Román , J. C. Murray , L. M. Weiner , in Antibody Fc: Linking Adaptive and Innate Immunity (Eds: M. E. Ackerman , F. Nimmerjahn ), Academic Press, Cambridge 2014, 1.

[7] J. U. Igietseme , X. Zhu , C. M. Black , in Antibody Fc: Linking Adaptive and Innate Immunity (Eds: M. E. Ackerman , F. Nimmerjahn ), Academic Press, Cambridge 2014, 269.

[8] L. Gauthier , A. Morel , N. Anceriz , B. Rossi , A. Blanchard‐Alvarez , G. Grondin , S. Trichard , C. Cesari , M. Sapet , F. Bosco , H. Rispaud‐Blanc , F. Guillot , S. Cornen , A. Roussel , B. Amigues , G. Habif , F. Caraguel , S. Arrufat , R. Remark , F. Romagné , Y. Morel , E. Narni‐Mancinelli , E. Vivier , Cell 2019, 177, 1701. 31155232 10.1016/j.cell.2019.04.041

[9] X. Xiao , Y. Cheng , X. Zheng , Y. Fang , Y. Zhang , R. Sun , Z. Tian , H. Sun , Front. Immunol. 2023, 14, 10.3389/fimmu.2023.1113303. PMC10126364 37114050

[10] M.‐E. Goebeler , R. C. Bargou , Nat. Rev. Clin. Oncol. 2020, 17, 418. 32242094 10.1038/s41571-020-0347-5

[11] R. C. Sterner , R. M. Sterner , Blood Cancer J. 2021, 11, 69. 33824268 10.1038/s41408-021-00459-7 PMC8024391

[12] M. C. Milone , J. Xu , S.‐J. Chen , M. A. Collins , J. Zhou , D. J. Powell , J. J. Melenhorst , Nat. Cancer. 2021, 2, 780. 34485921 10.1038/s43018-021-00241-5 PMC8412433

[13] J. C. Maza , D. M. García‐Almedina , L. E. Boike , N. X. Hamlish , D. K. Nomura , M. B. Francis , ACS Cent. Sci. 2022, 8, 955. 35912347 10.1021/acscentsci.1c01265 PMC9335918

[14] A. P. R. Johnston , M. M. J. Kamphuis , G. K. Such , A. M. Scott , E. C. Nice , J. K. Heath , F. Caruso , ACS Nano 2012, 6, 6667. 22872125 10.1021/nn3010476

[15] C. D. Phung , T. T. Pham , H. T. Nguyen , T. T. Nguyen , W. Ou , J.‐H. Jeong , H.‐G. Choi , S. K. Ku , C. S. Yong , J. O. Kim , Acta Biomater. 2020, 115, 371. 32798721 10.1016/j.actbio.2020.08.008

[16] M. J. Frank , N. Olsson , A. Huang , S.‐W. Tang , R. S. Negrin , J. E. Elias , E. H. Meyer , Cytotherapy 2020, 22, 135. 32171435 10.1016/j.jcyt.2020.01.003

[17] H.‐K. Li , C.‐W. Hsiao , S.‐H. Yang , H.‐P. Yang , T.‐S. Wu , C.‐Y. Lee , Y.‐L. Lin , J. Pan , Z.‐F. Cheng , Y.‐D. Lai , S.‐C. Hsiao , Cancers 2021, 13, 2724. 34072864 10.3390/cancers13112724 PMC8199224

[18] R. Tang , Y.‐H. Fu , B. Gong , Y.‐Y. Fan , H.‐H. Wang , Y. Huang , Z. Nie , P. Wei , Angew. Chem., Int. Ed. 2022, 61, e202205902. 10.1002/anie.202205902 35751134

[19] J. Li , M. Chen , Z. Liu , L. Zhang , B. H. Felding , K. W. Moremen , G. Lauvau , M. Abadier , K. Ley , P. Wu , ACS Cent. Sci. 2018, 4, 1633. 30648147 10.1021/acscentsci.8b00552 PMC6311947

[20] E. M. Sletten , C. R. Bertozzi , Angew. Chem., Int. Ed. 2009, 48, 6974. 10.1002/anie.200900942 PMC2864149 19714693

[21] O. Boutureira , G. J. L. Bernardes , Chem. Rev. 2015, 115, 2174. 25700113 10.1021/cr500399p

[22] J. M. Chalker , G. J. L. Bernardes , Y. A. Lin , B. G. Davis , Chem. Asian. J. 2009, 4, 630. 19235822 10.1002/asia.200800427

[23] S. B. Gunnoo , A. Madder , ChemBioChem 2016, 17, 529. 26789551 10.1002/cbic.201500667

[24] I. Dovgan , S. Erb , S. Hessmann , S. Ursuegui , C. Michel , C. Muller , G. Chaubet , S. Cianférani , A. Wagner , Org. Biomol. Chem. 2018, 16, 1305. 29388667 10.1039/c7ob02844j

[25] Y. Seki , T. Ishiyama , D. Sasaki , J. Abe , Y. Sohma , K. Oisaki , M. Kanai , J. Am. Chem. Soc. 2016, 138, 10798. 27534812 10.1021/jacs.6b06692

[26] M. T. Taylor , J. E. Nelson , M. G. Suero , M. J. Gaunt , Nature 2018, 562, 563. 30323287 10.1038/s41586-018-0608-y PMC6203954

[27] S. Lin , X. Yang , S. Jia , A. M. Weeks , M. Hornsby , P. S. Lee , R. V. Nichiporuk , A. T. Iavarone , J. A. Wells , F. D. Toste , C. J. Chang , Science 2017, 355, 597. 28183972 10.1126/science.aal3316 PMC5827972

[28] D. Alvarez Dorta , D. Deniaud , M. Mével , S. G. Gouin , Chem.‐Eur. J. 2020, 26, 14257. 32538529 10.1002/chem.202001992

[29] J. Dong , L. Krasnova , M. G. Finn , K. B. Sharpless , Angew. Chem., Int. Ed. 2014, 53, 9430. 10.1002/anie.201309399 25112519

[30] F. W. Kimani , J. C. Jewett , Angew. Chem., Int. Ed. 2015, 54, 4051. 10.1002/anie.201411277 25663253

[31] H. G. Higgins , K. J. Harrington , Arch. Biochem. Biophys. 1959, 85, 409. 14401754 10.1016/0003-9861(59)90506-5

[32] J. Gavrilyuk , H. Ban , M. Nagano , W. Hakamata , C. F. Barbas , Bioconjugate Chem. 2012, 23, 2321. 10.1021/bc300410p PMC3526679 23181702

[33] H.‐M. Guo , M. Minakawa , L. Ueno , F. Tanaka , Bioorg. Med. Chem. Lett. 2009, 19, 1210. 19136260 10.1016/j.bmcl.2008.12.071 PMC2643309

[34] D. Ma , Y. Xie , J. Zhang , D. Ouyang , L. Yi , Z. Xi , Chem. Commun. 2014, 50, 15581. 10.1039/c4cc07057g 25360457

[35] N. S. Joshi , L. R. Whitaker , M. B. Francis , J. Am. Chem. Soc. 2004, 126, 15942. 15584710 10.1021/ja0439017

[36] J. M. Mcfarland , N. S. Joshi , M. B. Francis , J. Am. Chem. Soc. 2008, 130, 7639. 18498164 10.1021/ja710927q

[37] M. Minakawa , H.‐M. Guo , F. Tanaka , J. Org. Chem. 2008, 73, 8669. 18844415 10.1021/jo8017389 PMC2647331

[38] J. Gavrilyuk , H. Ban , H. Uehara , S. J. Sirk , K. Saye‐Francisco , A. Cuevas , E. Zablowsky , A. Oza , M. S. Seaman , D. R. Burton , C. F. Barbas , J. Virol. 2013, 87, 4985. 23427154 10.1128/JVI.03146-12 PMC3624287

[39] P. A. Szijj , K. A. Kostadinova , R. J. Spears , V. Chudasama , Org. Biomol. Chem. 2020, 18, 9018. 33141139 10.1039/d0ob01912g PMC7814872

[40] J. Zhang , D. Ma , D. Du , Z. Xi , L. Yi , Org. Biomol. Chem. 2014, 12, 9528. 25354584 10.1039/c4ob01873g

[41] M. H. Y. Cheng , H. Savoie , F. Bryden , R. W. Boyle , Photochem. Photobiol. Sci. 2017, 16, 1260. 28636039 10.1039/c7pp00091j

[42] N. Griebenow , S. Greven , M. Lobell , A. M. Dilmaç , S. Bräse , RSC Adv. 2015, 5, 103506.

[43] D. Alvarez‐Dorta , C. Thobie‐Gautier , M. Croyal , M. Bouzelha , M. Mével , D. Deniaud , M. Boujtita , S. G. Gouin , J. Am. Chem. Soc. 2018, 140, 17120. 30422648 10.1021/jacs.8b09372

[44] C. Song , K. Liu , Z. Wang , B. Ding , S. Wang , Y. Weng , C.‐W. Chiang , A. Lei , Chem. Sci. 2019, 10, 7982. 31673320 10.1039/c9sc02218j PMC6788519

[45] S. Sato , H. Nakamura , Angew. Chem., Int. Ed. 2013, 52, 8681. 10.1002/anie.201303831 23824878

[46] T. Long , L. Liu , Y. Tao , W. Zhang , J. Quan , J. Zheng , J. D. Hegemann , M. Uesugi , W. Yao , H. Tian , H. Wang , Angew. Chem., Int. Ed. 2021, 60, 13414. 10.1002/anie.202102287 33847040

[47] M. Shinoda , T. Isozaki , T. Suzuki , Photochem. Photobiol. 2014, 90, 92. 23998294 10.1111/php.12168

[48] L. O. Reid , M. Vignoni , N. Martins‐Froment , A. H. Thomas , M. L. Dántola , Photochem. Photobiol. Sci. 2019, 18, 1732. 31070216 10.1039/c9pp00182d

[49] S. D. Glover , C. Jorge , L. Liang , K. G. Valentine , L. Hammarström , C. Tommos , J. Am. Chem. Soc. 2014, 136, 14039. 25121576 10.1021/ja503348d PMC4195373

[50] B. X. Li , D. K. Kim , S. Bloom , R. Y.‐C. Huang , J. X. Qiao , W. R. Ewing , D. G. Oblinsky , G. D. Scholes , D. W. C. Macmillan , Nat. Chem. 2021, 13, 902. 34183819 10.1038/s41557-021-00733-y

[51] S. Ito , T. Kato , K. Shinpo , K. Fujita , Biochem. J. 1984, 222, 407. 6433900 10.1042/bj2220407 PMC1144193

[52] A. Borrmann , O. Fatunsin , J. Dommerholt , A. M. Jonker , D. W. P. M. Löwik , J. C. M. Van Hest , F. L. Van Delft , Bioconjugate Chem. 2015, 26, 257. 10.1021/bc500534d 25521043

[53] A. M. Jonker , A. Borrmann , E. R. H. Van Eck , F. L. Van Delft , D. W. P. M. Löwik , J. C. M. Van Hest , Adv. Mater. 2015, 27, 1235. 25535032 10.1002/adma.201404448

[54] J. Dommerholt , S. Schmidt , R. Temming , L. J. A. Hendriks , F. P. J. T. Rutjes , J. C. M. Van Hest , D. J. Lefeber , P. Friedl , F. L. Van Delft , Angew. Chem., Int. Ed. 2010, 49, 9422. 10.1002/anie.201003761 PMC3021724 20857472

[55] J. J. Bruins , A. H. Westphal , B. Albada , K. Wagner , L. Bartels , H. Spits , W. J. H. Van Berkel , F. L. Van Delft , Bioconjugate Chem. 2017, 28, 1189. 10.1021/acs.bioconjchem.7b00046 PMC5399473 28263569

[56] E. Montanari , A. Gennari , M. Pelliccia , L. Manzi , R. Donno , N. J. Oldham , A. Macdonald , N. Tirelli , Bioconjugate Chem. 2018, 29, 2550. 10.1021/acs.bioconjchem.8b00227 29975838

[57] A. M. Marmelstein , M. J. Lobba , C. S. Mogilevsky , J. C. Maza , D. D. Brauer , M. B. Francis , J. Am. Chem. Soc. 2020, 142, 5078. 32093466 10.1021/jacs.9b12002

[58] A.‐W. Struck , M. R. Bennett , S. A. Shepherd , B. J. C. Law , Y. Zhuo , L. S. Wong , J. Micklefield , J. Am. Chem. Soc. 2016, 138, 3038. 26867114 10.1021/jacs.5b10928

[59] T. Akio , S. Miho , Chem. Lett. 1989, 18, 9.

[60] M. J. Lobba , C. Fellmann , A. M. Marmelstein , J. C. Maza , E. N. Kissman , S. A. Robinson , B. T. Staahl , C. Urnes , R. J. Lew , C. S. Mogilevsky , J. A. Doudna , M. B. Francis , ACS Cent. Sci. 2020, 6, 1564. 32999931 10.1021/acscentsci.0c00940 PMC7517114

[61] J. C. Maza , D. M. García‐Almedina , L. E. Boike , N. X. Hamlish , D. K. Nomura , M. B. Francis , ACS Cent. Sci. 2022, 8, 955. 35912347 10.1021/acscentsci.1c01265 PMC9335918

[62] J. J. Bruins , D. Blanco‐Ania , V. Van Der Doef , F. L. Van Delft , B. Albada , Chem. Commun. 2018, 54, 7338. 10.1039/c8cc02638f 29911239

[63] J. J. Bruins , C. Van De Wouw , K. Wagner , L. Bartels , B. Albada , F. L. Van Delft , ACS Omega 2019, 4, 11801. 31460288 10.1021/acsomega.9b01727 PMC6682001

[64] G. P. Subedi , A. W. Barb , Structure 2015, 23, 1573. 26211613 10.1016/j.str.2015.06.015 PMC4558368

[65] J. J. Bruins , J. A. M. Damen , M. A. Wijdeven , L. P. W. M. Lelieveldt , F. L. Van Delft , B. Albada , Bioconjugate Chem. 2021, 32, 2167. 10.1021/acs.bioconjchem.1c00351 PMC8532111 34519477

[66] J. Li , H. Kong , L. Huang , B. Cheng , K. Qin , M. Zheng , Z. Yan , Y. Zhang , J. Am. Chem. Soc. 2018, 140, 14542. 30351919 10.1021/jacs.8b08175

[67] V. R. Kumar , N. Rajkumar , F. Ariese , S. Umapathy , J. Phys. Chem. A. 2015, 119, 10147. 26381591 10.1021/acs.jpca.5b07972

[68] Y. Honda , M. Hada , M. Ehara , H. Nakatsuji , Phys. Chem. A. 2007, 111, 2634. 10.1021/jp0686487 17388344

[69] J. Makuraza , T. Pogrebnaya , A. Pogrebnoi , Int. J. Mater. Sci. Appl. 2015, 4, 314.

[70] H. Shimoishi , K. Akiyama , S. Tero‐Kubota , Y. Ikegami , Chem. Lett. 1988, 17, 251.

[71] M. Fréneau , N. Hoffmann , J. Photochem. Photobiol. C. 2017, 33, 83.

[72] M. F. Debets , S. S. Van Berkel , J. Dommerholt , A. (T.) J. Dirks , F. P. J. T. Rutjes , F. L. Van Delft , Acc. Chem. Res. 2011, 44, 805. 21766804 10.1021/ar200059z

[73] R.‐M. Lu , Y.‐C. Hwang , I.‐J. Liu , C.‐C. Lee , H.‐Z. Tsai , H.‐J. Li , H.‐C. Wu , J. Biomed. Sci. 2020, 27, 1. 31894001 10.1186/s12929-019-0592-z PMC6939334

[74] P. Holliger , P. J. Hudson , Nat. Biotechnol. 2005, 23, 1126. 16151406 10.1038/nbt1142

[75] V. Chudasama , A. Maruani , S. Caddick , Nat. Chem. 2016, 8, 114. 26791893 10.1038/nchem.2415

[76] S. Mariathasan , M.‐W. Tan , Trends. Mol. Med. 2017, 23, 135. 28126271 10.1016/j.molmed.2016.12.008

[77] N. Ashman , J. D. Bargh , D. R. Spring , Chem. Soc. Rev. 2022, 51, 9182. 36322071 10.1039/d2cs00446a

[78] M.‐A. Kasper , A. Stengl , P. Ochtrop , M. Gerlach , T. Stoschek , D. Schumacher , J. Helma , M. Penkert , E. Krause , H. Leonhardt , C. P. R. Hackenberger , Angew. Chem., Int. Ed. 2019, 58, 11631. 10.1002/anie.201904193 PMC6851832 31250955

[79] C. E. Stieger , Y. Park , M. A. R. De Geus , D. Kim , C. Huhn , J. S. Slenczka , P. Ochtrop , J. M. Müchler , R. D. Süssmuth , J. Broichhagen , M.‐H. Baik , C. P. R. Hackenberger , Angew. Chem., Int. Ed. 2022, 61, e202205348. 10.1002/anie.202205348 PMC9804898 35792701

[80] J. D. Bargh , A. Isidro‐Llobet , J. S. Parker , D. R. Spring , Chem. Soc. Rev. 2019, 48, 4361. 31294429 10.1039/c8cs00676h

