# Supplementary material for: Site‐Selective Tyrosine Reaction for Antibody‐Cell Conjugation and Targeted Immunotherapy
Source: Adv Sci (Weinh). 2023 Dec 3;11(5):2305012. doi: 10.1002/advs.202305012 (PMC10837340; doi:10.1002/advs.202305012)
Supplement: Supplementary file 1 — Supporting Information [file ADVS-11-2305012-s001.pdf]

ADVANCED SCIENCE
Open Access

Supporting Information

for *Adv. Sci.*, DOI 10.1002/advs.202305012

Site-Selective Tyrosine Reaction for Antibody-Cell Conjugation and Targeted Immunotherapy

Hongfei Chen, Hong-Chai Fabio Wong, Jiaming Qiu, Biquan Li, Dingdong Yuan, Hao Kong, Yishu Bao, Yu Zhang, Zhiyi Xu, Ying-Lung Steve Tse and Jiang Xia**

Supporting Information

Site-Selective Tyrosine Reaction for Antibody-Cell Conjugation and Targeted Immunotherapy

Hongfei Chen, Hong-Chai Fabio Wong, Jiaming Qiu, Biquan Li, Dingdong Yuan, Hao Kong, Yishu Bao, Yu Zhang, Zhiyi Xu, Ying-Lung Steve Tse,* Jiang Xia*

Department of Chemistry and Center for Cell & Developmental Biology, The Chinese University of Hong Kong, Shatin, Hong Kong SAR, China.

*Address correspondence to stevetse@cuhk.edu.hk and jiangxia@cuhk.edu.hk

Phone: (852) 3943 6165

Fax: (852) 2603 5057

Contents

Items	Page No.
**Detailed experimental procedures**	**S5**
**Figure S1.** The model reaction between **1a** and **2a**.	**S29**
**Figure S2.** Catechol and vinyl ether did not react.	**S30**
**Figure S3.** Photoaddition reactions under different wavelengths of light.	**S31**
**Figure S4**. Reaction between Fmoc-Y-OH and vinyl ether.	**S32**
**Figure S5.** Reaction between Fmoc-FY-OH and vinyl ether.	**S33**
**Figure S6**. Reaction between Fmoc-GY-OH and vinyl ether.	**S34**
**Figure S7**. Reaction between Fmoc-GGY-OH and vinyl ether.	**S35**
**Figure S8.** Fmoc-CY-OH did not react with vinyl ether.	**S36**
**Figure S9**. Reaction between Fmoc-SGY-OH and vinyl ether.	**S37**
**Figure S10**. Reaction between Fmoc-KGY-OH and vinyl ether.	**S38**
**Figure S11**. Reaction between Fmoc-MGY-OH and vinyl ether.	**S39**
**Figure S12**. Reaction between Fmoc-GGSGGY-OH and vinyl ether.	**S40**
**Figure S13**. Reaction between Fmoc-GGSYGG-OH and vinyl ether.	**S41**
**Figure S14.** Reaction between Fmoc-YGGSGG-OH and vinyl ether.	**S42**
**Figure S15**. Reaction analysis and yield of **4da**.	**S43**
**Figure S16.** Reaction analysis and yield of **4db**.	**S44**
**Figure S17.** Reaction analysis and yield of **4dc**.	**S45**
**Figure S18.** Reaction analysis and yield of **4dd**.	**S46**

**Figure S19.** Reaction analysis and yield of **4de**.	**S47**
**Figure S20.** Reaction analysis and yield of **4df**.	**S48**
**Figure S21.** Reaction analysis and yield of **4dg**.	**S49**
**Figure S22.** Tyrosine reaction for the functionalization of recombinant proteins.	**S50**
**Figure S23.** Protein modification of MBP-GGY, GFP-GGY, and nbHER2-GGY.	**S51**
**Figure S24.** Characterization of nbHER2-GGY.	**S53**
**Figure S25.** Modification and characterization of nbHER2-GGY with vinyl ether.	**S54**
**Figure S26.** LC-MS/MS characterization of the reaction site of nbHER2-GGY-VE.	**S55**
**Figure S27.** Modification and characterization of nbHER2-GGY with vinyl ether azide.	**S57**
**Figure S28.** MS analysis of GFP-GGY.	**S58**
**Figure S29.** MS analysis of the reaction product of GFP-GGY with vinyl ether.	**S59**
**Figure S30.** Modification and characterization of GFP-GGY with vinyl ether azide.	**S60**
**Figure S31.** LC-MS/MS analysis of GFP-GGY-VE-N_3	**S61**
**Figure S32.** Sequence comparison of human IgG1 heavy chains.	**S62**
**Figure S33.** Heavy chain modification of Human IgG1.	**S64**
**Figure S34.** Kinetics of the antibody reaction.	**S66**
**Figure S35.** Chemical modification did not cause significant degradation or aggregation the antibody.	**S67**
**Figure S36.** Antigen binding ability of antibodies by ELISA.	**S68**
**Figure S37.** MALDI-TOF MS analysis of enzyme-digested TAMRA-labeled Trastuzumab (Tras-VE-TAMRA).	**S69**
**Figure S38.** LC-MS/MS characterization of reaction site of Atezo-N_3.	**S70**

**Figure S39.** Raw gel figure of Figure 4B.	**S71**
**Figure S40.** Concentration dependent cellular cytotoxicity of the generated ADC and antibody.	**S72**
**Figure S41.** Cytotoxicity of antibodies with different modification methods**.**	**S73**
**Figure S42.** HR-ESI analysis of MMAE compounds.	**S74**
**Figure S43.** NMR spectra of VE-N_3.	**S75**
**Figure S44.** NMR spectrums of VE-alkyne.	**S76**
**Figure S45.** NMR spectrums of VE-Biotin.	**S77**
**Figure S46.** NMR spectrums of VE-FAM.	**S78**
**Figure S47.** NMR spectrums of Fmoc-Tyr-OH 3a.	**S79**
**Figure S48.** Structure confirmation of product 4aa.	**S80**
**Reference**	**S82**

Detailed experimental procedures

Materials and instruments. Unless otherwise noted, all reagents were purchased from commercial sources and used without further purification. Fmoc-protected amino acids and coupling reagents were obtained from GL Biochem Ltd. (Shanghai, China). Rink amide resins were obtained from Biotage (Uppsala, Sweden). 5(6)-TMARA was purchased from Beijing Okeanos Technology Co., Ltd. (Beijing, China). 3-(Vinyloxy)-1-propylamine, trifluoroacetic acid, and triisopropylsilane were purchased from J&K Scientific Ltd. (Beijing, China). 2-azidoacetic acid, DBCO-TMARA (Catalogue number 760773), and tyrosinase (from mushroom, product number T3824-25KU) were purchased from Sigma-Aldrich Co. (USA). PNGase F was obtained from New England Biolabs (NEB). Human IgG (Catalogue number ab91102) was purchased from Abcam. In-Gel Tryptic Digestion Kit was purchased from Thermo Fisher Scientific Inc. (USA). Peptide characterization and purification were performed in RP-HPLC (Shimadzu, DGU-20A5, Japan). Peptide analysis was performed in an AutoFlex Speed LRF MALDI-TOF mass spectrometer (Bruker Daltonics, Germany). The gel images were captured by an ENDURO™ GDS Gel Documentation System (USA) or a Bio-Rad ChemiDoc Image System (USA).

Peptide synthesis. All Peptides were synthesized based on manual Fmoc-SPPS chemistry. Briefly, Rink Amide-ChemMatrix® resins (Biotage, Sweden) with a loading capacity of 0.5 mmol/g first swelled in DCM/DMF (50% v/v). For each coupling procedure, five-fold excess of protected amino acid, HBTU, HOBt, and DIEA (with a ratio of 1: 1: 1: 2) in DMF was added to the resin for 35 min with shaking at room temperature (RT). The deprotection reaction of the Fmoc group was performed in 20% piperidine in DMF (v/v) after the resins were washed with DMF for 5 times. For capping the

N-terminus amine, the resin was suspended in a DMF solution containing acetic anhydride (10 equivalents based on resin substitution) and DIEA (10 equivalents based on resin substitution) and shaken at RT for 30 min. Normally, peptides were cleaved from the resin, and the sidechains were deprotected by treatment with TFA/H_2O/TIPS (95/2.5/2.5) for 2 h at RT. Then the resin was filtered and rinsed twice with TFA. The crude peptide was obtained by precipitation by adding cold diethyl ether.

Peptide purification and characterization. Crude peptides were dissolved in 50% acetonitrile (ACN): 50% H_2O containing 0.1% TFA. After being filtered through a 0.2 μm filter, the peptide solution was injected into RP-HPLC (Shimadzu, DGU-20A5, Japan) equipped with a C18 column (Vydac 218TP C18 LC Column 5um, 250 × 4.6mm ID). 0.1% TFA in H_2O (v/v) and 0.1% TFA in ACN (v/v) were used as the mobile phases A and B, respectively. For analytical HPLC analyses, the total flow rate was set to be 1 mL/min, and the B concentration rose from 5 % to 95 % over 13 min following a linear gradient. For the purification of peptides on a larger scale by semi-prep HPLC columns (Vydac 218TP C18 LC Semi-Prep Column 10um, 250x10mm ID), the total flow rate was set to be 3 mL/min (gradient: 0-5 minutes 5% B, 5-30 minutes 5-65% B, 30-33 minutes 65-95% B, 33-36 minutes 95% B). The peptide peaks were collected, lyophilized, and confirmed by MALDI-TOF mass spectrometry analysis (Bruker Daltonics, Germany).

The model reaction of 1a and 2a. Substrates **1a** (0.1 mmol), $NaIO_4$ (0.1 mmol), and **2a** (0.5 mmol) were dissolved in ACN/H_2O (1mL, 1/9, v/v). The reaction system was irradiated by blue light (456 nm, 30 mW/cm^2) for 5 min at RT. The reaction solution was extracted by EtOAc (2 mL) three times. And

then, the product was purified by silica column chromatography (EtOAc/hexane=3/1).

^{1}H NMR (400 MHz, Chloroform-*d*) δ 6.71 (d, *J* = 8.1 Hz, 1H), 6.62 (d, *J* = 8.4 Hz, 1H), 5.84 (dd, *J* = 6.6, 2.2 Hz, 1H), 4.04 – 3.94 (m, 1H), 3.84 – 3.76 (m, 3H), 3.31 (dd, *J* = 16.5, 6.6 Hz, 1H), 3.05 (dd, *J* = 16.5, 2.2Hz, 1H), 2.19 (s, 3H).

^{13}C NMR (100 MHz, Chloroform-*d*) δ 144.13, 138.17, 126.12, 124.93, 122.56, 115.30, 107.18, 70.35, 61.87, 36.45, 18.16.

HRMS (ESI) calcd. for $C_{11}H_{14}NaO_4^+$ 233.0784 [M+Na]$^+$; found 233.0784

Computational details. The reaction mechanisms and the associated free energy barriers of [3+2] photoaddition of 2a to 4MQ were determined with Density Functional Theory (DFT) calculations in Gaussian 16 (ver. C.02).[1] Unrestricted DFT was used for the radical using ωB97XD functional.[2] For closed shell systems, restricted DFT was adapted instead. The 6-31+G(d) basis set was used for all geometry optimizations and frequency calculations along with PCM (water) solvent models.[3] Reaction paths were followed by intrinsic reaction coordinate (IRC) with local quadratic approximation (LQA) to the potential energy.[4,5] To obtain the free energies, thermal correction to Gibbs Free Energy from each frequency calculation was added to the single point energy of each corresponding specie, calculated with the aug-cc-pVTZ basis set in gas phase. The free energies of solvation in water were calculated using M06-2X functional, 6-31G(d) basis set and SMD (water) solvent models at 298.15 K and 1 atm.[6,7]

Cartesian coordinates are listed below:

^{3}R

E(UωB97XD/aug-cc-pVTZ, SMD(water)) = -804.876729063

Solvated Gibbs Free Energy = -504929.38352056 kcal/mol

C	-1.83681016	0.86650699	-0.15662729

C	-0.91565918	2.03997563	-0.21335519
C	0.16073805	2.06611732	0.74250074
C	0.37887603	1.05231556	1.67763148
C	-0.43537959	-0.06989705	1.72386249
C	-1.52954613	-0.16384817	0.79513549
H	0.82008777	2.92929700	0.71180457
H	1.20266537	1.14559157	2.37918985
H	-2.27175108	-0.94476455	0.93388397
C	-0.17794934	-1.18827433	2.68366690
H	-1.10565373	-1.68714751	2.97845319
H	0.46620793	-1.93963687	2.20784237
H	0.33852959	-0.83211254	3.57872330
O	-1.08507293	2.95952133	-1.04130825
O	-2.82932102	0.78853606	-0.92149554
C	-0.58498424	-1.98492224	-0.71326962
H	-1.43646156	-1.80252113	-1.35675262
H	-0.67995789	-2.71777129	0.08121042
C	0.62136706	-1.47376780	-1.05091307
H	0.74648374	-0.80032019	-1.89837185
C	2.95535385	-1.20380424	-0.80722134
H	2.98963836	-1.22765120	-1.90128439
H	3.73237646	-1.86007863	-0.41348691
C	3.14175287	0.21425575	-0.28896333
H	3.10147761	0.21383274	0.80732337
H	2.33825109	0.86155660	-0.66660016
O	4.40602832	0.64423521	-0.76047661
H	4.57360939	1.53666702	-0.43281346
O	1.71635031	-1.75540028	-0.34566392
O	-4.29101552	-1.58418049	-0.94819611
H	-3.82459539	-0.72115969	-0.93538712
H	-4.99321620	-1.50342057	-0.29129457

^{3}TS1

E(UωB97XD/aug-cc-pVTZ, SMD(water)) = -804.877180696

Solvated Gibbs Free Energy = -504927.80325279746 kcal/mol

C	-1.84964372	0.83684246	-0.12132229
C	-0.92913191	2.01056314	-0.12634585
C	0.16600765	1.98571765	0.81090320
C	0.38760744	0.93795515	1.69396117
C	-0.43516069	-0.18694563	1.69855124
C	-1.49551101	-0.27743407	0.72716061
H	0.82217301	2.85218720	0.81562619
H	1.21073394	0.99897722	2.40025192
H	-2.28397491	-1.00477643	0.90537632

C	-0.19075741	-1.32652334	2.63681618
H	-1.12195484	-1.83468391	2.90378040
H	0.46493435	-2.06749601	2.15950899
H	0.30800493	-0.98925410	3.54912428
O	-1.11711142	2.97913690	-0.89431717
O	-2.85964593	0.81023143	-0.86653012
C	-0.64506710	-1.71238559	-0.69345334
H	-1.47506264	-1.56665858	-1.37513339
H	-0.71150255	-2.54170154	0.00380301
C	0.60109571	-1.29044048	-1.09946103
H	0.74016548	-0.60549055	-1.93458749
C	2.94957642	-1.16109858	-0.88780976
H	2.96094376	-1.11338098	-1.98091668
H	3.68781300	-1.88963798	-0.55219124
C	3.22091749	0.20384503	-0.27444215
H	3.18262048	0.13007003	0.81947543
H	2.45686185	0.92196939	-0.60148882
O	4.50772692	0.58596030	-0.72403401
H	4.72906324	1.44375124	-0.34042469
O	1.68195847	-1.66686520	-0.43801065
O	-4.31823563	-1.53797995	-1.03709797
H	-3.84707318	-0.67874301	-0.96231586
H	-4.96088241	-1.53957429	-0.31731682

^{3}IM1

E(UωB97XD/aug-cc-pVTZ, SMD(water)) = -804.885046421

Solvated Gibbs Free Energy = -504933.87984294497 kcal/mol

C	-1.66968679	0.81561770	-0.17267914
C	-0.78985891	2.05148365	0.02197662
C	0.18097598	1.97996311	1.06761001
C	0.36692174	0.83570816	1.85484076
C	-0.32033057	-0.34192754	1.65341681
C	-1.31259812	-0.47201082	0.53453002
H	0.77641107	2.86945541	1.25155336
H	1.09791001	0.88205350	2.65798433
H	-2.25102799	-0.87648307	0.93815232
C	-0.07806093	-1.54469780	2.50568011
H	-1.02093102	-2.03217283	2.77807843
H	0.51963211	-2.28086613	1.95296824
H	0.46541366	-1.28552102	3.41744920
O	-0.96526518	3.04310302	-0.69681252
O	-2.63284489	0.87830753	-0.91948766
C	-0.83774664	-1.53121055	-0.54255860
H	-1.61763269	-1.57047549	-1.30880875

H	-0.81910784	-2.50764236	-0.04734284
C	0.47470781	-1.26494141	-1.17606140
H	0.61892070	-0.48357900	-1.91962087
C	2.82511341	-1.18687924	-0.92915557
H	2.84890551	-1.18172334	-2.02556000
H	3.57180876	-1.89579421	-0.56516800
C	3.10905781	0.20332879	-0.37791719
H	3.08258690	0.17468840	0.71910915
H	2.34318615	0.91106833	-0.72276858
O	4.39313334	0.57507749	-0.85251206
H	4.60765072	1.45269619	-0.51268701
O	1.56594824	-1.66213481	-0.46739463
O	-4.47798931	-1.30306789	-1.05080685
H	-3.88455129	-0.52929796	-1.03420109
H	-5.09400109	-1.16329336	-0.32112269

^{3}TS2

E(UωB97XD/aug-cc-pVTZ, SMD(water)) = -804.857823759

Solvated Gibbs Free Energy = -504914.24010573 kcal/mol

C	-1.23561698	0.44649466	-0.77400278
C	-1.39064843	1.79366939	-0.14041526
C	-1.12600469	1.88647670	1.26777004
C	-0.73262994	0.80049818	2.01217458
C	-0.51574268	-0.47057381	1.42551065
C	-0.78153988	-0.66315601	0.05224315
H	-1.25481031	2.86035090	1.73369134
H	-0.55212164	0.91879353	3.07745489
H	-2.34565987	-1.13795353	0.03705473
C	-0.00937866	-1.59314698	2.28935645
H	-0.66410690	-2.47033720	2.22880620
H	0.98760340	-1.90929232	1.96434334
H	0.04885038	-1.28514675	3.33638086
O	-1.74147599	2.78434099	-0.82674860
O	-1.59268351	0.26459605	-1.96752055
C	-0.25156589	-1.89886484	-0.69127811
H	-0.89960217	-2.13945298	-1.53859157
H	-0.24102913	-2.76961959	-0.02816400
C	1.12040142	-1.63217844	-1.22447963
H	1.24173657	-0.99431824	-2.10030388
C	3.08395683	-0.53336040	-0.48461538
H	3.36630604	-0.47118609	-1.54350014
H	3.96159046	-0.82611649	0.09591075
C	2.54390327	0.80335401	0.00545269
H	2.28479218	0.72625406	1.06930772

H	1.63243378	1.06359188	-0.55053869
O	3.56257003	1.76812995	-0.20817264
H	3.24968430	2.62429327	0.10927787
O	2.11064190	-1.55152246	-0.28935357
O	-3.29877560	-1.47361536	-0.36513819
H	-3.34304458	-1.03829972	-1.24525578
H	-4.02564336	-1.12226885	0.18329633

^{3}IM2

E(UωB97XD/aug-cc-pVTZ, SMD(water)) = -804.906936562

Solvated Gibbs Free Energy = -504945.7033529375 kcal/mol

C	1.70384385	-0.19946484	0.31220560
C	2.79164021	0.34605358	-0.50385411
C	2.66525348	1.71897082	-0.91509989
C	1.57960513	2.45726954	-0.54629013
C	0.53116018	1.90903808	0.25377873
C	0.60061255	0.57932928	0.68433532
H	3.46212823	2.13493167	-1.52373620
H	1.49819777	3.49240587	-0.86608880
H	3.71550931	-3.63222538	-0.65878185
C	-0.63104150	2.78960732	0.62222336
H	-1.58250790	2.30931145	0.37356055
H	-0.64012495	2.99626871	1.69979415
H	-0.57754837	3.74785668	0.09964238
O	3.78365873	-0.36269840	-0.82226387
O	1.75136797	-1.46255292	0.72522341
C	-0.48782172	-0.05855583	1.52508429
H	-1.00001826	0.71969440	2.11071013
H	-0.03629997	-0.74265224	2.24966024
C	-1.47635433	-0.84052315	0.72100636
H	-2.06909097	-1.62336290	1.19285241
C	-3.21429310	-0.76006002	-0.88024769
H	-3.08945790	-1.84934017	-0.91022602
H	-3.27409638	-0.38663807	-1.90532256
C	-4.47288683	-0.38463579	-0.11034585
H	-4.59150736	0.70647547	-0.11384581
H	-4.39047026	-0.72188185	0.93154749
O	-5.56038939	-1.02039845	-0.76483993
H	-6.37922651	-0.77714401	-0.31568957
O	-2.07409888	-0.14863761	-0.29173296
O	3.93094278	-2.95575064	-0.00341094
H	4.20509818	-2.16603826	-0.50845301
H	2.55925798	-1.94953123	0.42379482

^{3}TS3

E(UωB97XD/aug-cc-pVTZ, SMD(water)) = -804.891673002

Solvated Gibbs Free Energy = -504939.0128149075 kcal/mol

C	1.71320699	-0.31195749	0.33433865
C	2.82369020	0.20831433	-0.49037394
C	2.76025269	1.56732619	-0.90730950
C	1.70552441	2.35912625	-0.55193220
C	0.61981091	1.87183949	0.24663333
C	0.62871721	0.56280872	0.68256945
H	3.57592207	1.94692198	-1.51495702
H	1.67902653	3.39346486	-0.88434709
H	3.41283395	-3.41301150	-0.69090831
C	-0.50213648	2.81976223	0.58762755
H	-1.47525832	2.38649373	0.33783774
H	-0.51052371	3.05290487	1.65950873
H	-0.39543940	3.76265673	0.04466901
O	3.83194797	-0.51542506	-0.83541943
O	1.69821527	-1.52802872	0.75531960
C	-0.48855676	-0.02265708	1.52050635
H	-0.98246990	0.78263636	2.08529090
H	-0.06591164	-0.71040410	2.25867882
C	-1.49505104	-0.78755216	0.72240678
H	-2.10004535	-1.55995304	1.19626899
C	-3.23193867	-0.68769196	-0.87819678
H	-3.11797348	-1.77823983	-0.90892062
H	-3.28898031	-0.31351046	-1.90325618
C	-4.48720752	-0.30130508	-0.10816537
H	-4.59744609	0.79067441	-0.11287169
H	-4.40697578	-0.63790472	0.93409911
O	-5.58036012	-0.92919154	-0.76115100
H	-6.39684531	-0.67879754	-0.31166891
O	-2.08586057	-0.08785345	-0.29010388
O	3.62004175	-2.76321473	-0.00212761
H	3.84989259	-1.77206005	-0.47494631
H	2.67044833	-2.33024449	0.40475544

^{3}P

E(UωB97XD/aug-cc-pVTZ, SMD(water)) = -504946.0191642749

Solvated Gibbs Free Energy = -804.906600801 kcal/mol

C	1.70986485	-0.23788832	0.33584579
C	2.77753871	0.33948518	-0.48728137
C	2.69479955	1.66455628	-0.90729851
C	1.59909217	2.42582512	-0.54290590
C	0.53008649	1.91415374	0.25262332

C	0.58089982	0.60858466	0.68754748
H	3.49129880	2.08030911	-1.51600601
H	1.54541262	3.45781498	-0.87872454
H	3.78173043	-3.61053821	-0.69829442
C	-0.62177170	2.82881583	0.59279907
H	-1.58019732	2.36852534	0.33548770
H	-0.64152222	3.05896617	1.66478718
H	-0.54025463	3.77589708	0.05257274
O	3.83633934	-0.38072321	-0.83919733
O	1.78997965	-1.43213656	0.72374586
C	-0.51001914	-0.02415572	1.51972122
H	-1.02925987	0.75664575	2.09612929
H	-0.06018719	-0.70913567	2.24448467
C	-1.49071442	-0.81260712	0.71097182
H	-2.06667841	-1.61204247	1.17566019
C	-3.23713652	-0.74632133	-0.88078681
H	-3.09244310	-1.83275251	-0.92319873
H	-3.30881846	-0.36326378	-1.90155697
C	-4.49892974	-0.40299812	-0.10113945
H	-4.63967466	0.68544183	-0.09422679
H	-4.40411329	-0.74769111	0.93725461
O	-5.57701688	-1.05479014	-0.75553632
H	-6.39807856	-0.83197432	-0.29989615
O	-2.10570661	-0.12009237	-0.29095801
O	3.91806448	-2.97527472	0.01681308
H	3.81147164	-1.31628327	-0.51123229
H	3.03577420	-2.80048406	0.39724133

^{1}P

E(UωB97XD/aug-cc-pVTZ, SMD(water)) = -804.995878945

Solvated Gibbs Free Energy = -504996.9623162899 kcal/mol

C	-1.00112946	0.43161361	0.15524483
C	-1.86041029	1.42548915	-0.30820694
C	-3.15468251	1.01368638	-0.63995670
C	-3.55337489	-0.31710705	-0.50942948
C	-2.68295865	-1.30599095	-0.03353944
C	-1.39530770	-0.89062317	0.29462443
H	-3.85151540	1.76140265	-1.00828845
H	-4.57012565	-0.58839783	-0.78271256
H	1.48677177	3.70367225	0.35158737
C	-3.09958396	-2.74534888	0.12244535
H	-2.48076514	-3.40747840	-0.49504558
H	-2.99210576	-3.07971377	1.16121057
H	-4.14345713	-2.88831869	-0.17176707

O	-1.52149181	2.73080239	-0.47363732
O	0.30992195	0.63296020	0.53379392
C	-0.22056241	-1.66287508	0.83524601
H	-0.01201727	-2.58792183	0.29178479
H	-0.35676526	-1.91154283	1.89357291
C	0.93094538	-0.66850979	0.65121141
H	1.61597139	-0.58964324	1.49920347
C	2.81847803	-0.26358439	-0.74042011
H	2.76180923	0.74241433	-0.30929975
H	2.93281978	-0.16205510	-1.82229331
C	3.99821832	-1.02929544	-0.15833878
H	4.06815885	-2.01302200	-0.63942364
H	3.85865467	-1.18143194	0.92042708
O	5.15914986	-0.25164290	-0.41159756
H	5.93167769	-0.72983616	-0.08605509
O	1.60586645	-0.97900496	-0.52460719
O	0.79017200	3.32594222	0.90447957
H	-0.67498730	2.95627049	-0.02486670
H	0.99913661	2.37842016	0.98123117

Peptide reactions. The starting peptide Fmoc-GGY-OH (10 mM, 5 μL) was incubated with tyrosinase (1.68 μM) at 4 °C in PB Buffer (0.2 M, pH 6.5, 10 μL) for 30 min. 25 μL DI water was added to the reaction system. After incubation, vinyl ether (100 mM, 5μL) was added and irradiated by 456 nm blue light (Kessil Lamp, 25% intensity, 30 mW/cm^2) for various periods. The reaction rate constants of conjugation of Fmoc-GGY-OH oxidized form (3d*) and VE were calculated based on HPLC analysis results. The relative amounts of Fmoc-GGY-OH oxidized form (3d*) and product were monitored by absorbance at 254 nm and calculated by integrating peak areas at 254 nm. Percent conversion (x) was calculated by the appearance of the product. The purified product (5 mM, 10 μL) was used as the standard to calculate the reaction yield. Pseudo-first order rate constant k_1 was measured by plotting ln[1/(1-x)] versus time and analysis by linear regression.[8,9]

1) Tyrosinase oxidation. The maximum absorption wavelength of quinone is 405 nm. Fmoc-Tyr-OH (10 mM, 20 μL) was incubated with tyrosinase (1.68 μM) in PB Buffer (pH 6.5, 160 μL) at 4℃. And

the OD value at 405 nm was detected every 5 min.

2) Peptide reaction with VE molecules. Peptide (10 mM) was incubated with tyrosinase (1.68 μM) in PB buffer (0.2 M, pH 6.5) at 4 °C for 30 min, then VE (100 mM) was added and irradiated at 456 nm light (Kessil lamp, 25% intensity, 30 mW/cm^2) for 5 min. The reaction yield was determined by the integrated peak area of the product in HPLC chromatograms, and the new peak was subjected to MALDI-TOF MS analysis to confirm it as the conjugation product.

Plasmid construction. *E. coli* TOP10 strain was used for cloning and plasmid propagation and grown in selective Luria-Bertani medium or Luria Bertani plates with 1.5 wt% agar. Antibiotics were added for selection at the following concentrations: 100 μg/mL ampicillin or 50 μg/mL kanamycin. The DNA sequences of the plasmid constructs containing PCR fragments were confirmed by sequencing. The primers were ordered from BGI. Plasmids were constructed using standard restriction enzyme cloning techniques.

Expression and purification of proteins. BL21 (DE3) cells were grown at 37 ºC until cultures reached an OD600 value between 0.6-0.8, upon which protein expression was induced with a final concentration of 0.1 g/L IPTG. Cells were then shaken overnight at 37 ºC and pelleted. Then cell pellets were resuspended in 15 mL of an equilibration buffer (20 mM sodium phosphate, 2 M NaCl, 20 mM imidazole at pH = 7.4) and then lysed via sonication for 30 min at 60% amplitude. The cell lysate was centrifuged at 14,000 rpm for 30 min; then, the supernatant was decanted before loading onto a HisTrap Crude column. After binding, the bound protein was washed with 4 portions of two resin bed volumes of wash buffer (20 mM sodium phosphate, 300 mM NaCl, 25 mM imidazole at pH 7.4) and

subsequently eluted with four resin bed volumes of elution buffer (20 mM sodium phosphate, 300 mM NaCl, 250 mM imidazole at pH = 7.4). The purified protein was then spun-concentrated into 20 mM phosphate buffer at pH 7.2 using 10 kDa MWCO or 30 kDa MWCO filters. Purified protein samples were flash-frozen and stored at -80 ºC until use.

Reactions of the recombinant proteins and product analysis. Protein (10 μM) was incubated with tyrosinase (100 nM) and small molecules (1000 μM) in PB buffer (0.2 M, pH 6.5) at 4 °C under 456 nm irradiation (Kessil lamp, 20 mW/cm^2) for 60 min. 12% acrylamide gels were prepared according to BIO-RAD bulletin 6201 protocol. 12 μL 0.5 mg/mL protein solution was diluted with 3 μL 5 × sample buffer, including 5% 2-mercaptoethanol, and heated to 95 °C for 10 minutes. After loading the samples, the gel was run using a BIO-RAD Mini-PROTEAN Tetra Vertical Electrophoresis Cell at 150 volts until completion. Fluorescently labeled proteins were analyzed prior to staining using a BioRad ChemiDoc™ system. Subsequently, the gel was stained using a staining solution containing 1 g/L Coomassie Brilliant Blue R-250 in 5:4:1 (v/v/v) methanol: water: acetic acid for 30 min. The gel was subsequently destained using 5:4:1 (v/v/v) methanol: water: acetic acid for 60 min, after which it was further destained overnight using deionized water.

Western blotting experiments. Add 5 μg of the modified protein into SDS Sample Buffer (5 X) and boil samples for 10 min before loading. Run the samples on a pre-cast SDS polyacrylamide gel at 170V (constant voltage) for 40 to 60 minutes until the dye reaches the bottom of the gel. Remove the gel and soak it in 1L of protein transfer buffer for 15 minutes. Cut the nitrocellulose membrane (ISC BioExpress, F-3139-3) to a similar size to the transfer area of the gel. Assemble the electroblotting

cassette and place the electrodes in the blotting unit, then transfer in the transfer buffer at 100 V for 1 hour at a constant current (not to exceed 0.4 A). Following the transfer, remove the membrane from the blotting cassette and mark the orientation of the gel with a pencil. Rinse briefly with PBS. Wash the membrane with TBST (10mM Tris-HCl, pH 8.0, 150 mM NaCl, 0.05% Tween 20) once for 5 min at room temperature. Block non-specific binding on the membrane with freshly prepared 5% nonfat dried milk for 1 hour on a shaking platform at room temperature. Incubate the membrane with primary biotin antibody (BK-1/39, SC-53179 from Santa Cruz) diluted in TBST and 5% nonfat dried milk at 4° C overnight. Wash three times for 5 min each with TBST. Incubate with Anti-mouse-IgG, HRP-conjugated secondary antibody (7076S from Cell Signaling Technology), in TBST-5% nonfat dried milk for 1 hour at room temperature. Wash three times again for 5 minutes each with TBST. For detection, use Western Blot Luminol Reagent and prepare according to instructions. Lay the membrane on a plastic surface with the protein side up. Add the mixed detection solution to the membrane. Incubate for 1 minute. Remove the excess solution and cover the membrane with transparent plastic. Use Bio-Rad ChemiDoc Image System (USA) to detect the signal.

Antibody deglycosylation. Trastuzumab (Tras) (200 µg, 5 mg/mL in PBS pH 7.4) was incubated with PNGase F (2.0 µL, 250 units) at 37 °C. After overnight incubation the antibody was dialyzed (3 times to PBS pH 5.5) and concentrated to 2.0 mg/mL.

Procedure for antibody conjugation. Deglycosylated trastuzumab (20 µL, 2.0 mg/mL, 40 µg in PBS pH 5.5, final concentration 5 µM) was diluted with 20 µL PB buffer pH 6.5 and incubated with VE-N_3 (5 µL, 5 mM in DMSO, final concentration 500 µM) and mushroom tyrosinase (5.0 µL, 1.7 mg/mL

in phosphate buffer pH 6.0, final concentration 2 μM) and irradiated under 456 nm light (20 mW/cm^2) for 8 h at 4 °C. After completion, the product was concentrated, and small molecules were removed by 30 kDa MWCO filters. After that, DBCO-PEG$_4$-TAMRA (5 μL, 2 mM in DMSO) was added and incubated at room temperature for 1.5 h.

Cell imaging experiment. 1×10^5 SKOV3 or MDA-MB-231 cells were seeded in 35-mm glass-bottom tissue culture dishes. When reaching 80% confluency, cells were incubated with Trastuzumab-(VE)-TAMRA (30 μg/mL) for 45 minutes and then washed with PBS three times. After that, cells were incubated with DAPI (10 μg/mL) at 37 °C for 10 min. After washing with PBS, cells were imaged immediately under a Leica confocal microscope with corresponding filters.

Cell-based anti-proliferation assays. SKOV3 and MDA-MB-231 cell lines were cultured in DMEM medium supplemented with 10% FBS and 1.0% Penicillin-Streptomycin. Cells were seeded (100 μL) at a density of 5×10^3 cells/well (SKOV3) or 2×10^3 cells/well (MDA-MB-231) in 96-well cell culture microplates. Plates were incubated for 24 h at 37°C, 5% CO_2. Subsequently, the cells were aspirated, and respective wells on the microplate were directly subjected to solutions of ADCs /antibodies in medium (5% PBS; 100 μL) starting at 200 nM final concentration to 0.0001nM final concentration. Plates were incubated for 72 h at 37°C, 5% CO_2. Subsequently, the cells were aspirated, and CCK8 reagents (10 μL in 100 μL DMEM) were added, followed by incubation for 4 h at 37°C, 5% CO_2. Metabolic conversion of WST-8 to WST-8 formazan was quantified by the absorbance signal at 450 nm on a Thermofisher microplate reader. Raw data were normalized to 0% viability (cells treated with 5 μM MMAE in medium with 5% PBS; 100 μL) and 100% viability (cells treated with medium with

5% PBS; 100 μL). Means and standard errors of the mean (SEM) were calculated from three biological replicates containing triplicate datasets (N = 3, n = 3) and plotted against ADC/antibody concentration. IC_{50} values were calculated using nonlinear regression (log inhibitor vs response).

Antibody-cell conjugation. The cell used for conjugation (THP1 cell or NK 92 cell) was spun down at 300 g for 2 min. Then remove supernatant and resuspend cells in ~ 1 mL of DPBS. Repeat the centrifugation step and remove the supernatant to wash the cells. Repeat until a total of three washes have been completed. On the final wash, resuspend about 1×10^6 cells in 1 mL of DPBS. Cells were incubated with 20 μM nbHER2-BPA or Tras-BPA for 10 min at 37°C. The tube containing cells and nbHER2-BPA or Tras-BPA was put on ice and irradiated by 365 nm light 20 mW/cm^2 for 20 min. After the irradiation, add 1 mL of DPBS to each tube and spin the tube at ~300 g for 2 min. Wash the cells until a total of two washes have been performed and move on to any downstream applications.

Flow cytometry analysis. nbHER2-BPA or Tras-BPA were labeled with AF488-NHS ester (10 μM) at room temperature for 60 min. And the extra AF488-NHS ester was excluded by desalting columns. Then THP1 cells were incubated with 20 μM nbHER2-BPA/AF488 or Tras-BPA/AF488 and irradiated under 365 nm light as described above. The group without both nbHER2-BPA/AF488 and light irradiation and the group with nbHER2-BPA/AF488 but without light irradiation were set as control groups. After the reaction, the cells were washed with DPBS 3 times and analyzed by flow cytometry in AF 488 channel by counting 1×10^5 cells.

Fluorescent staining. SKOV3 and THP1cells were stained with Mito Trakcer Red (Invitrogen) and

Dio (Dioctadecyloxacarbocyanine perchlorate, Sigma-Aldrich), respectively, following the manufacturer's protocol. THP1 cells were conjugated with nbHER2-BPA as described above. Non-labeled THP1 cells were used as negative controls. After washing with PBS, the THP1 cells were resuspended with RPMI media with 10 % FBS, then mixed with SKOV3 cells in the same media and incubated at 37 ℃ and 5 % CO_2. After 8 hours, cells were gently washed with PBS 3 times and imaged on a confocal microscope (SP8, Leica).

Cytotoxicity assay. Target cell cytotoxicity was evaluated as previously described. Adherent tumor cells (SKOV3, MDAMB231) were seeded at 2.5×10^4 cells/well in 96-well flat-bottom plates in the complete RPMI medium and incubated overnight at 37°C in a humidified 5% CO_2 atmosphere. Target cells were incubated with nbHER2-NK-92 conjugate or Tras-NK-92 for 48 h in NK 92 culture medium containing 0.2% interleukin-2 (IL-2) (Purchasing from PROCELL) at 37℃, 5% CO_2 in a 2:1 Effector cells/Target cells (E/T) ratio (1×10^5 cells/well). Cellular cytotoxicity was measured via the release of lactate dehydrogenase (LDH) from dead target cells by using CyQUANT™ LDH Cytotoxicity Assay (Invitrogen™) according to the manufacturer's instructions. Spontaneous LDH release was assessed using target and effector cells without antibodies. Maximal target cell lysis was achieved by incubation of target cells with lysis buffer. The percentage of cytotoxicity towards target cells was calculated based on the following formula:

$$\%\text{Cytotoxicity} = \frac{\textit{Experiment Value} - \textit{Effector Cells Spontaneous Control} - \textit{Target Cells Spontaneous Control}}{\textit{Target Cell Maximum Control} - \textit{Target Cells Spontaneous Control}} \times 100$$

Detailed synthetic procedures of chemical molecules

2-azido-N-(3-(vinyloxy)propyl)acetamide (VE-N_3)

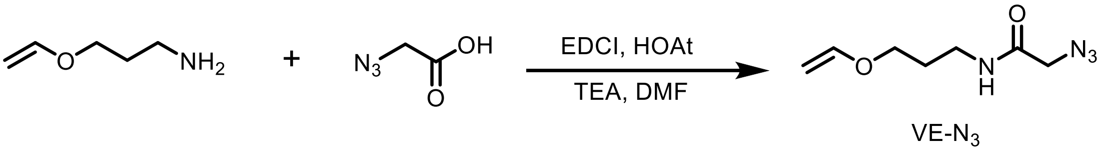

In a round bottle flask, 3-(Vinyloxy)-1-propylamine(0.5 mmol), 2-azidoacetic acid (1.2 eq), EDCI (1.2 eq), HOAt (1.05 eq) and TEA (1.5 eq) were dissolved in DMF (5 mL) and stirred on an ice bath for one hour. After that, the reaction solution was stirred at room temperature for 12 hours. Then 30 mL water was added to the flask, and ethyl acetate (10 mL × 3) was used to extract the product three times. The organic layer was washed with NaCl-saturated solution (10 mL) twice and dried with anhydrous Na_2SO_4. Then the organic layer was concentrated and purified by column chromatography (PE/EA=2/1) to get colorless oil 70 mg (yield 76%) (Figures S43 in the Supporting Information).

^{1}H NMR (400 MHz, DMSO-d_6) δ 8.16 (s, 1H), 6.50 (dd, J = 14.3, 6.8 Hz, 1H), 4.18 (dd, J = 14.3, 1.7 Hz, 1H), 3.98 (dd, J = 6.8, 1.7 Hz, 1H), 3.81 (s, 2H), 3.68 (t, J = 6.3 Hz, 2H), 3.17 (q, J = 6.6 Hz, 2H), 1.75 (p, J = 6.6 Hz, 2H).

^{13}C NMR (100 MHz, DMSO-d_6) δ 167.25, 151.88, 86.81, 65.19, 50.79, 35.69, 28.57. HRMS (ESI) calcd. for $C_7H_{12}N_4O_2$ 185.10329 $[M+H]^+$; found 185.10330.

N-(3-(vinyloxy)propyl)hex-5-ynamide(VE-Alkyne)

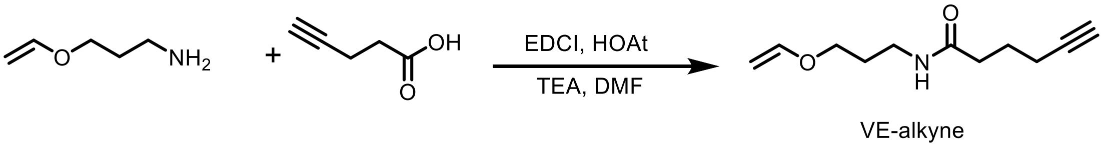

In a round bottle flask, 3-(Vinyloxy)-1-propylamine(0.5 mmol), pent-4-ynoic acid (1.2 eq), EDCI (1.2 eq), HOAt (1.05 eq) and TEA (1.5 eq) were dissolved in DMF(5 mL) on an ice bath for one hour. After that, the reaction solution was stirred at room temperature for 12 hours. Then 30 mL water was

added to the flask, and ethyl acetate (10 mL ×3) was used to extract the product three times. The organic layer was washed with NaCl-saturated solution (10 mL) twice and dried with anhydrous Na_2SO_4. Then the organic layer was concentrated and purified by column chromatography (PE/EA=1/1) to get a solid of 45 mg (yield 46%) (Figure S44 in the Supporting Information).

^{1}H NMR (400 MHz, DMSO-d_6) δ 7.94 (t, J = 5.6 Hz, 1H), 6.50 (dd, J = 14.3, 6.8 Hz, 1H), 4.17 (dd, J = 14.3, 1.7 Hz, 1H), 3.97 (dd, J = 6.9, 1.7 Hz, 1H), 3.68 (t, J = 6.4 Hz, 2H), 3.12 (q, J = 6.5 Hz, 2H), 2.76 (t, J = 2.6 Hz, 1H), 2.35 (dd, J = 6.7, 2.6 Hz, 2H), 2.25 (t, J = 7.2 Hz, 2H), 1.72 (p, J = 6.7 Hz, 2H).

^{13}C NMR (100 MHz, DMSO-d_6) δ 170.23, 151.91, 86.77, 71.32, 65.28, 39.53, 35.47, 34.22, 28.75, 14.30.

HRMS (ESI) calcd. for $C_{10}H_{15}NO_2$ 204.0994[M+Na]$^+$; found 204.0995

5-((4S)-2-oxohexahydro-1H-thieno[3,4-d]imidazol-4-yl)-N (3(vinyloxy)propyl) pentanamide (VE-Biotin)

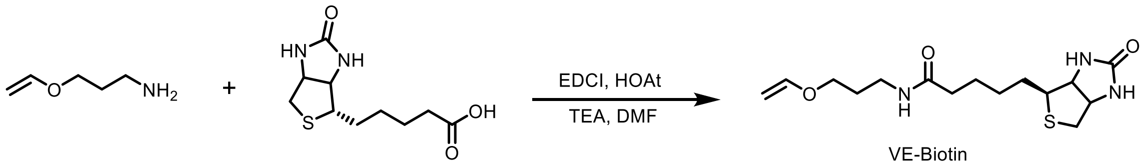

The compound was synthesized according to the above procedures with 23 mg white solid obtained (68% yield) in 0.1mmol scale starting materials (Figure S45 in the Supporting Information).

^{1}H NMR (400 MHz, Methanol-d_4) δ 6.47 (dd, J = 14.4, 6.8 Hz, 1H), 4.51 – 4.46 (m, 1H), 4.30 (dd, J = 7.9, 4.5 Hz, 1H), 4.18 (dd, J = 14.4, 1.9 Hz, 1H), 3.96 (dd, J = 6.8, 1.9 Hz, 1H), 3.71 (t, J = 6.2 Hz, 2H), 3.29 – 3.17 (m, 4H), 2.92 (dd, J = 12.8, 5.0 Hz, 1H), 2.20 (t, J = 7.4 Hz, 2H), 1.84 (p, J = 6.6 Hz, 2H), 1.72 (dtd, J = 12.9, 6.9, 6.0, 2.5 Hz, 2H), 1.63 (dt, J = 8.8, 6.2 Hz, 2H), 1.43 (dd, J = 8.7, 6.5 Hz,

2H).

^{13}C NMR (100 MHz, Methanol-d_4) δ 174.72, 164.72, 151.57, 85.49, 65.09, 61.97, 60.22, 55.61, 39.66, 36.03, 35.40, 28.58, 28.40, 25.50, 24.90.

HRMS (ESI) calcd. for $C_{15}H_{25}N_3O_3S$ 328.16894 $[M+H]^+$; found 328.16844

3',6'-dihydroxy-3-oxo-N-(3-(vinyloxy)propyl)-3H-spiro[isobenzofuran-1,9'-xanthene]-5-carboxamide (VE-FAM)

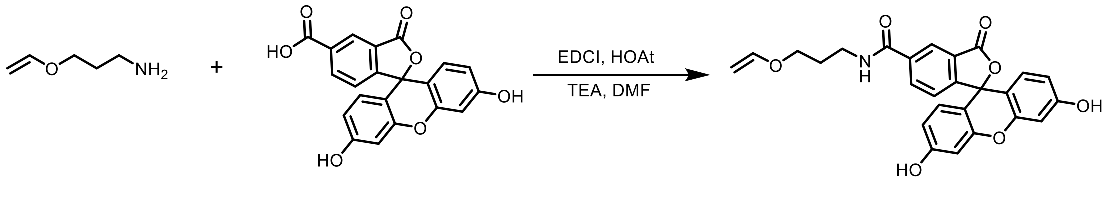

The compound was synthesized according to the above procedures with 22 mg yellow solid obtained (48% yield) in 0.1mmol scale starting materials. (Figures S46 in the Supporting Information)

^{1}H NMR (400 MHz, Methanol-d_4) δ 8.13 – 8.03 (m, 2H), 7.60 (d, J = 1.4 Hz, 1H), 6.68 (d, J = 2.3 Hz, 2H), 6.59 (d, J = 8.7 Hz, 2H), 6.53 (dd, J = 8.7, 2.4 Hz, 2H), 6.36 (dd, J = 14.3, 6.8 Hz, 1H), 4.14 – 4.04 (m, 2H), 3.88 (dd, J = 6.8, 1.9 Hz, 1H), 3.68 (t, J = 6.0 Hz, 2H), 3.39 (t, J = 7.0 Hz, 2H), 1.85 (q, J = 6.4 Hz, 2H).

^{13}C NMR (100 MHz, Methanol-d_4) δ 169.20, 166.80, 163.46, 152.63, 151.46, 140.93, 128.94, 128.91, 124.79, 122.62, 112.37, 109.50, 102.21, 85.55, 65.38, 60.16, 37.12, 35.59, 30.28, 28.34, 19.50, 13.09.

HRMS (ESI) calcd. for $C_{26}H_{21}NNaO_7$ 482.1210$[M+Na]^+$; found 482.1213

DBCO-Vc-MMAE

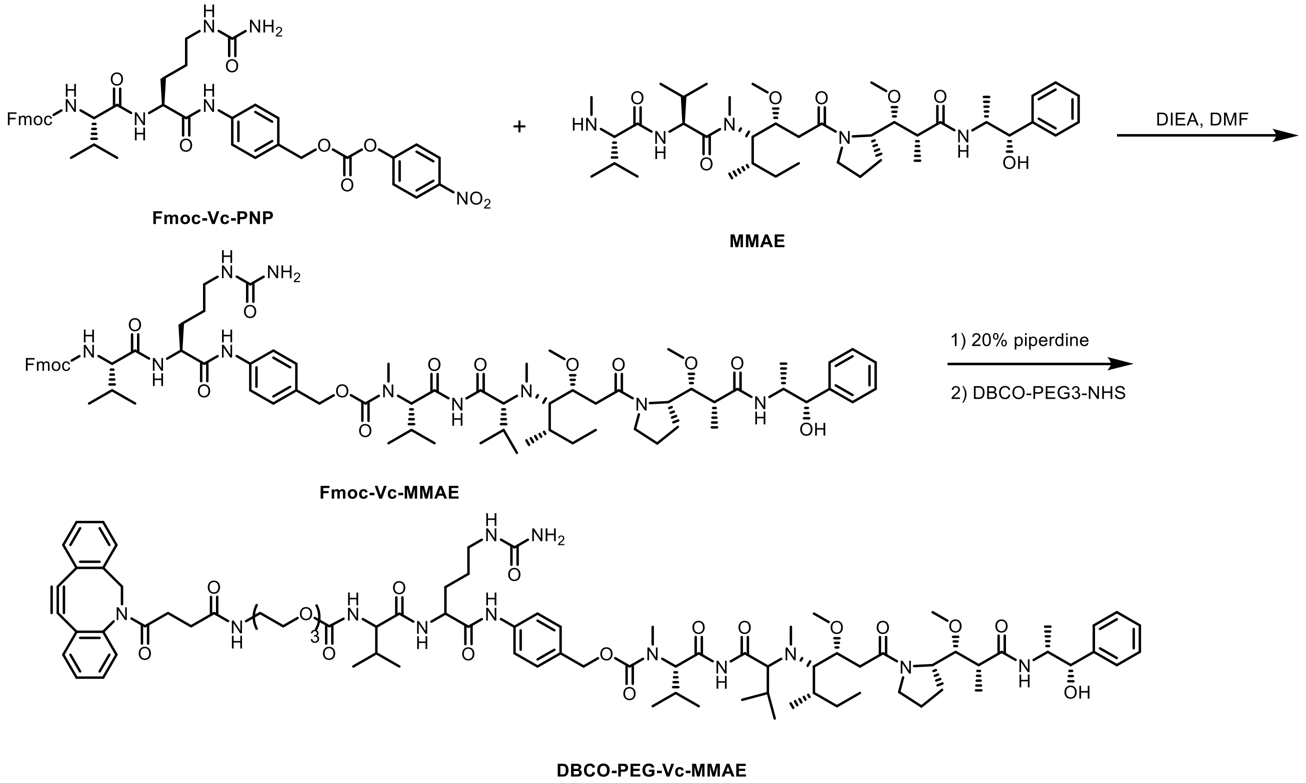

To a solution of the PNP carbonate compound Fmoc-Vc-PNP (6.9 mg, 9.0 µmol) in DMF (200 µL) was added solid MMAE (5.8 mg, 8.1 µmol) followed by the addition of Et_3N (2.4 µL, 18 µmol). After 16 h, the mixture was purified via reversed-phase (C18) HPLC chromatography. Fmoc-Vc-MMAE was obtained as a white solid (7.3 mg, 5.4 µmol, 67%). HR-ESI calculated for $C_{73}H_{104}N_{10}NaO_{14}^+$ $[M+Na]^+$ 1367.7626, found 1367.7618. Then the Fmoc-Vc-MMAE was added to the 20% piperidine in DMF and stirred for 30 min. The NH_2-Vc-MMAE was purified via HPLC. After that, NH_2-Vc-MMAE (6.0 mg, 5.4 µmol) was reacted with DBCO-PEG$_3$-NHS(6.0 µmol) in DMF with DIEA(2.0 eq) overnight. The final product DBCO-PEG$_3$-Vc-MMAE was obtained by HPLC purification in white solid (3.0 mg,1.9 µmol, 35%). HR-ESI calculated for $C_{80}H_{112}N_{12}NaO_{16}^+$ $[M+Na]^+$ 1635.9049, found 1635.9064 (Figure S41 in the Supporting Information).

DBCO-BPA

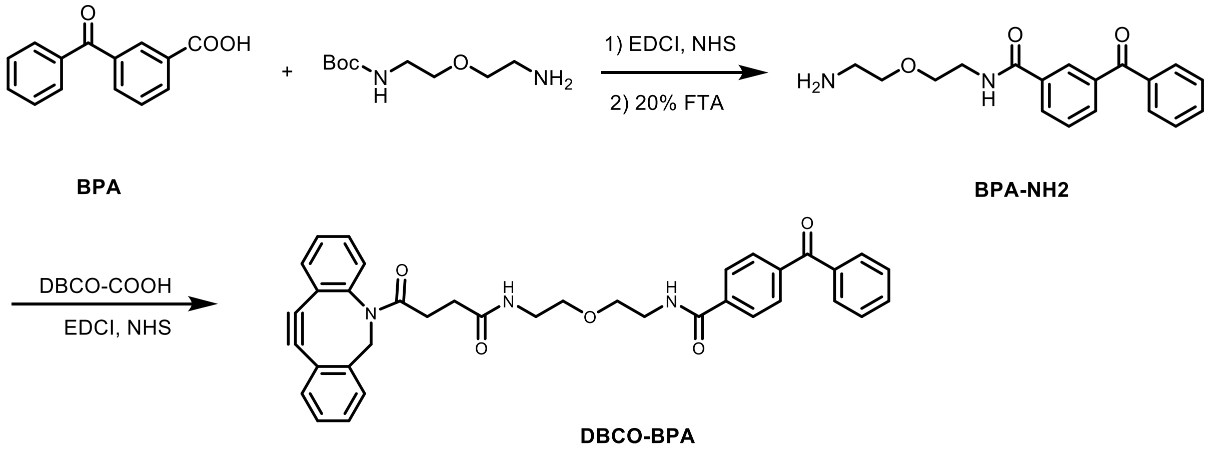

In a round bottom flask, benzophenone-3-carboxylic acid (BPA) (1.0 eq), tert-butyl (2-(2-aminoethoxy)ethyl)carbamate (1.2 eq), EDCI (1.2 eq), NHS (1.2 eq) and TEA (1.5 eq) were dissolved in DMF (10 mL) and stirred at room temperature overnight. The product was extracted by ethyl acetate (10 mL ×3). Then the organic layer was concentrated and dissolved in 20% TFA in DCM. The solution was stirred at room temperature for 2 hours. The solution was concentrated and dissolved in DMF with DBCO-COOH(1.2 eq), EDCI (1.2 eq), NHS (1.2 eq) and TEA (1.5 eq). The reaction was stirred overnight. The final product DBCO-BPA was obtained by HPLC purification in light yellow solid. HR-ESI calculated for $C_{37}H_{33}N_3NaO_5^+$ $[M+Na]^+$ 622.2312, found 622.2315.

Proteins used in this study. The primers of MBP-GGY and GFP-GGY were designed according to previous work.[10] And the plasmids of nbHER2-GGY and nbHER2-GGF were directly purchased from BGI company (Shenzhen, China).

a) MBP-GGY

Forward：5’- AATTCGGAGGAGGAGGTTCTGGCGGCTATTAAC-3’

Reverse：5'- TCGAGTTAATAGCCGCCAGAACCTCCTCCTCCG -3'

Protein sequence:

HHHHHHMKIKTGARILALSALTTMMFSASALAKIEEGKLVIWINGDKGYNGLAEVGKKFEK

DTGIKVTVEHPDKLEEKFPQVAATGDGPDIIFWAHDRFGGYAQSGLLAEITPDKAFQDKLYPF

TWDAVRYNGKLIAYPIAVEALSLIYNKDLLPNPPKTWEEIPALDKELKAKGKSALMFNLQEP

YFTWPLIAADGGYAFKYENGKYDIKDVGVDNAGAKAGLTFLVDLIKNKHMNADTDYSIAE

AAFNKGETAMTINGPWAWSNIDTSKVNYGVTVLPTFKGQPSKPFVGVLSAGINAASPNKEL

AKEFLENYLLTDEGLEAVNKDKPLGAVALKSYEEELVKDPRIAATMENAQKGEIMPNIPQMS

AFWYAVRTAVINAASGRQTVDEALKDAQTGGGGSGGY*

b) GFP-GGY

Forward：5’- AATTCGGAGGAGGAGGTTCTGGCGGCTATTAAC-3’

Reverse：5'- TCGAGTTAATAGCCGCCAGAACCTCCTCCTCCG -3'

protein sequences:

MHHHHHHMASMTGGQQMGRGSMVSKGEELFTGVVPILVELDGDVNGHKFSVSGEGEGDA

TYGKLTLKFICTTGKLPVPWPTLVTTLTYGVQCFSRYPDHMKQHDFFKSAMPEGYVQERTIF

FKDDGNYKTRAEVKFEGDTLVNRIELKGIDFKEDGNILGHKLEYNYNSHNVYIMADKQKNG

IKVNFKIRHNIEDGSVQLADHYQQNTPIGDGPVLLPDNHYLSTQSALSKDPNEKRDHMVLLE

FVTAAGITLGMDELYKELRRQASGGGGSGGY*

c) nbHER2-GGY

protein sequences:

MGSSHHHHHHSSGLVPRGSHMQVQLQESGGGSVQAGGSLKLTCAASGYIFNSCGMGWYRQ

SPGRERELVSRISGDGDTWHKESVKGRFTISQDNVKKTLYLQMNSLKPEDTAVYFCAVCYNL

ETYWGQGTQVTVSSGGGGSGGY*

d) nbHER2-GGF

protein sequences:

MGSSHHHHHHSSGLVPRGSHMQVQLQESGGGSVQAGGSLKLTCAASGYIFNSCGMGWYRQ

SPGRERELVSRISGDGDTWHKESVKGRFTISQDNVKKTLYLQMNSLKPEDTAVYFCAVCYNL

ETYWGQGTQVTVSSGGGGSGGF*

Statistical analysis: All data are presented as mean ± standard deviation (SD). Statistical analysis was conducted with GraphPad Prism 8.0 (GraphPad Software Inc, USA). At least three independent samples were used for statistical analysis in each experiment. Student's t-test was used to assess statistical significance between two groups. Differences were considered statistically significant at $P < 0.05$.

A)

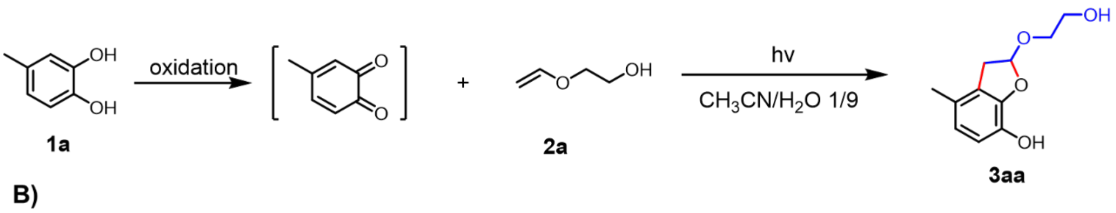
OH
OH
1a
oxidation
O
O
+
OH
O
2a
hv
CH_3CN/H_2O 1/9
OH
O
O
OH
3aa

B)

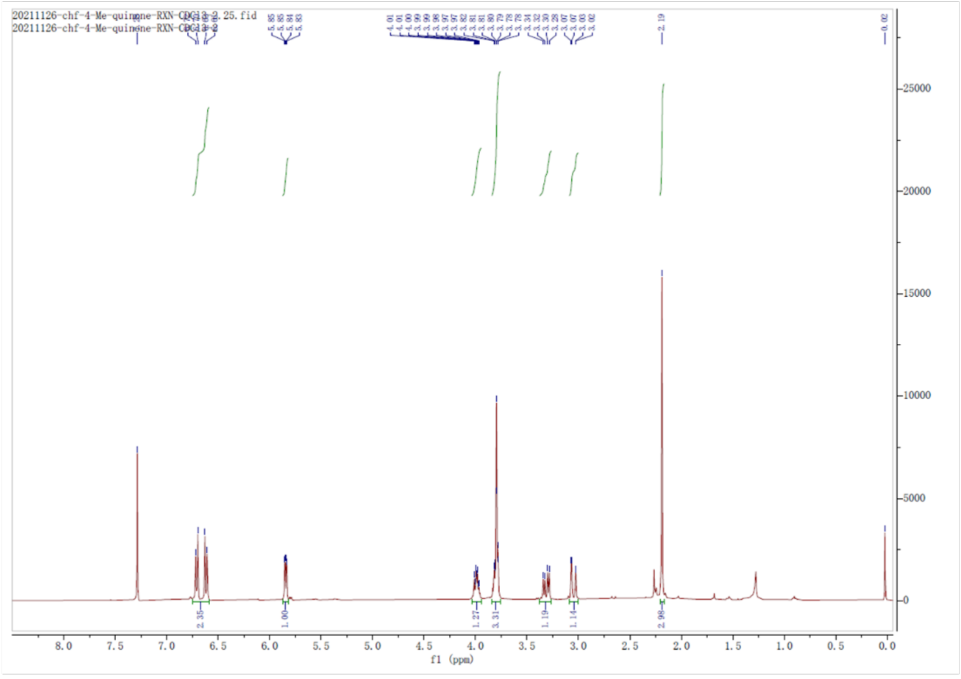
2.19
0.02
2.35
1.00
1.27
3.31
1.19
1.14
2.98
f1 (ppm)

C)

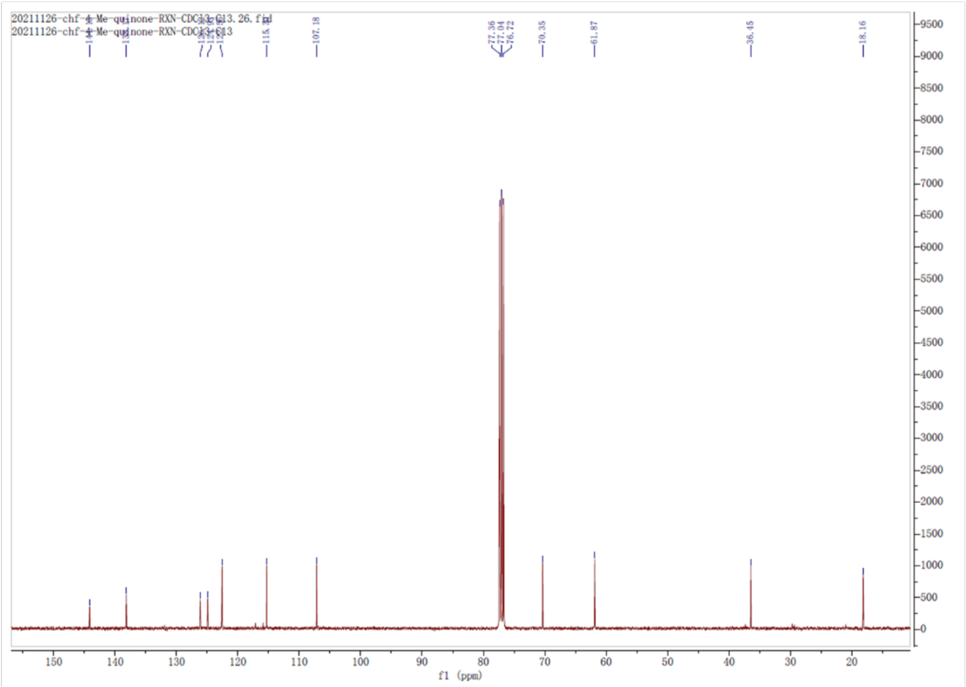
107.18
77.36
77.04
76.72
70.35
61.87
36.45
18.16
f1 (ppm)

D)

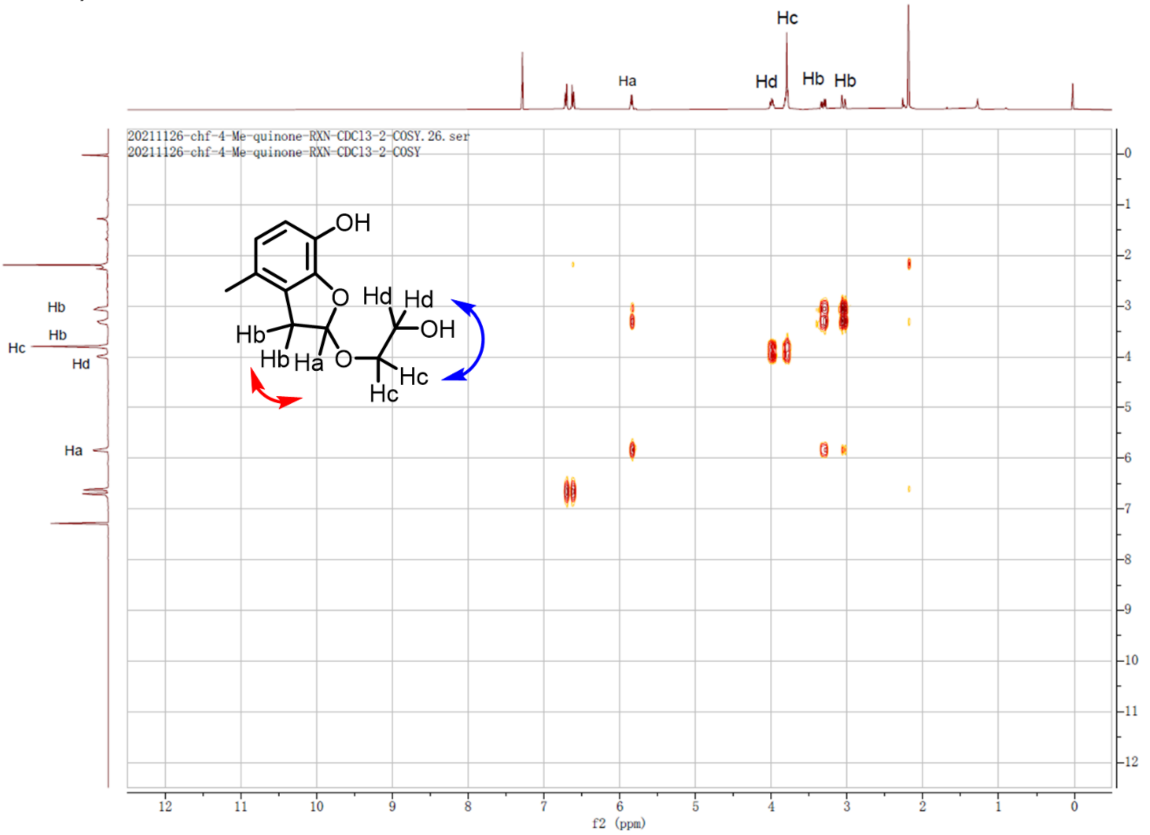

Figure S1. The model reaction between **1a** and **2a**. **A)** Reaction scheme. **B)** H[1]-NMR spectrum of product **3aa**. **C)** C[13]-NMR spectrum of product **3aa**. **D)** H[1]-H[1] COSY spectrum of product **3aa**.

A)

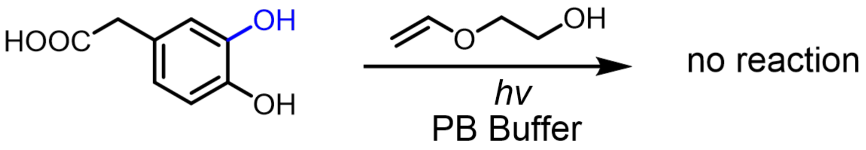

B)

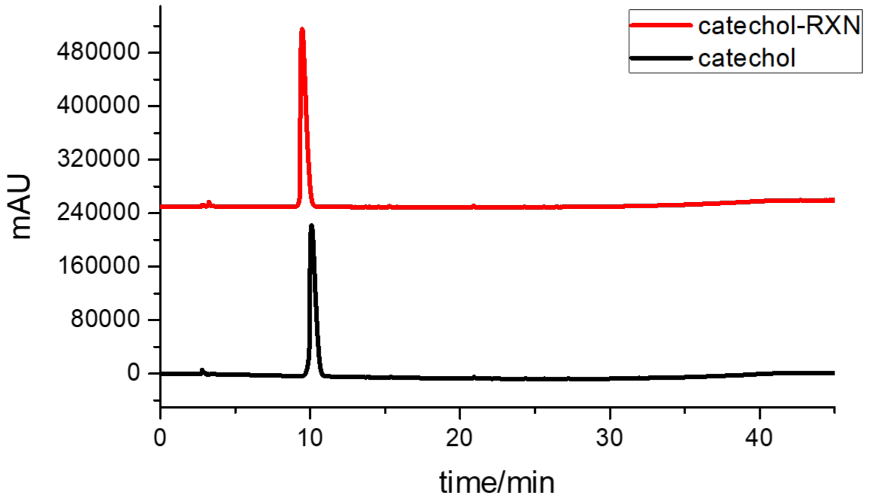

Figure S2. Catechol and vinyl ether did not react. **A)** Reaction scheme. **B)** HPLC chromatogram showing no reaction between catechol and vinyl ether.

A)

B)

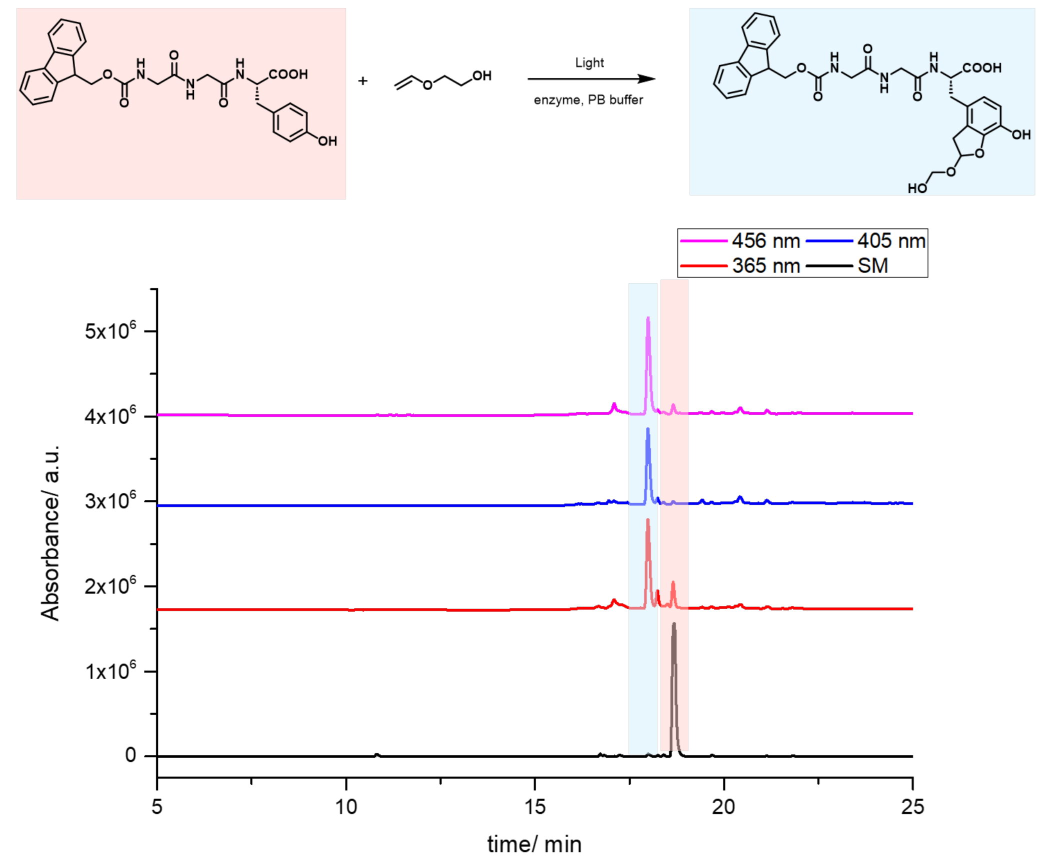

Figure S3. Photoaddition reactions under different wavelengths of light. Briefly, peptide Fmoc-GGY-OH (10 mM, 5 µL) was incubated with tyrosinase (1.68 µM) at 4 °C in PB Buffer (0.2 M, pH 6.5, 10 µL) for 30 min. 25 µL DI water was added to the reaction system. After incubation, vinyl ether (VE, 100 mM, 5µL) was added and irradiated by different wavelengths (30 mW/cm^2). The reaction results were analyzed by HPLC. **A)** Reaction scheme of photoaddition reaction between Fmoc-GGY-OH and VE. **B)** HPLC spectra of peptide reactions under different wavelengths.

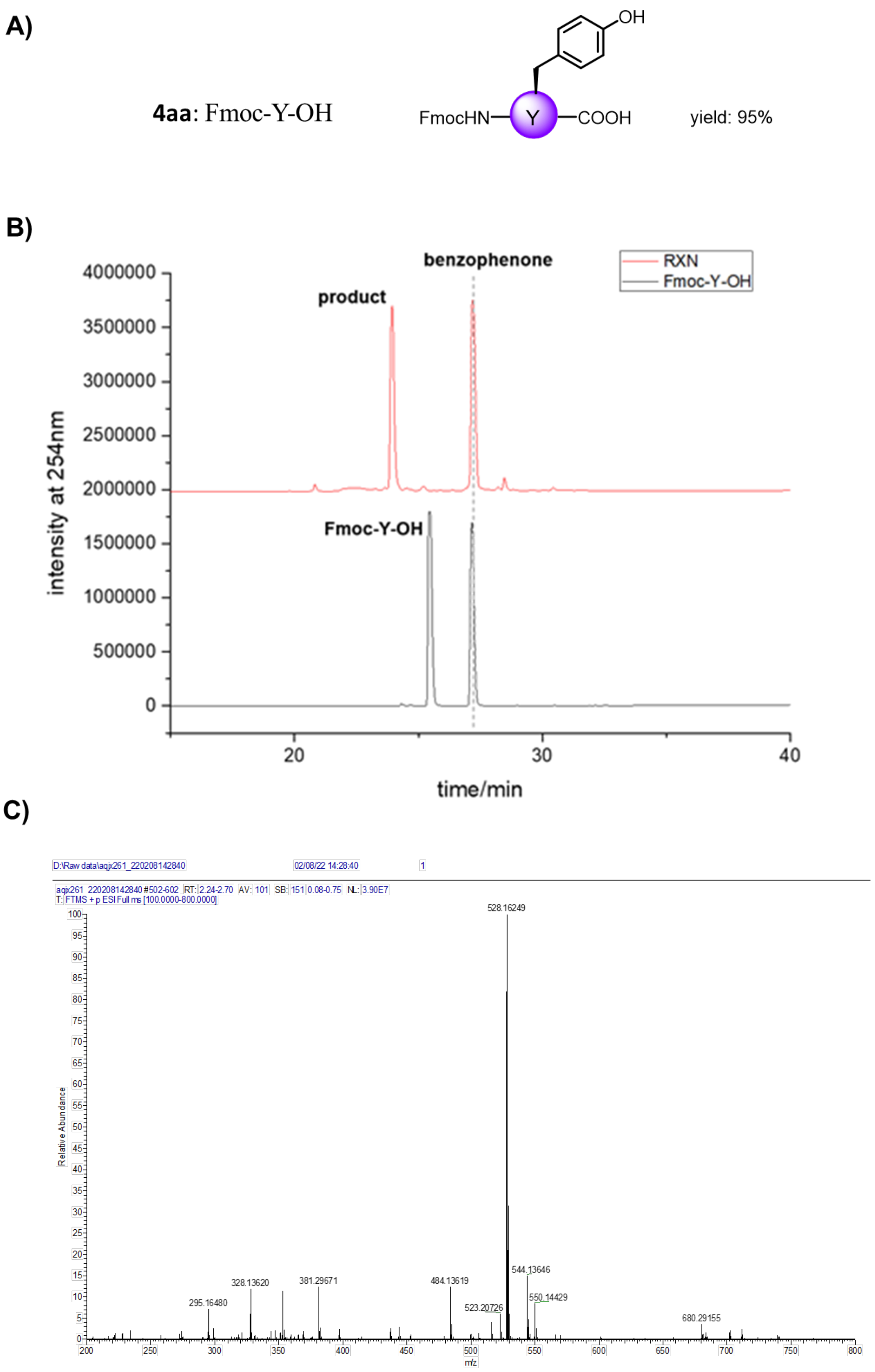

Figure S4. Reaction between Fmoc-Y-OH and vinyl ether. **A)** Structure and reaction yield of Fmoc-Y-OH. **B)** HPLC chromatogram. **C)** HR-ESI MS of the product peak. $[M+Na]^+$calcd for $C_{28}H_{27}NNaO_8^+$ 528.1625 found: 528.1628. Benzophenone was used as an internal standard.

A)

4ab: Fmoc-FY-OH

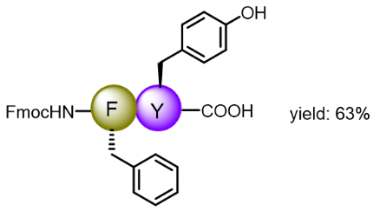

B)

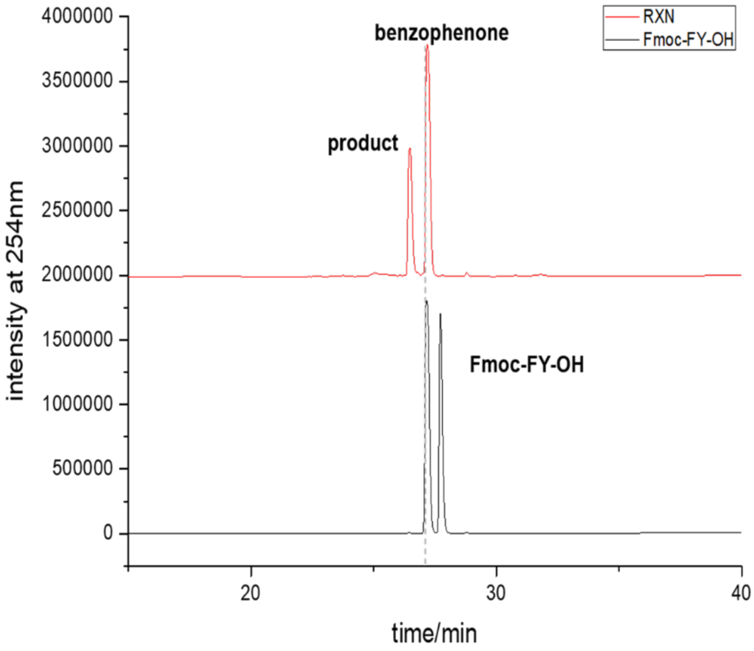

C)

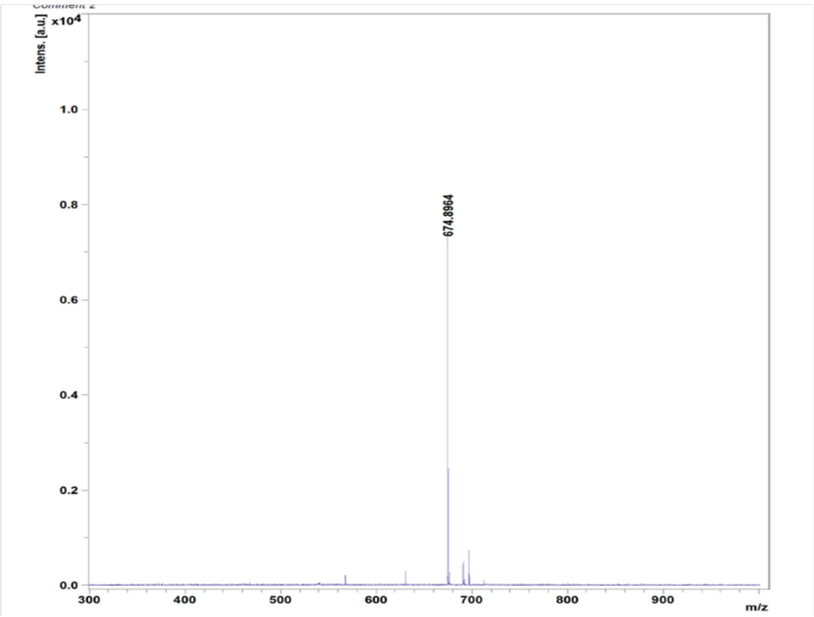

Figure S5. Reaction between Fmoc-FY-OH and vinyl ether. **A)** Structure and reaction yield of Fmoc-FY-OH. **B)** HPLC chromatogram. **C)** HR-ESI MS of the product peak. $[M+Na]^+$ calcd for $C_{37}H_{36}N_2NaO_9^+$ 675.2313 found:674.8964.

A)

4ac: Fmoc-GY-OH

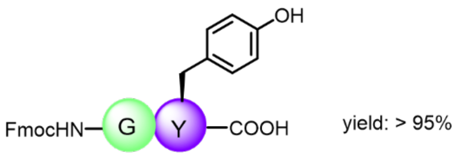

B)

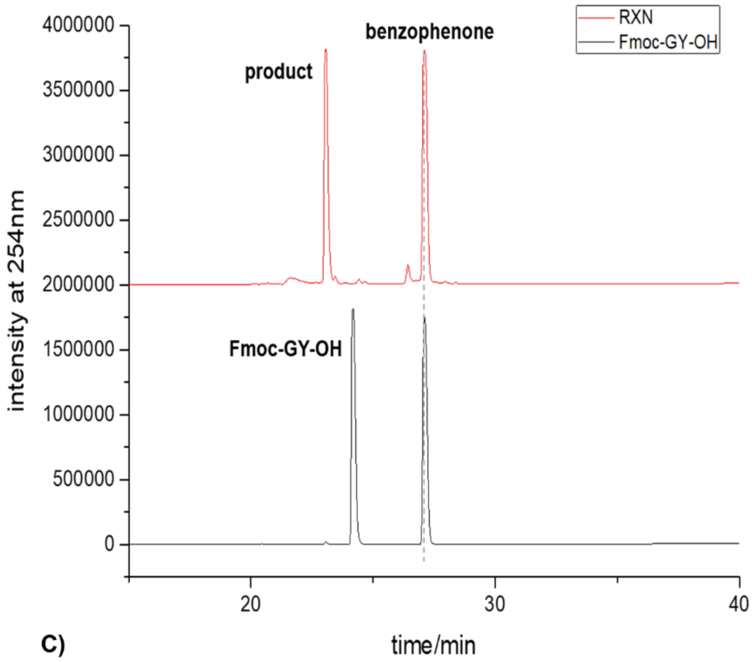

C)

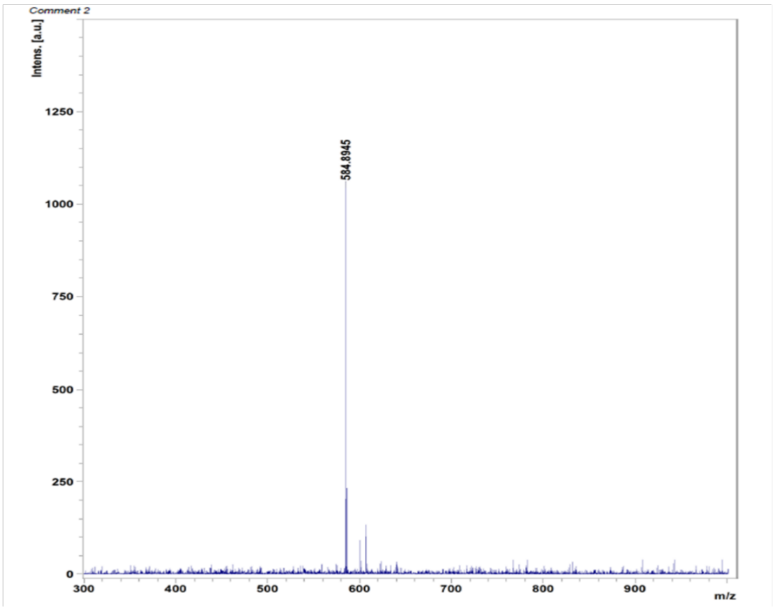

Figure S6. Reaction between Fmoc-GY-OH and vinyl ether. **A)** Structure and reaction yield of Fmoc-GY-OH. **B)** HPLC chromatogram. **C)** HR-ESI MS of the product peak.$[M+Na]^+$ calcd for $C_{30}H_{30}N_2NaO_9^+$ 585.1844 found:584.8945.

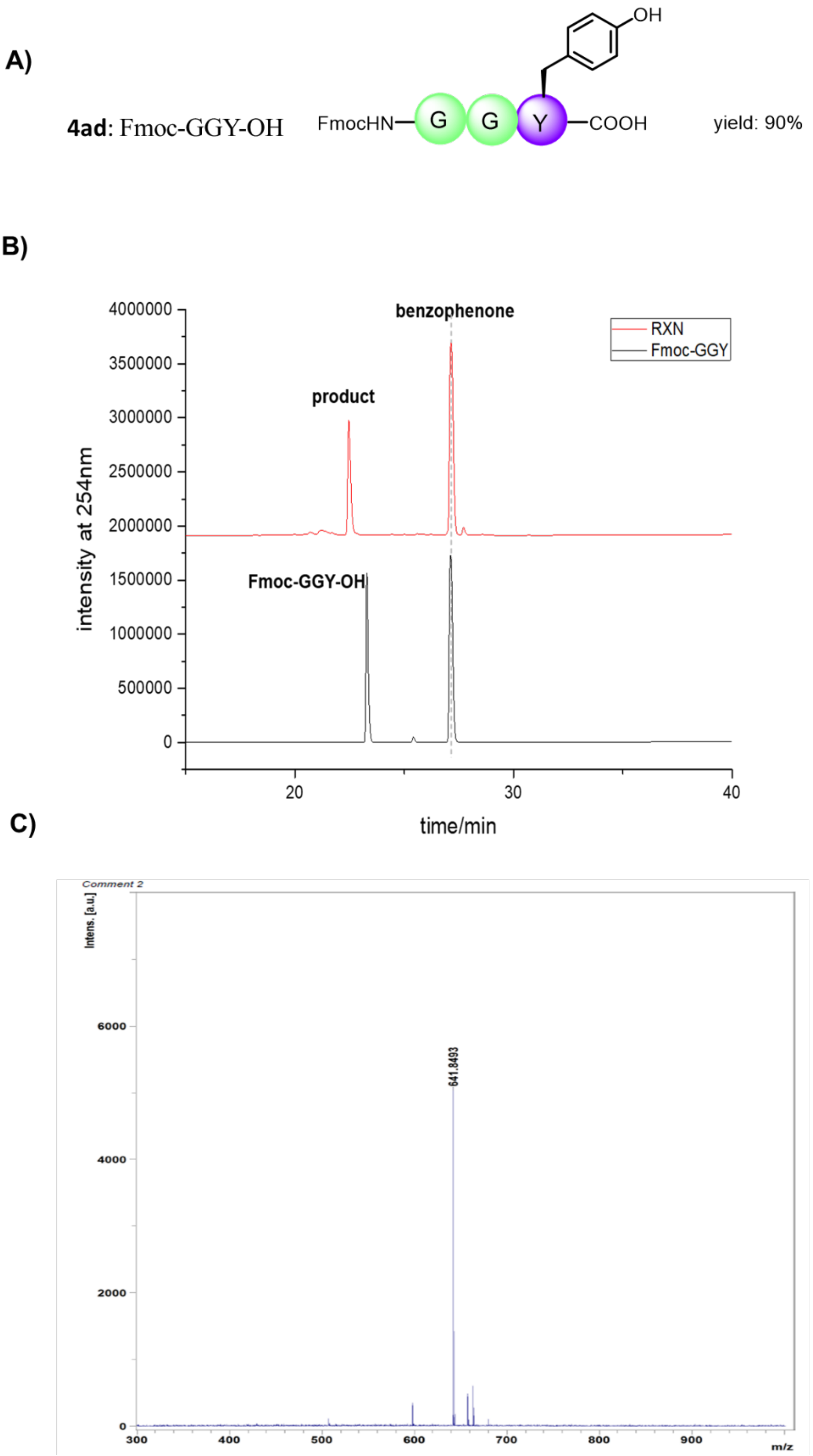

Figure S7. Reaction between Fmoc-GGY-OH and vinyl ether. **A)** Structure and reaction yield of Fmoc-GGY-OH. **B)** HPLC chromatogram. **C)** HR-ESI MS of the product peak. $[M+Na]^+$ calcd for $C_{32}H_{33}N_3NaO_{10}^+$ 642.2058 found:641.8493.

A)

4ae: Fmoc-CY-OH

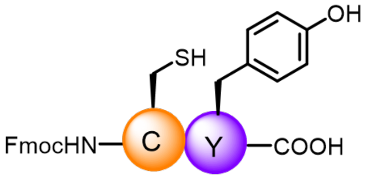

no reaction

B)

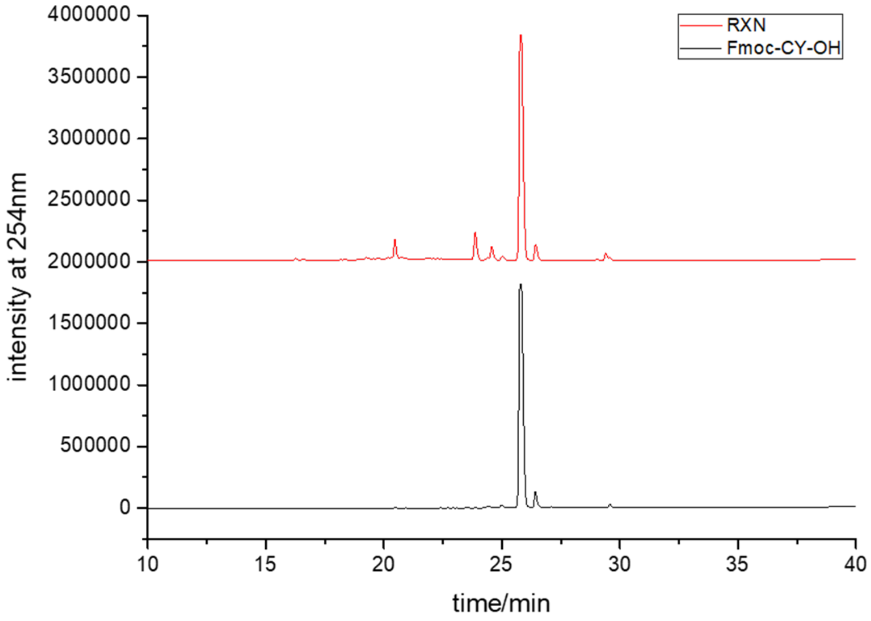

Figure S8. Fmoc-CY-OH did not react with vinyl ether. **A)** Structure and reaction yield of Fmoc-CY-OH. **B)** HPLC chromatogram.

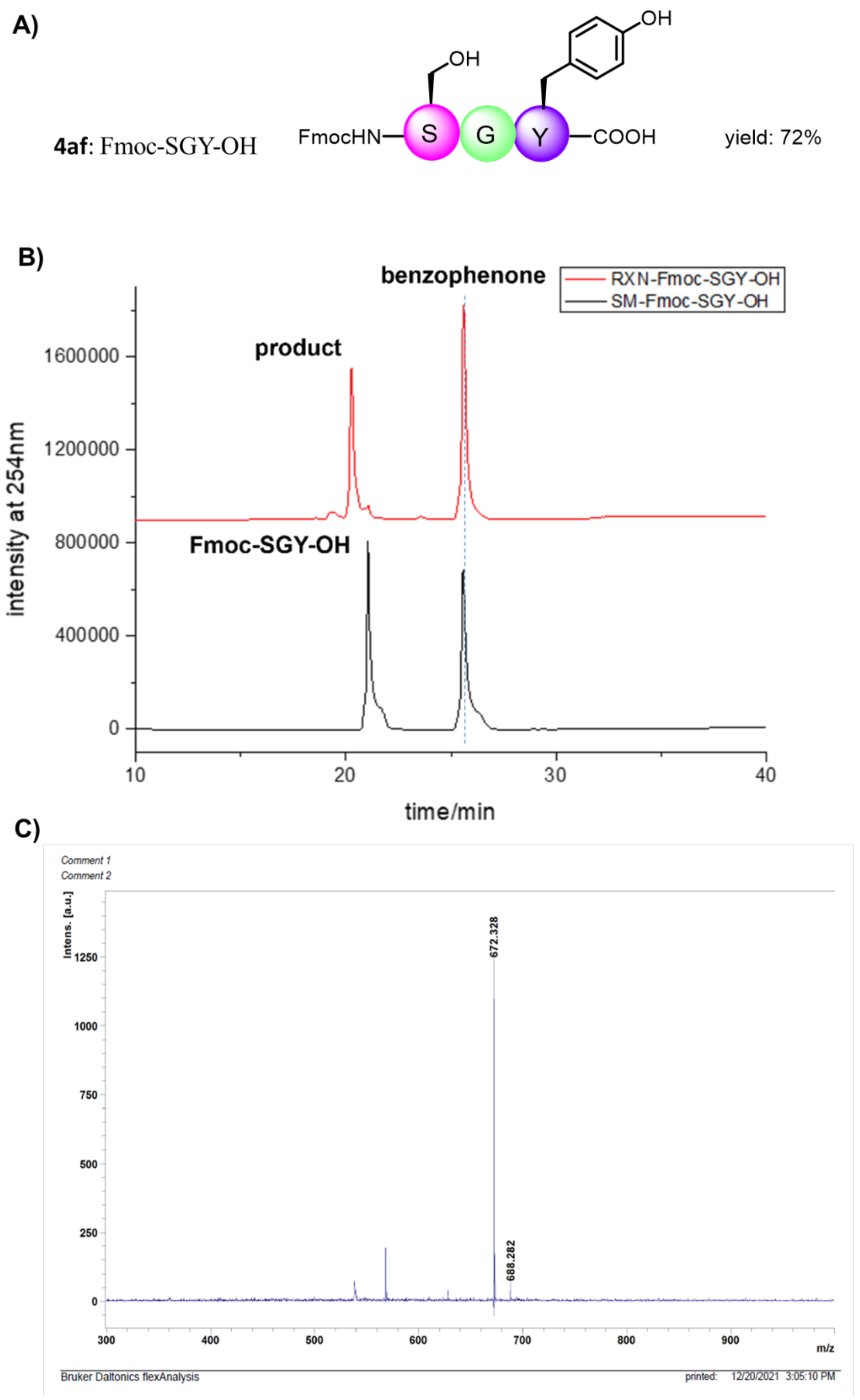

Figure S9. Reaction between Fmoc-SGY-OH and vinyl ether. **A)** Structure and reaction yield of Fmoc-SGY-OH. **B)** HPLC chromatogram. **C)** HR-ESI MS of the product peak. $[M+Na]^+$ calcd for $C_{33}H_{35}N_3NaO_{11}^+$ 672.216 found: 672.328.

A)

4ag: Fmoc-KGY-OH

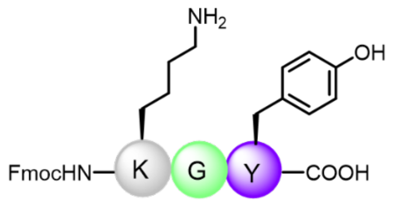

yield: 47%

B)

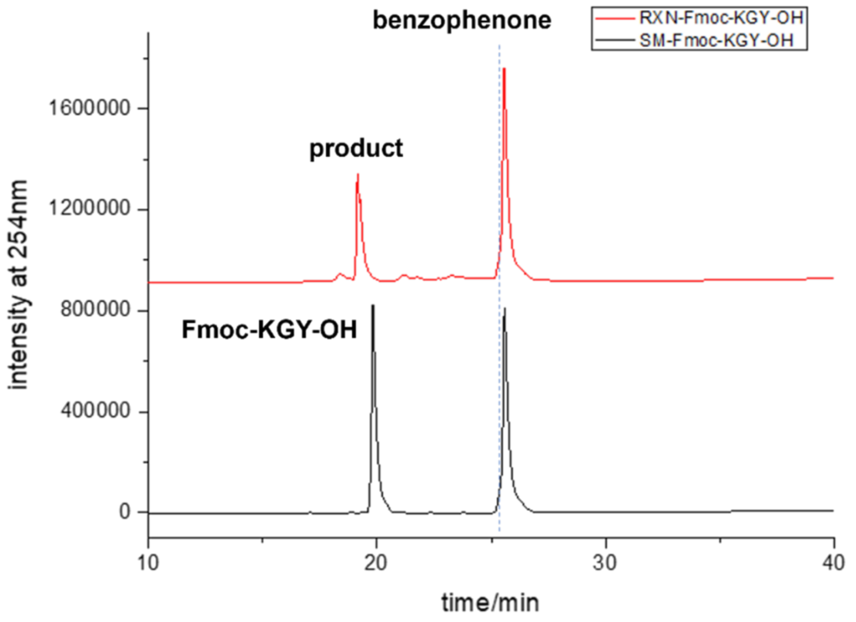

C)

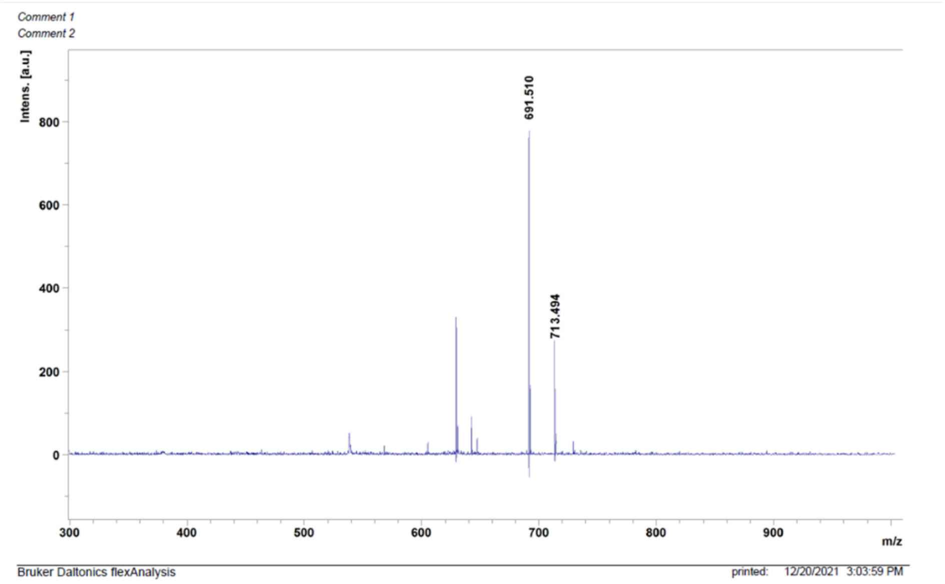

Figure S10. Reaction between Fmoc-KGY-OH and vinyl ether. **A)** Structure and reaction yield of Fmoc-KGY-OH. **B)** HPLC chromatogram. **C)** HR-ESI MS of the product peak. $[M+Na]^+$ calcd for $C_{36}H_{42}N_4NaO_{10}^+$ 713.280 found: 713.494

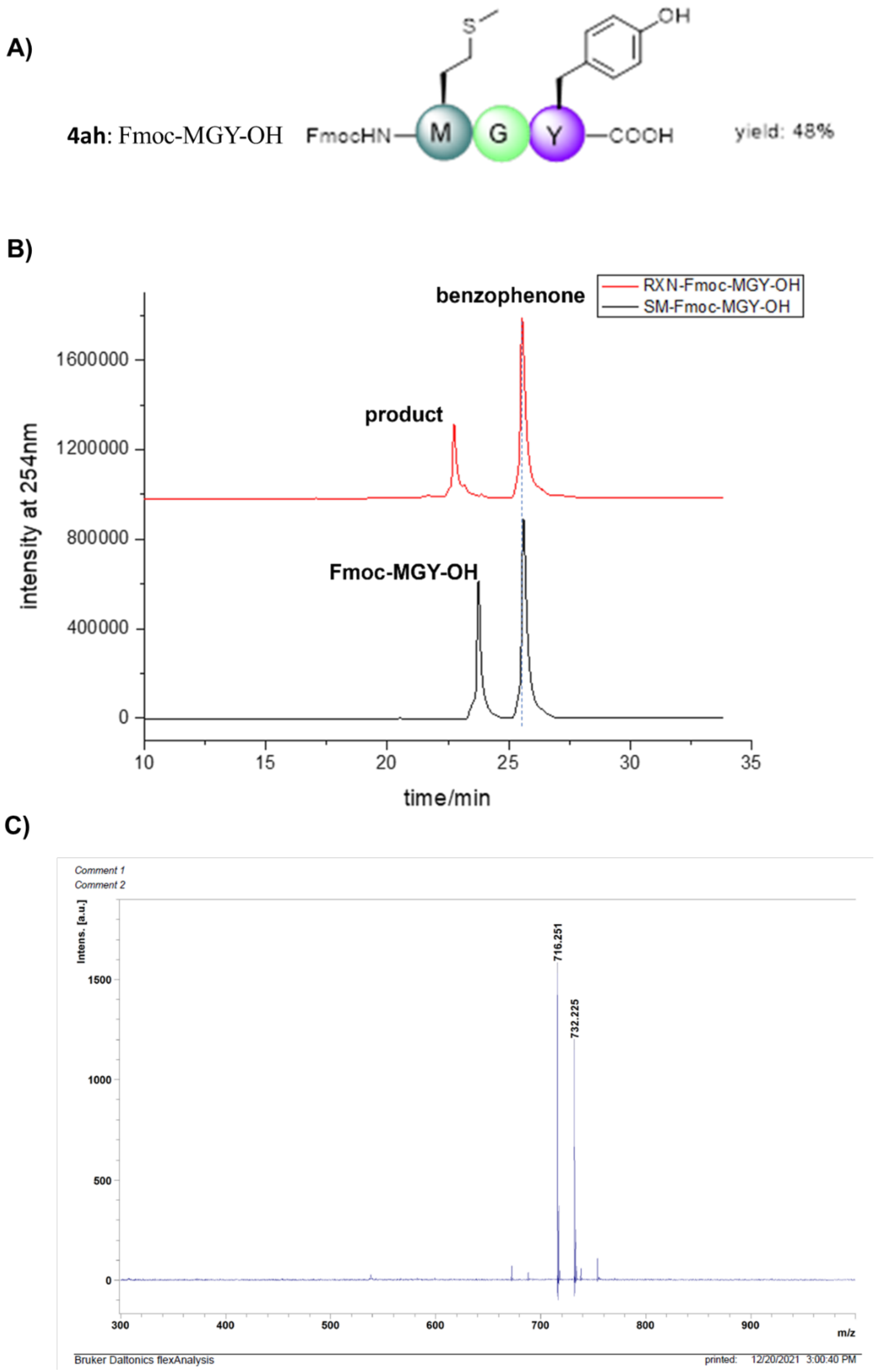

Figure S11. Reaction between Fmoc-MGY-OH and vinyl ether. **A)** Structure and reaction yield of Fmoc-MGY-OH. **B)** HPLC chromatogram. **C)** HR-ESI MS of the product peak. $[M+Na]^+$calcd for $C_{35}H_{39}N_3NaO_{10}S^+$ 716.225 found: 716.251

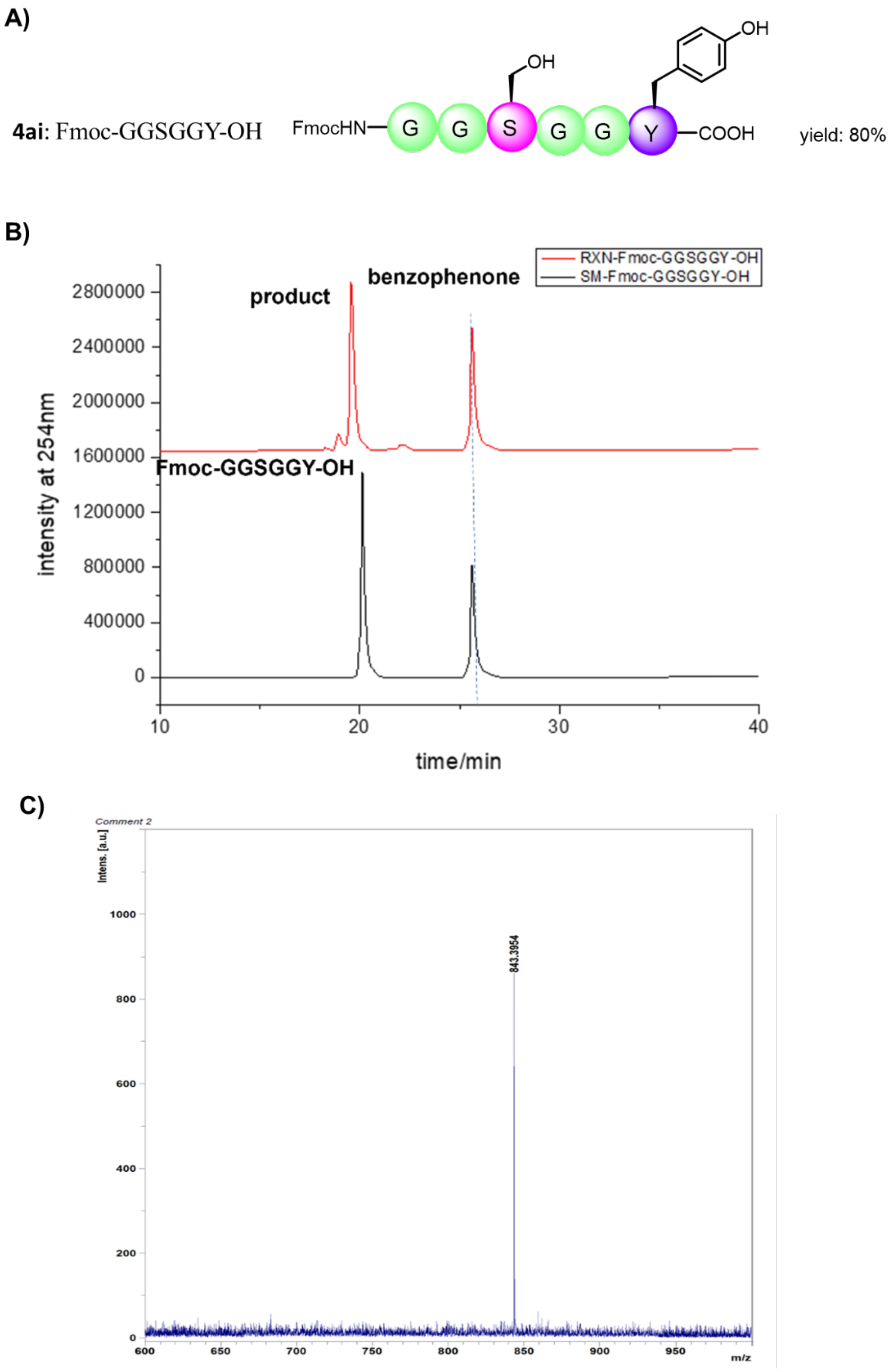

Figure S12. Reaction between Fmoc-GGSGGY-OH and vinyl ether. **A)** Structure and reaction yield of Fmoc-GGSGGY-OH. **B)** HPLC chromatogram. **C)** HR-ESI MS of the product peak. $[M+Na]^+$calcd for $C_{39}H_{44}N_6NaO_{14}^+$ 843.2808found: 843.3954.

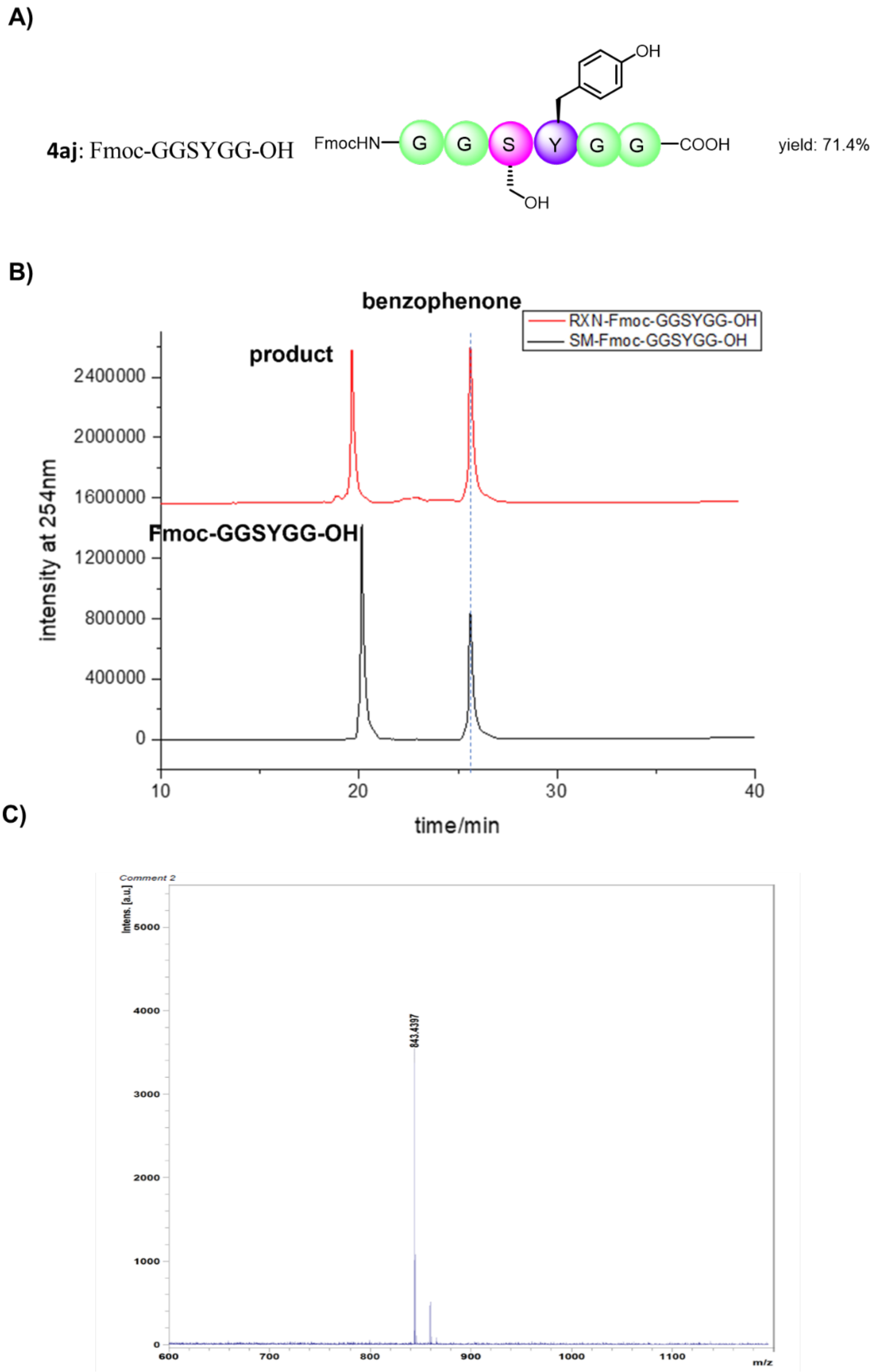

Figure S13. Reaction between Fmoc-GGSYGG-OH and vinyl ether. **A)** Structure and reaction yield of Fmoc-GGSYGG-OH. **B)** HPLC chromatogram. **C)** HR-ESI MS of the product peak. $[M+Na]^+$calcd for $C_{39}H_{44}N_6NaO_{14}^+$ 843.2808found: 843.4397.

A)

4ak: Fmoc-YGGSGG-OH

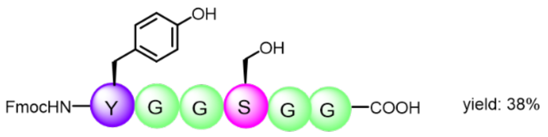

B)

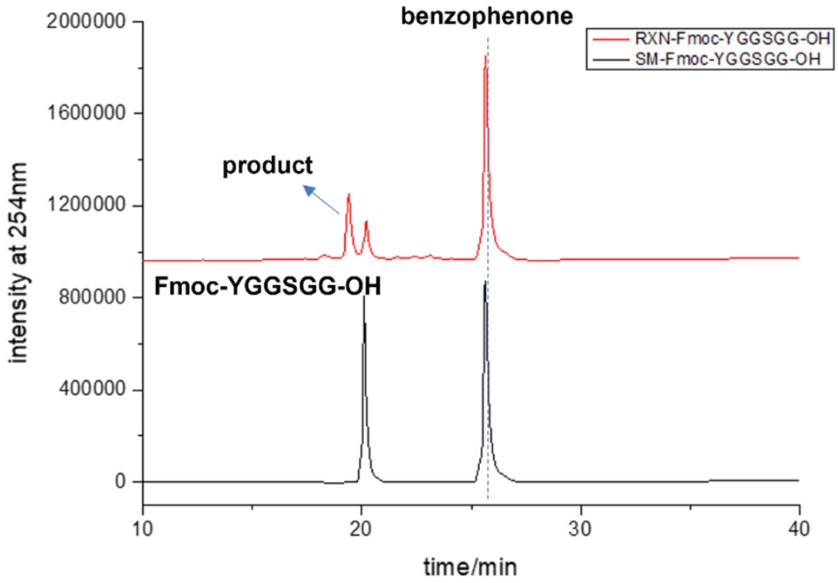

C)

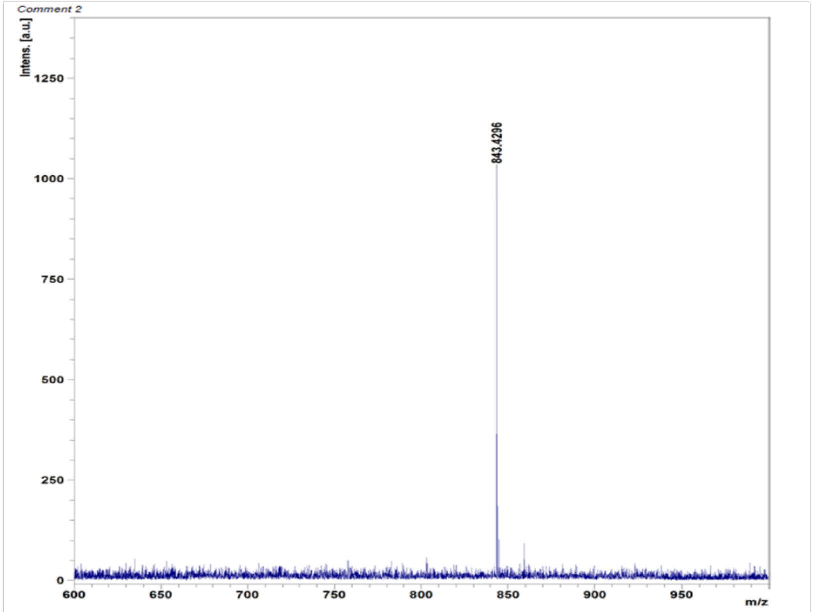

Figure S14. Reaction between Fmoc-YGGSGG-OH and vinyl ether. **A)** Structure and reaction yield of Fmoc-YGGSGG-OH. **B)** HPLC chromatogram. **C)** HR-ESI MS of the product peak. $[M+Na]^+$calcd for $C_{39}H_{44}N_6NaO_{14}^+$ 843.2808found: 843.4296.

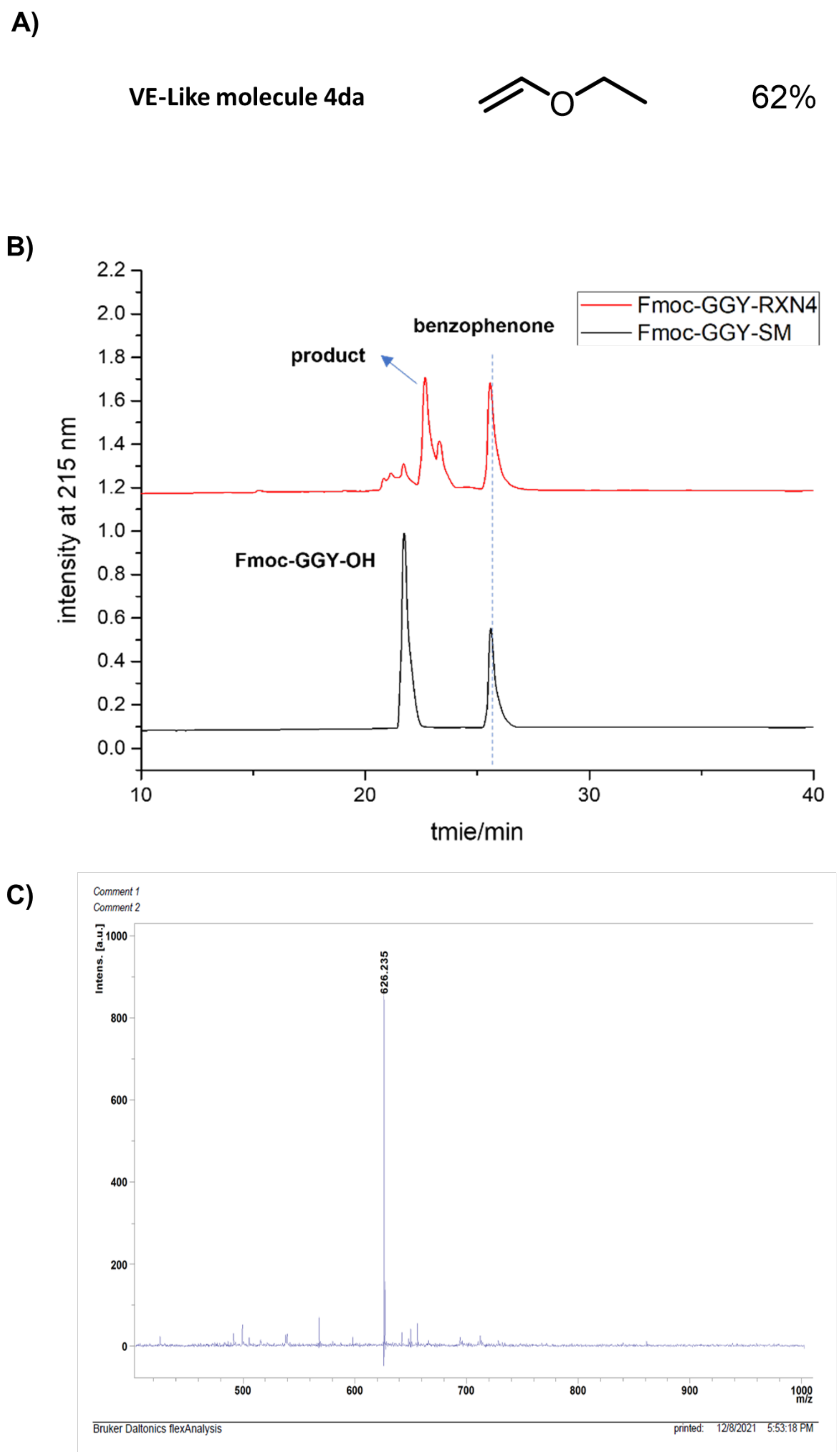

Figure S15. Reaction analysis and yield of **4da**. **A)** Structure and reaction yield of **4da**. **B)** HPLC chromatogram. **C)** HR-ESI MS of the product peak. $[M+Na]^+$calcd for $C_{32}H_{33}N_3NaO_9^+$ 626.211 found: 626.235.

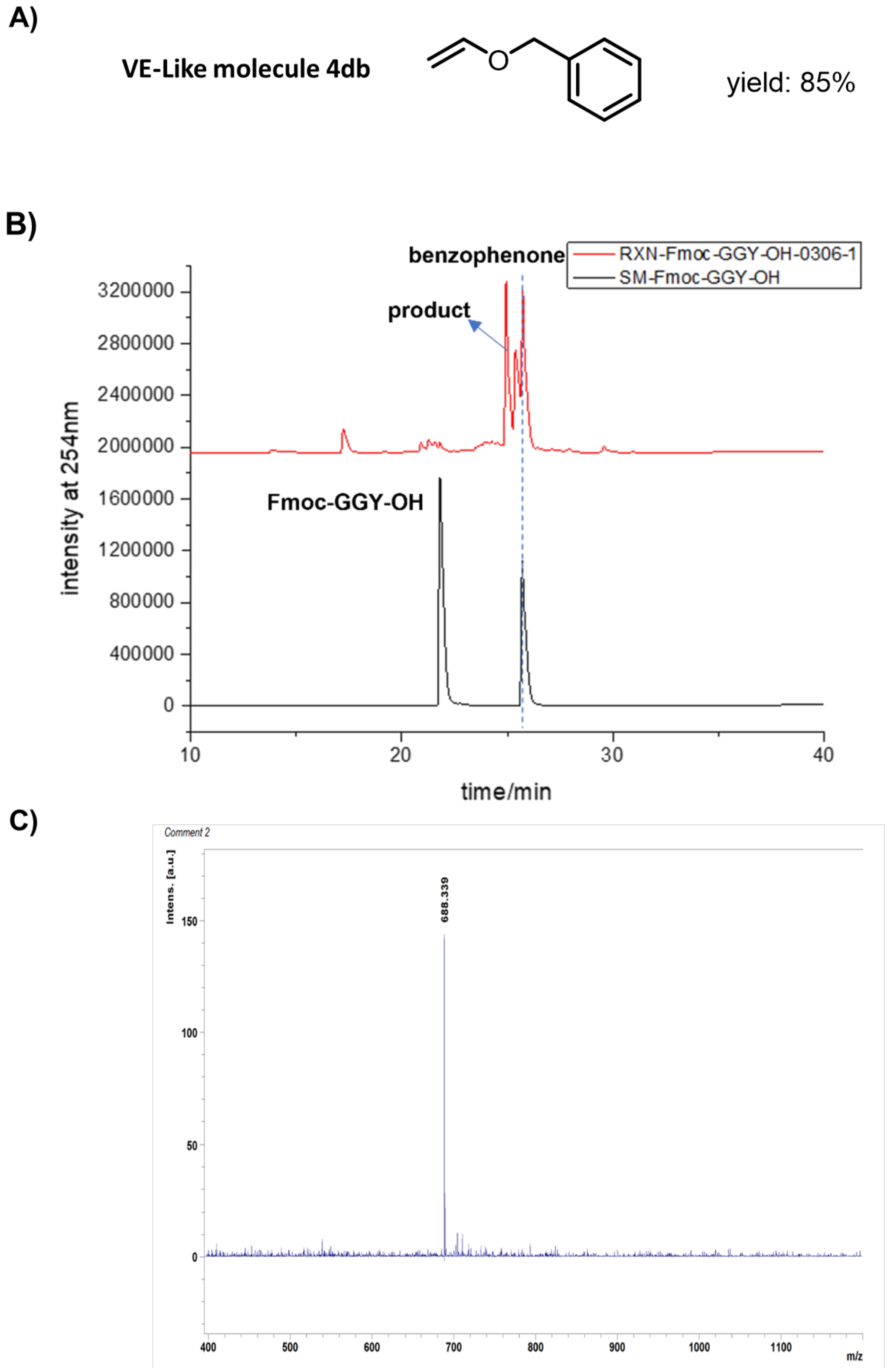

Figure S16. Reaction analysis and yield of **4db**. **A)** Structure and reaction yield of **4db**. **B)** HPLC chromatogram. **C)** HR-ESI MS of the product peak. $[M+Na]^+$calcd for $C_{37}H_{35}N_3NaO_9^+$ 688.227 found:688.339.

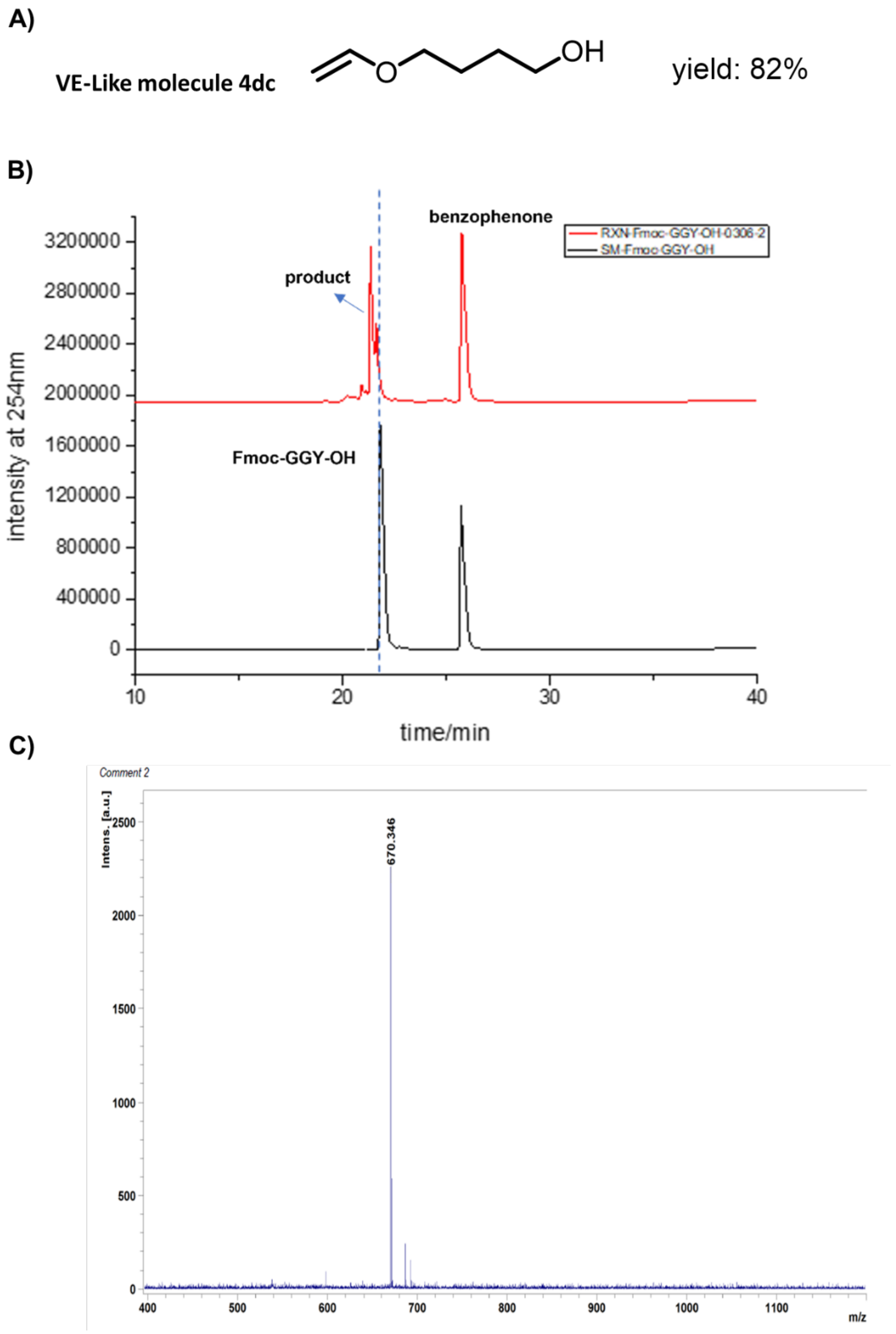

Figure S17. Reaction analysis and yield of **4dc**. **A)** Structure and reaction yield of **4dc**. **B)** HPLC chromatogram. **C)** HR-ESI MS of the product peak. $[M+Na]^+$calcd for $C_{34}H_{37}N_3NaO_{10}^+$ 670.237 found:670.346.

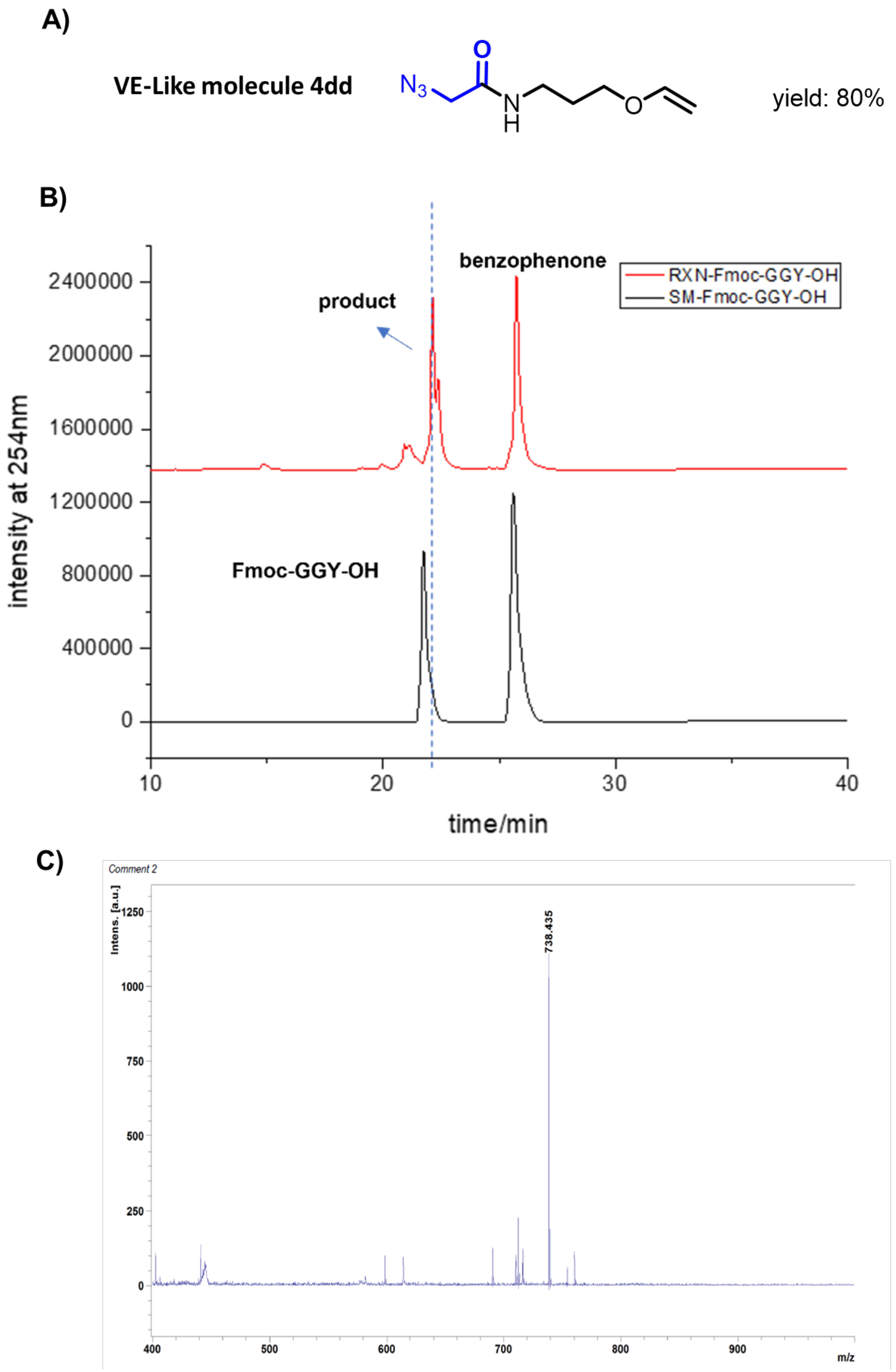

Figure S18. Reaction analysis and yield of **4dd**. **A)** Structure and reaction yield of **4dd**. **B)** HPLC chromatogram. **C)** HR-ESI MS of the product peak. $[M+Na]^+$ calcd for $C_{35}H_{37}N_7NaO_{10}^+$ 738.249 found:738.435.

A)

VE-Like molecule 4de

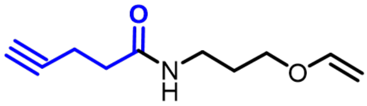

yield: 71%

B)

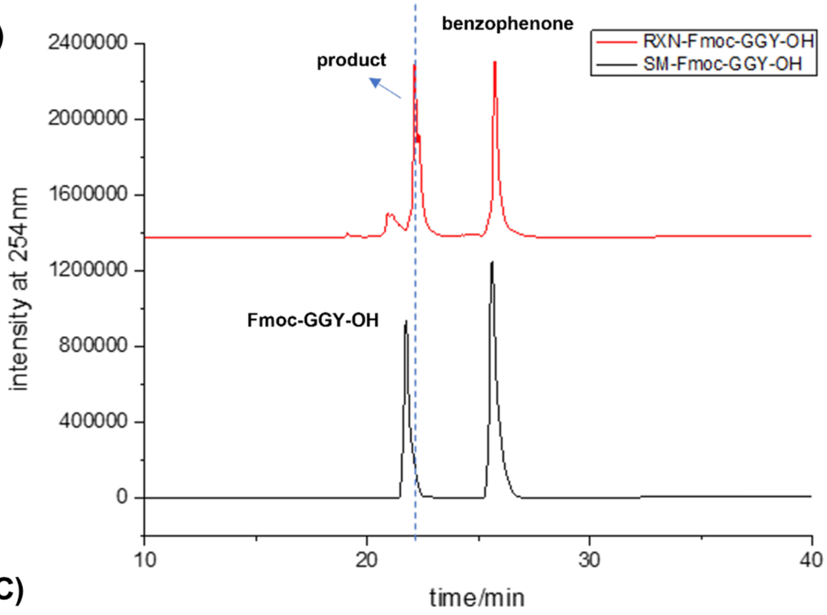

C)

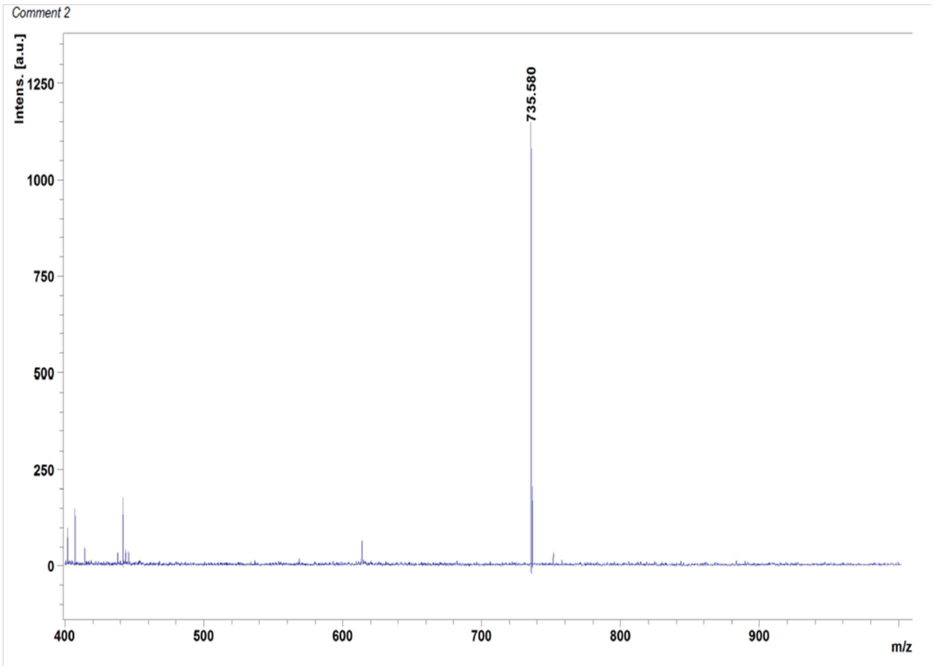

Figure S19. Reaction analysis and yield of **4de**. **A)** Structure and reaction yield of **4de**. **B)** HPLC chromatogram. **C)** HR-ESI MS of the product peak. $[M+Na]^+$ calcd for $C_{35}H_{37}N_7NaO_{10}^+$ 735.264 found:735.580

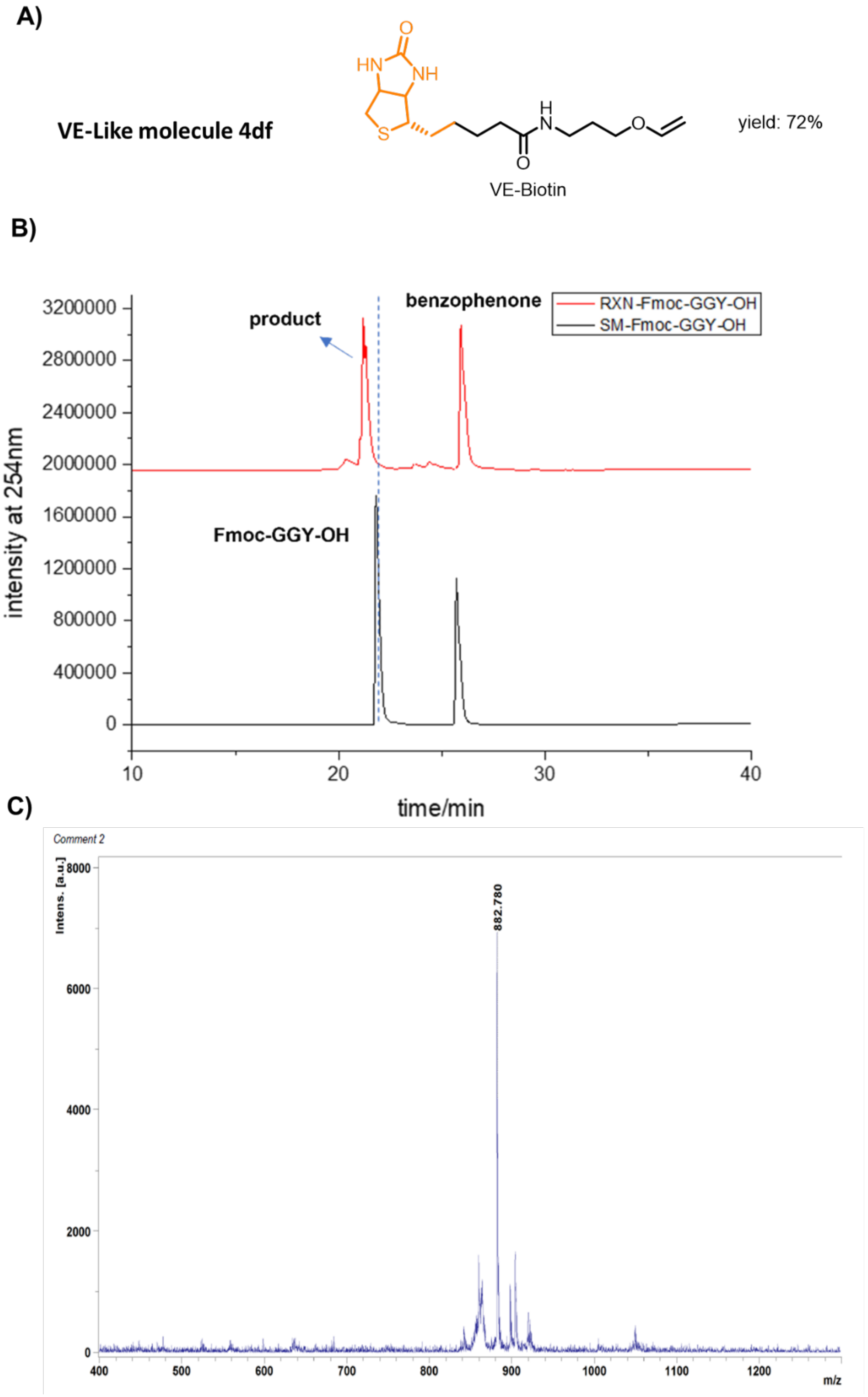

Figure S20. Reaction analysis and yield of **4df**. **A)** Structure and reaction yield of **4df**. **B)** HPLC chromatogram. **C)** HR-ESI MS of the product peak. [M+Na] $^{+}$ calcd for $C_{43}H_{50}N_6NaO_{11}^{+}$ 881.953found:882.780

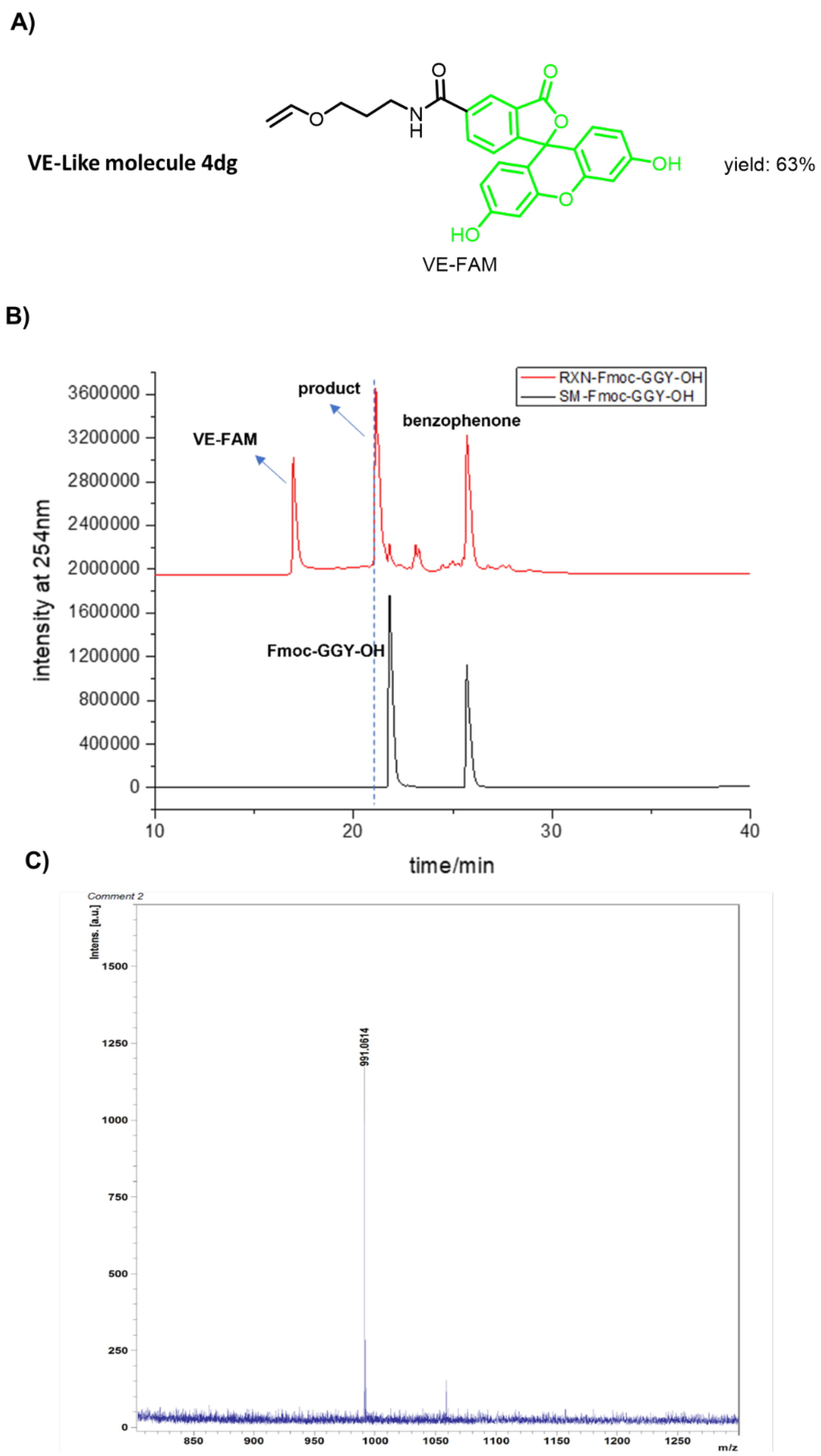

Figure S21. Reaction analysis and yield of **4dg**. **A)** Structure and reaction yield of **4dg**. **B)** HPLC chromatogram. **C)** HR-ESI MS of the product peak. $[M+H]^+$ calcd for $C_{54}H_{47}N_4O_{15}^+$ 991.3032 found:991.0614**.**

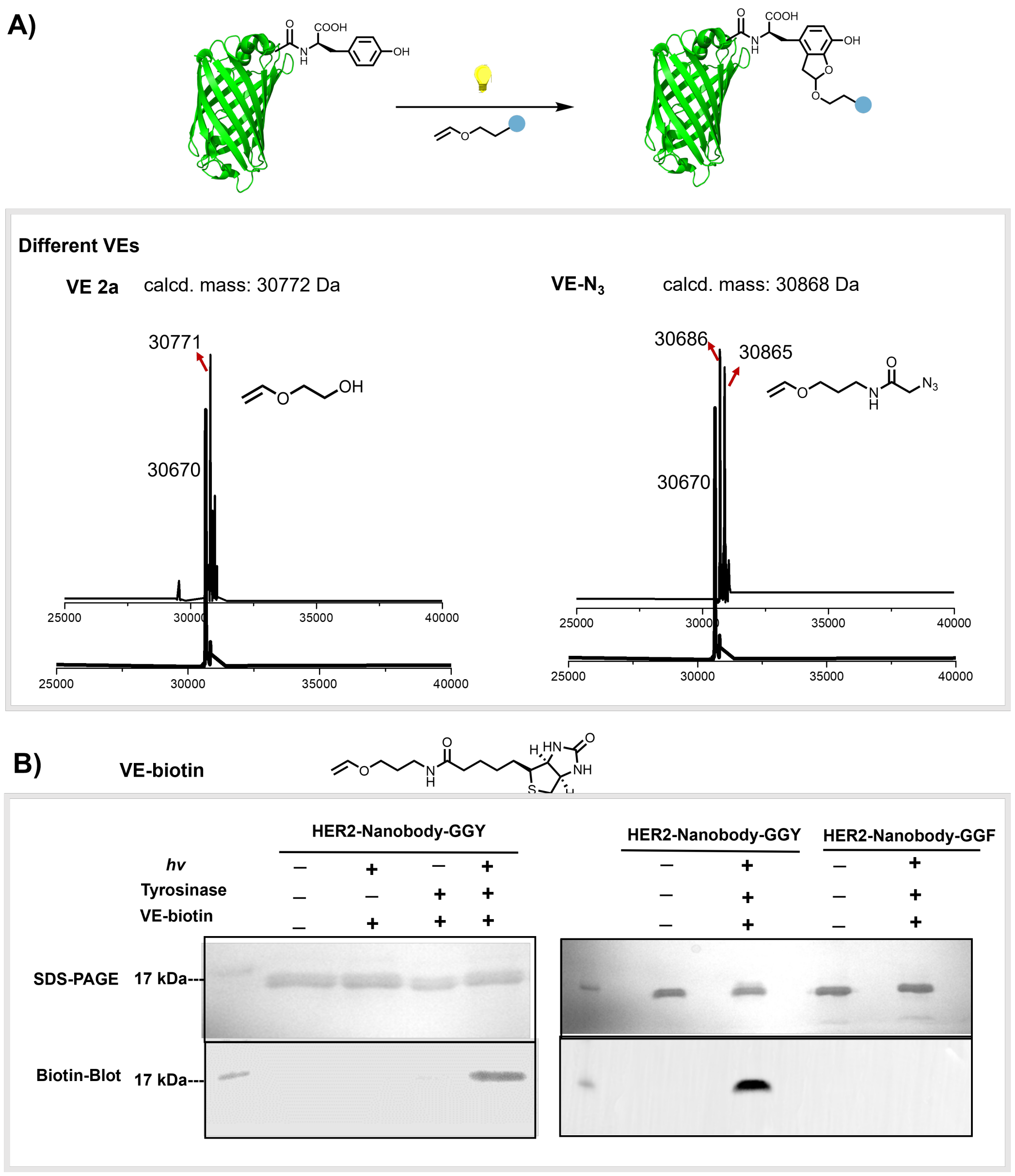

Figure S22. Tyrosine reaction for the functionalization of recombinant proteins. **(A)** Chemoenzymatic photoaddition of VE **2a** and **VE-N$_3$** to GFP-GGY. **(B)** Site-selective biotinylation of nbHER2-GGY. Reaction condition: protein (10 µM), **VE-biotin** (1000 µM), tyrosinase (100 nM) in PB Buffer (200 mM, pH 6.5) irradiated by 456 nm light (20 mW/cm^2) at 4°C for 1 h. The solutions were then resolved by denaturing SDS−polyacrylamide gel electrophoresis (SDS-PAGE) and imaged by Coomassie blue staining and analyzed using HRP-conjugated streptavidin. Raw figures can be found in Figure S23 in the Supporting Information.

A)

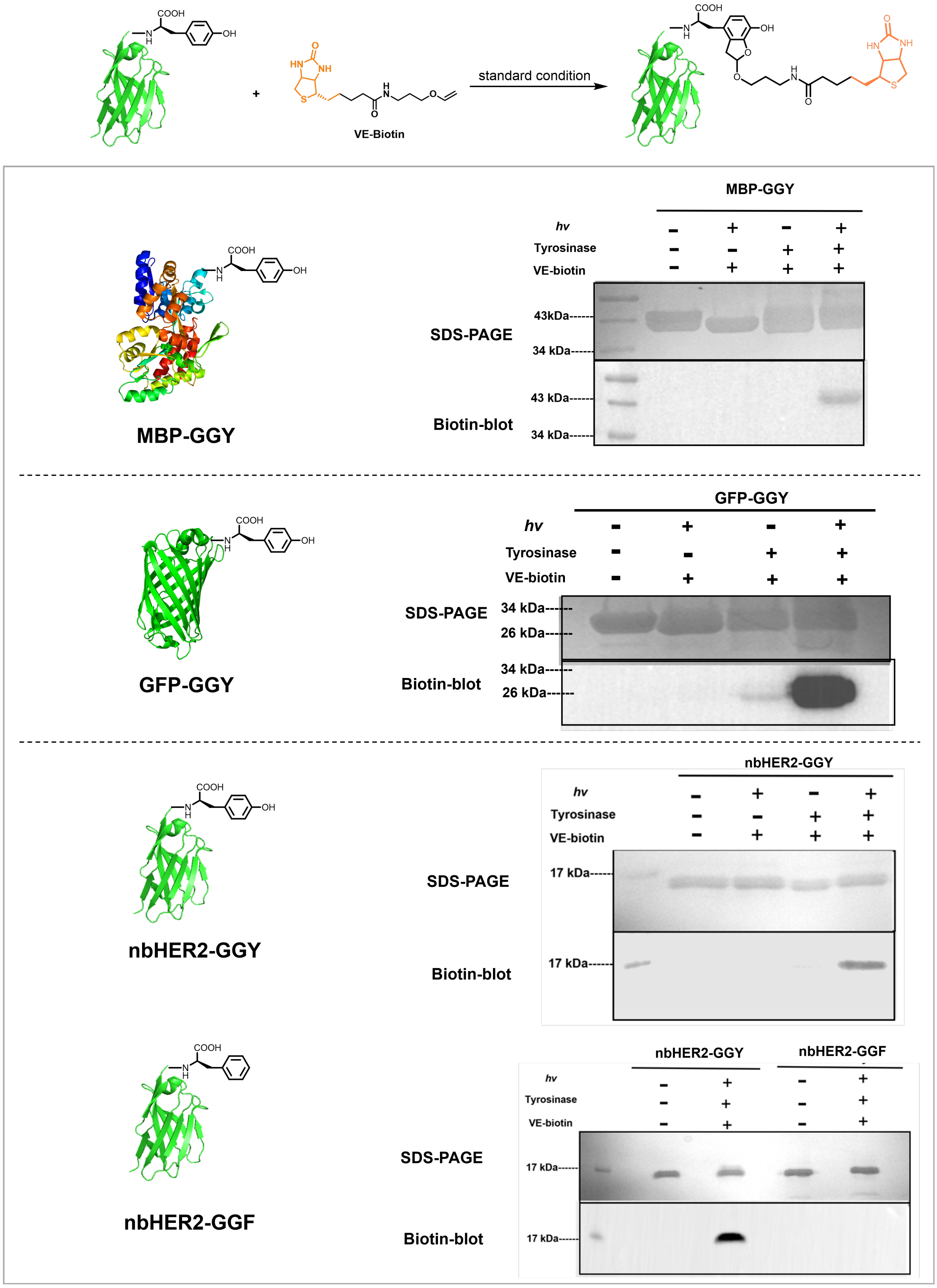
COOH
OH
HN
NH
O
S
VE-Biotin
standard condition
MBP-GGY
hv
Tyrosinase
VE-biotin
SDS-PAGE
Biotin-blot
43kDa
34 kDa
43 kDa
34 kDa
GFP-GGY
26 kDa
nbHER2-GGY
17 kDa
nbHER2-GGF

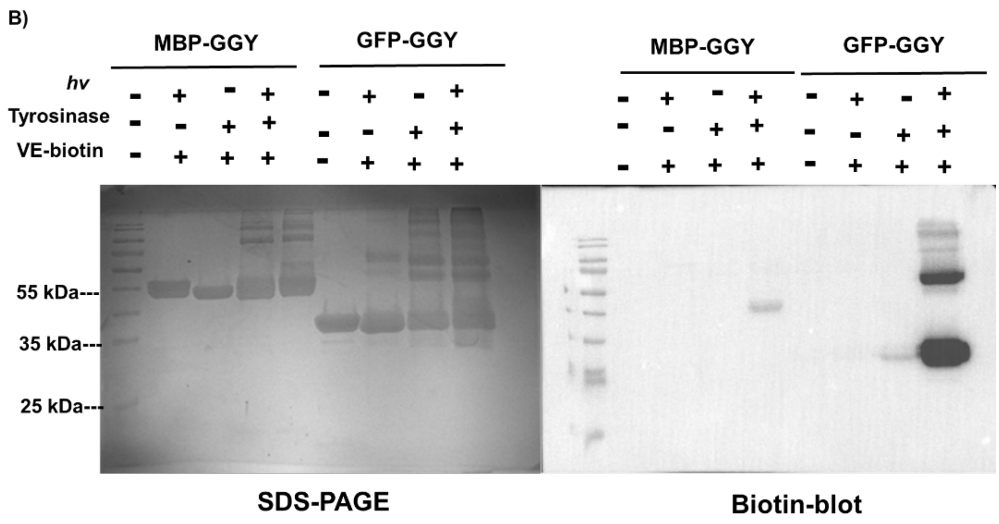

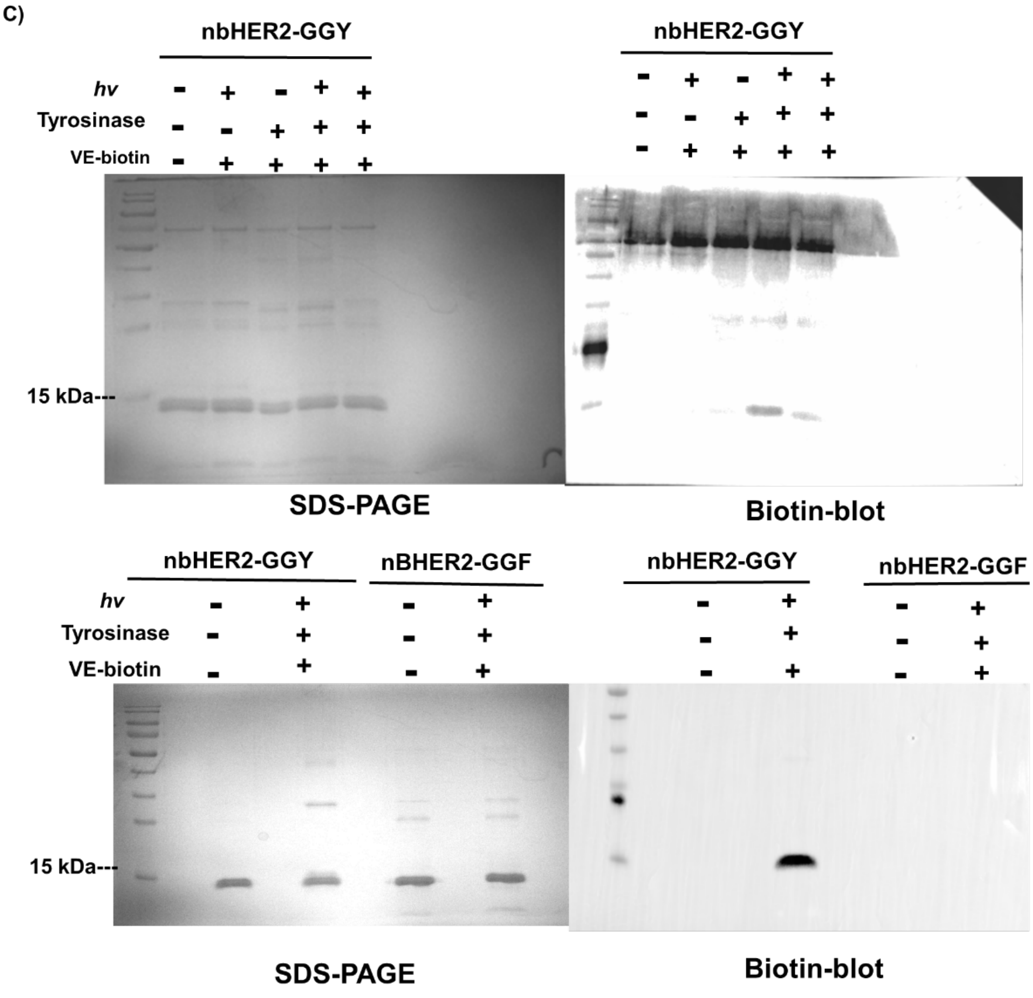

Figure S23. Protein modification of MBP-GGY, GFP-GGY, and nbHER2-GGY. **A)** SDS-PAGE and western blot for biotin after proteins conjugated with VE-Biotin under standard conditions. Reaction condition: protein (10 μM) was incubated with tyrosinase (0.1 μM) and VE-biotin (1000 μM) in PB

buffer (0.2 M, pH 6.5) at 4°C under 456 nm irradiation (Kessil lamp, 20 mw/cm^2) for 60 min. The reaction was analyzed by SDS-PAGE and western blotting. **B)** Raw SDS-PAGE and Biotin-Blot figures for modification of MBP-GGY and GFP-GGY. **C)** Raw SDS-PAGE and Biotin-Blot figures for modification nbHER2-GGY and nbHER2-GGF.

A)

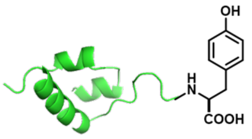

nbHER2-GGY

Protein sequence:

MGSSHHHHHHSSGLVPRGSHMQVQLQESGGGSVQAGGS
LKLTCAASGYIFNSCGMGWYRQSPGRERELVSRISGDGDT
WHKESVKGRFTISQDNVKKTLYLQMNSLKPEDTAVYFCA
VCYNLETYWGQGTQVTVSSGGGGSGGY

B)

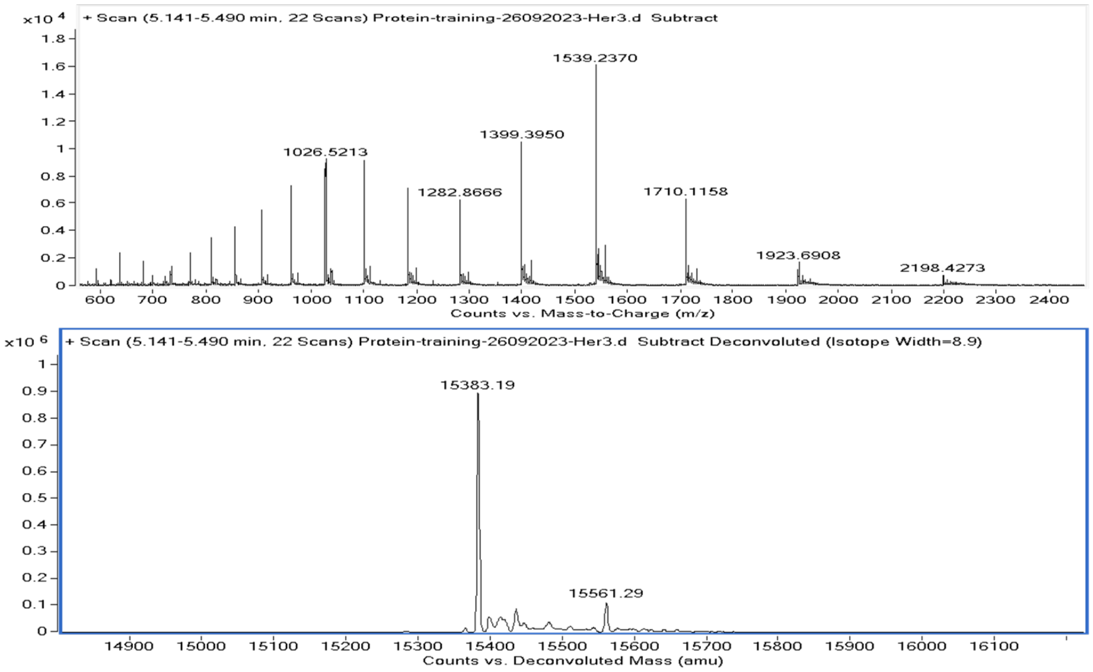

Figure S24. Characterization of nbHER2-GGY. **A)** Protein sequence of nbHER2-GGY. **B)** LC-MS analysis of nbHER2-GGY protein. Calculated (-Met) 15385 Da, found 15383 Da.

A)

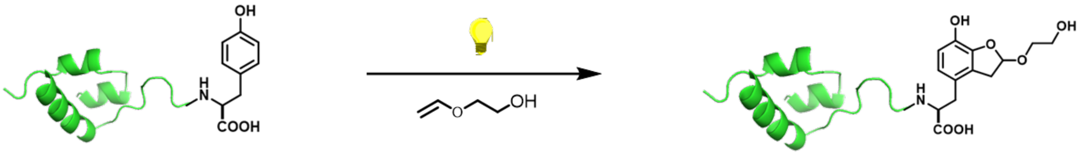

B)

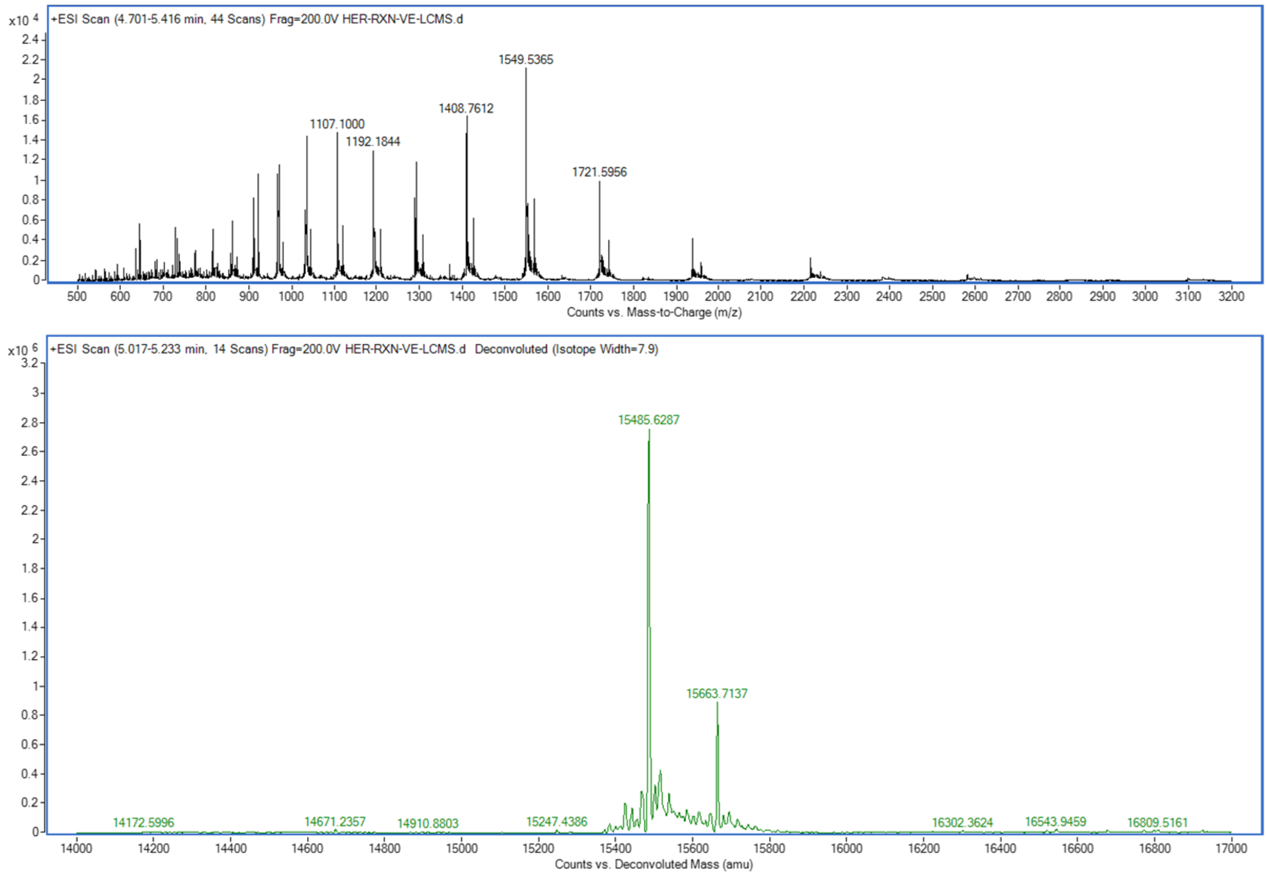

Figure S25. Modification and characterization of nbHER2-GGY with vinyl ether. **A)** Scheme of nbHER2-GGY modification with vinyl ether. **B)** LC-MS analysis of modified nbHER2-GGY protein. Calculated 15485 Da, found 15485 Da.

A)

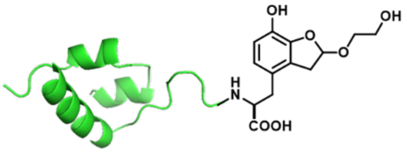

nbHER2-GGY-VE

B)

File	Score (Bio)	RT	Mass	Vol	Sequence	Seq Loc
R-RXN-VE-MSMS.d	30	3.922	1656.7229	43874	GQGTQVTVSSGGGGSGGY	A(126-143)
R-RXN-VE-MSMS.d	28.81	4.327	1842.8045	279804	WGQGTQVTVSSGGGGSGGY	A(125-143)
R-RXN-VE-MSMS.d	28.96	4.794	2463.0856	38530	NLETYWGQGTQVTVSSGGGGSGGY	A(120-143)
R-RXN-VE-MSMS.d	29.55	4.878	2463.0846	462314	NLETYWGQGTQVTVSSGGGGSGGY	A(120-143)
R-RXN-VE-MSMS.d	29.9	2.333	181.0741	79852	Y	A(57-57)
R-RXN-VE-MSMS.d	27.55	3.393	181.0748	16350	Y	A(57-57)
R-RXN-VE-MSMS.d	41.72	3.515	278.1637	437380	IF	A(48-49)
R-RXN-VE-MSMS.d	29.97	3.795	278.1632	21701	IF	A(48-49)
R-RXN-VE-MSMS.d	60.82	3.836	671.2422	1579192	CAVCY	A(115-119)
R-RXN-VE-MSMS.d	29.92	3.842	181.074	41388	Y	A(57-57)
R-RXN-VE-MSMS.d	68.21	3.858	638.2927	932820	NLETY	A(120-124)
R-RXN-VE-MSMS.d	38.96	3.929	1408.7597	1491028	TISQDNVKKTLY	A(88-99)
R-RXN-VE-MSMS.d	27.71	4.318	1623.7846	169260	LQMNSLKPEDTAVY	A(100-113)
R-RXN-VE-MSMS.d	29.11	4.367	1740.7724	65743	WGQGTQVTVSSGGGGSGGY	A(125-143)
R-RXN-VE-MSMS.d	51.02	4.532	818.3111	989418	FCAVCY	A(114-119)
R-RXN-VE-MSMS.d	29.56	4.532	842.2695	27508	NSCGMGW	A(50-56)
R-RXN-VE-MSMS.d	48.48	4.627	1607.7919	2812365	LQMNSLKPEDTAVY	A(100-113)
R-RXN-VE-MSMS.d	29.78	4.806	973.3429	96656	NSCGMGWY	A(50-57)
R-RXN-VE-MSMS.d	22.73	4.835	2998.542	25400	TISQDNVKKTLYLQMNSLKPEDTAVY	A(88-113)

C)

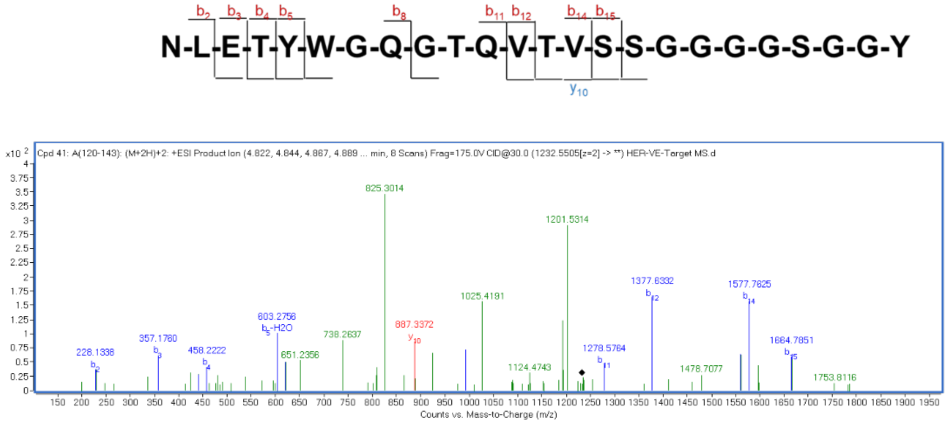

Abundance	m/z calculated	m/z detected	Ion	Sequence	Z
38.1	228.1343	228.1338	b2	NL	1
62.8	357.1769	357.1760	b3	NLE	1
40.9	458.2245	458.2222	b4	NLET	1
50.6	621.2879	621.2823	b5	NLETY	1
71.7	992.4472	992.4434	b8	NLETYWGQ	1
44.3	1278.5749	1278.5764	b11	NLETYWGQGTQ	1
163	1377.6434	1377.6332	b12	NLETYWGQGTQV	1
157.3	1577.7595	1577.7625	b14	NLETYWGQGTQVTV	1
58.8	1664.7915	1664.7851	b15	NLETYWGQGTQVTVS	1
87	887.3377	887.3372	y10	SSGGGGSGGY	1
29.1	440.214	440.210	b4-H_2O	NLET	1
100.6	603.2273	603.2756	b5-H_2O	NLETY	1
64.2	1559.7489	1559.7468	b14-H_2O	NLETYWGQGTQVTV	1

Figure S26. LC-MS/MS characterization of the reaction site of nbHER2-GGY-VE. **A)** structure of nbHER2-GGY modification with vinyl ether. **B)** Peptide mapping of modified nbHER2 digested by chymotrypsin. Because the C-terminus of nbHER2 lacks Lys or Arg, we chose chymotrypsin instead of trypsin for digestion. **C)** LC-MS/MS analysis of modified nbHER2 identified a modified C-terminal peptide that contains only one single Tyr, which indicated that only the terminal tyrosine was modified.

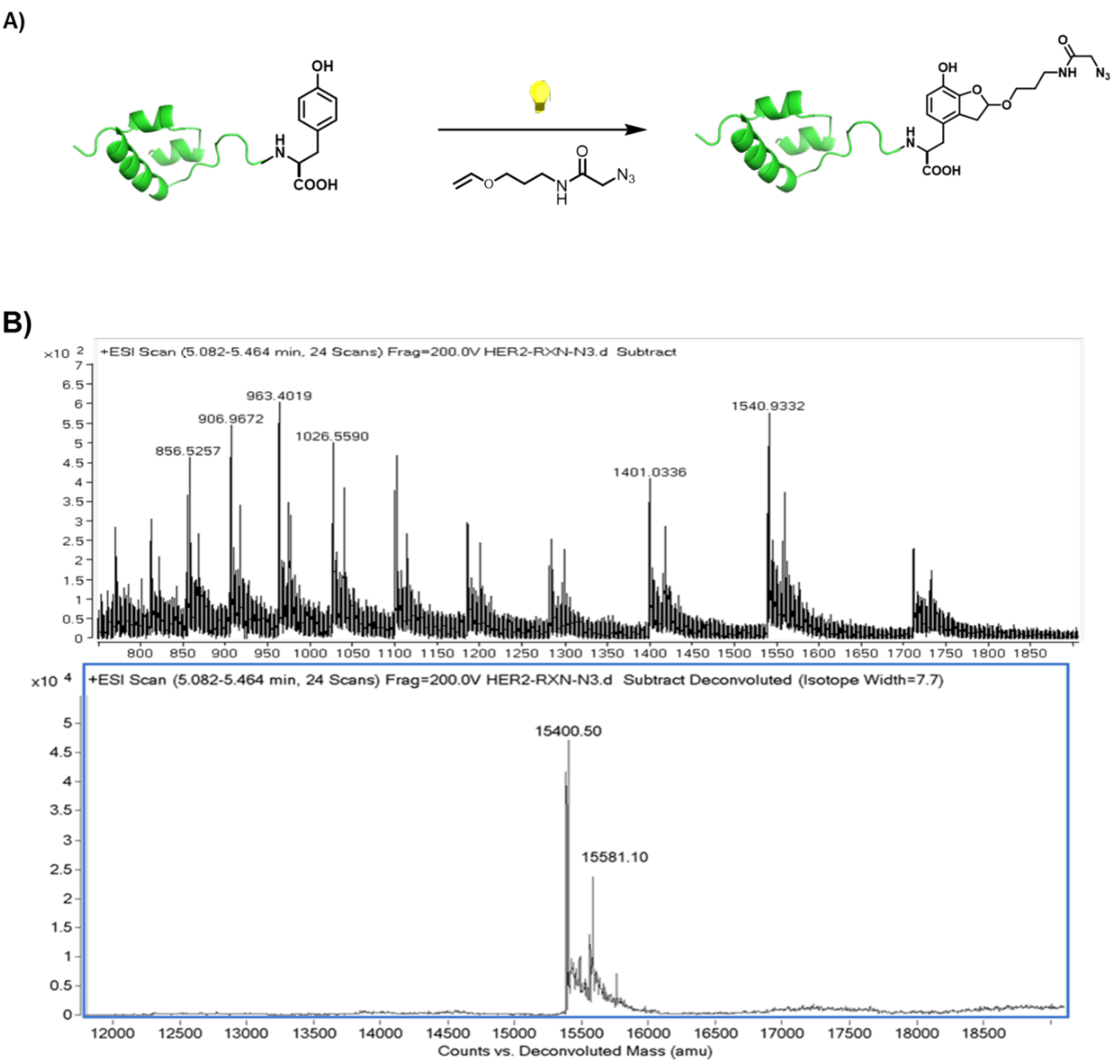

Figure S27. Modification and characterization of nbHER2-GGY with vinyl ether azide. **A)** Scheme of nbHER2-GGY modification with vinyl ether azide. **B)** LC-MS analysis of modified nbHER2-GGY protein. Calculated 15581 Da, found 15581 Da.

A)

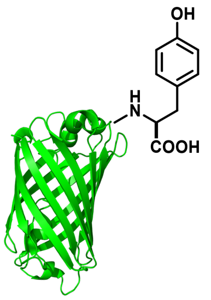

GFP-GGY

protein sequences:

MHHHHHHMASMTGGQQMGRGSMVSKGEELFTGVVPILVEL
DGDVNGHKFSVSGEGEGDATYGKLTLKFICTTGKLPVPWPTL
VTTLTYGVQCFSRYPDHMKQHDFFKSAMPEGYVQERTIFFKD
DGNYKTRAEVKFEGDTLVNRIELKGIDFKEDGNILGHKLEYN
YNSHNVYIMADKQKNGIKVNFKIRHNIEDGSVQLADHYQQNT
PIGDGPVLLPDNHYLSTQSALSKDPNEKRDHMVLLEFVTAAGI
TLGMDELYKELRRQASGGGGSGGY*

B)

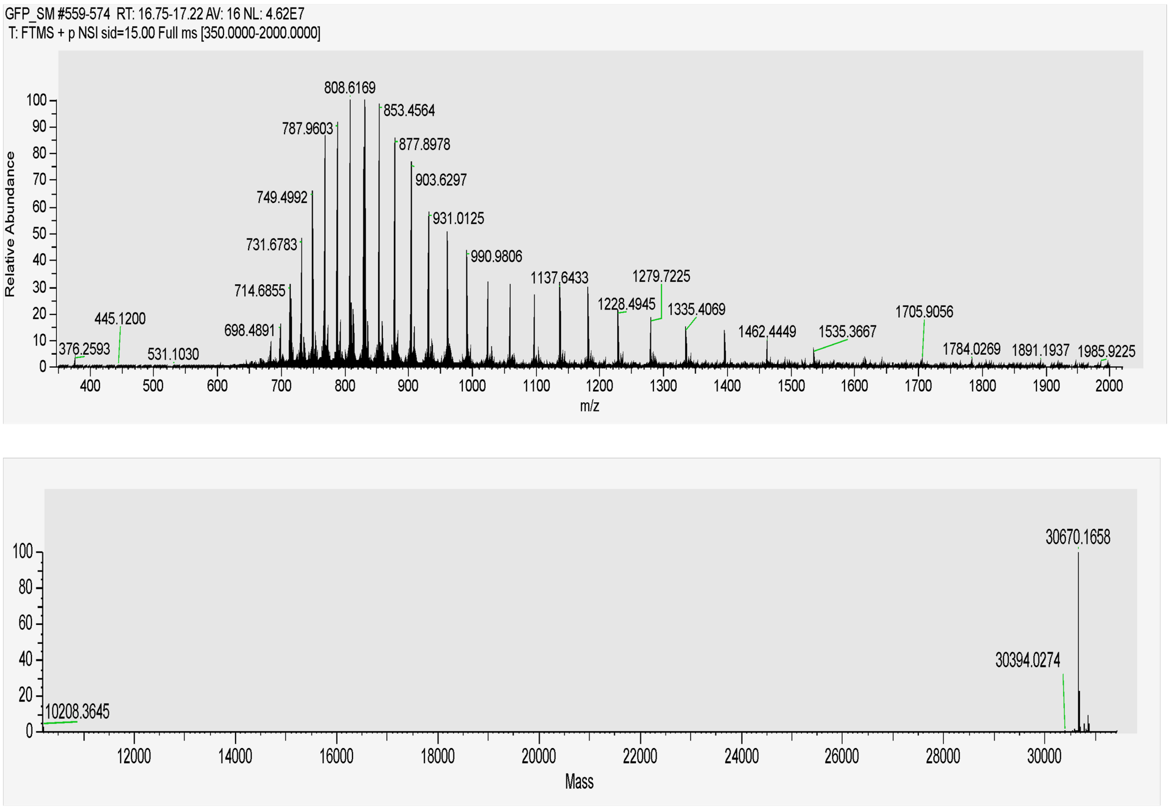

Figure S28. MS analysis of GFP-GGY. **A)** Protein sequence of GFP-GGY. **B)** LC-MS analysis of GFP-GGY protein. Calculated 30670 Da, found 30670 Da.

A)

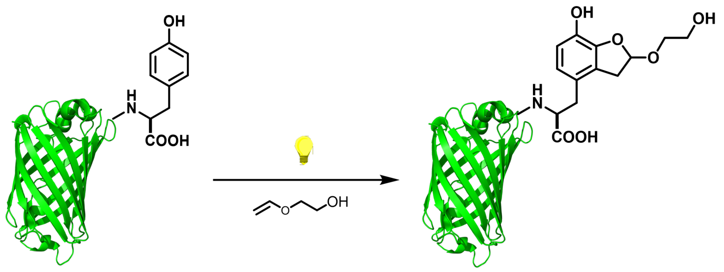

B)

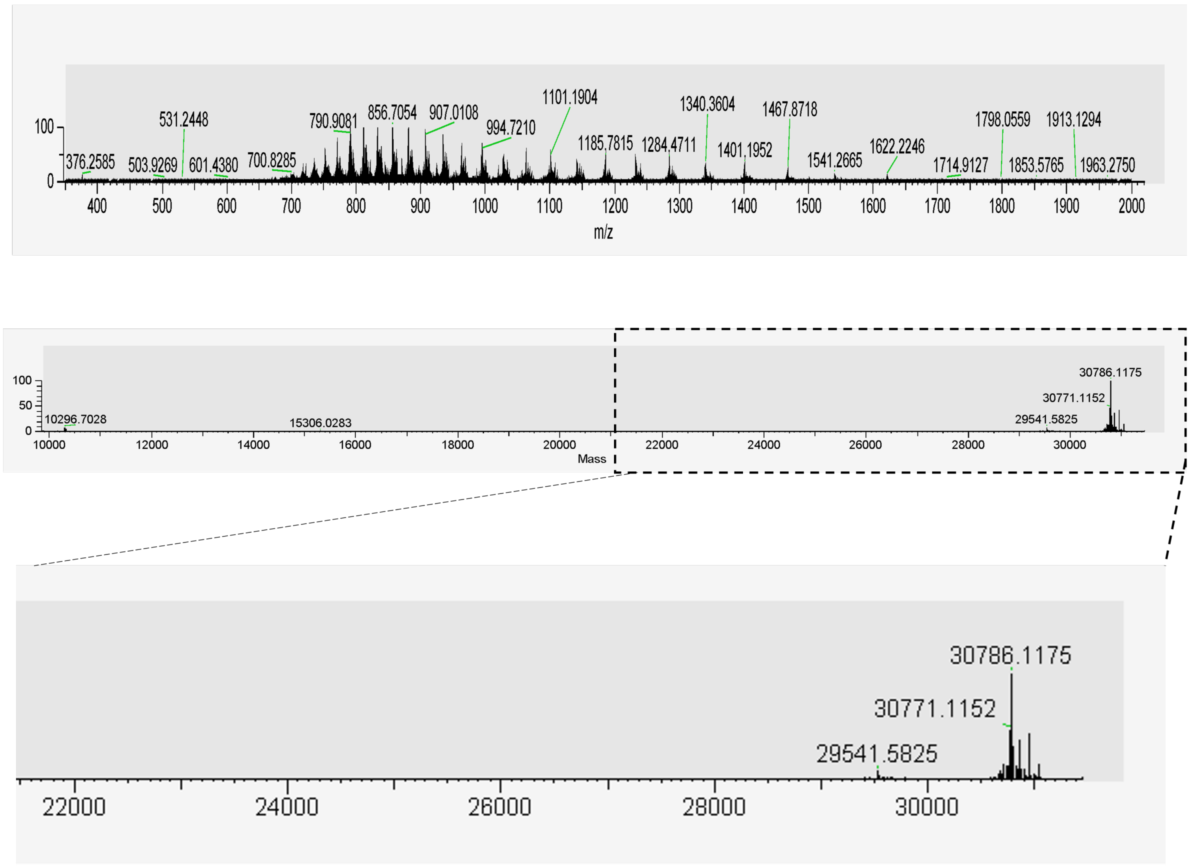

Figure S29. MS analysis of the reaction product of GFP-GGY with vinyl ether. **A)** Scheme of GFP-GGY modification with vinyl ether. **B)** LC-MS analysis of modified GFP-GGY protein. Calculated 30772 Da, found 30771 Da.

A)

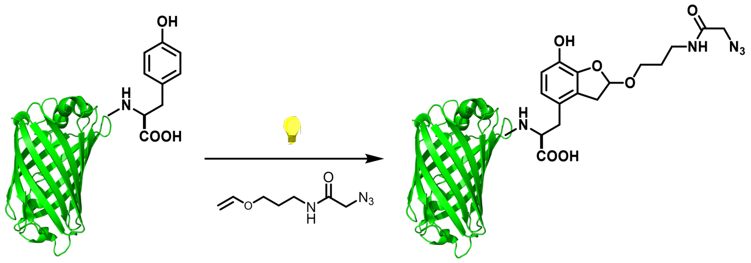

B)

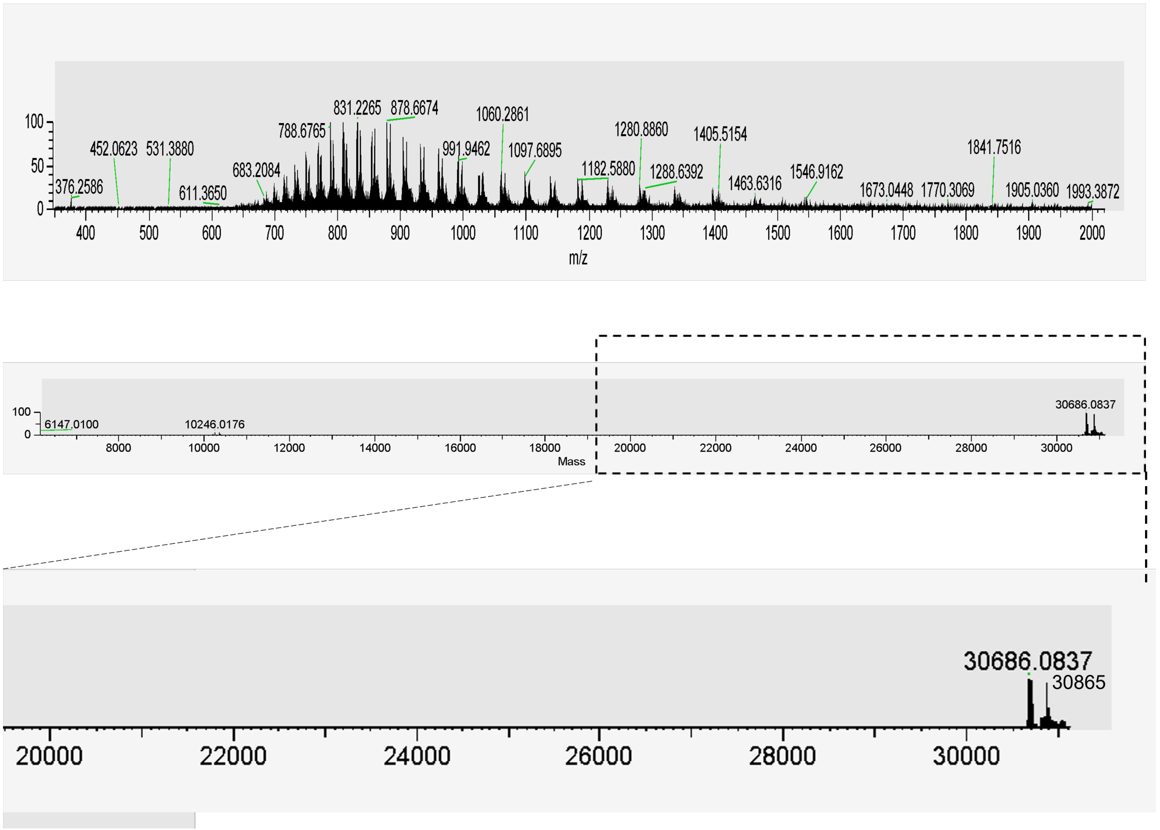

Figure S30. Modification and characterization of GFP-GGY with vinyl ether azide. **A)** Scheme of GFP-GGY modification with vinyl ether azide. **B)** LC-MS analysis of modified GFP-GGY protein. Calculated 30868 Da, found 30865 Da.

A)

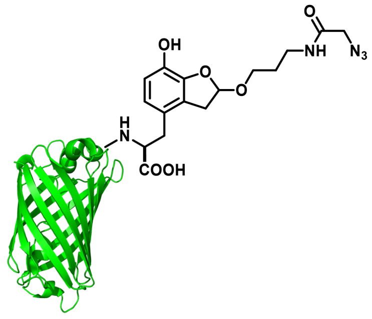

GFP-GGY-VE-N_3

B)

Peptide Summary

Fragment Matches

Value Type: Theo. Mass [Da]

Ion Series | Neutral Losses | Precursor Ions | Internal Fragments

#1	b-CO+	b-CO2+	b-H2O+	b-H2O2+	b-NH3+	b-NH32+	Seq.	y-CO+	y-CO2+	y-H2O+	y-H2O2+	y-NH3+	y-NH32+	#2
1	129.11347	65.06037			140.08184	70.54456	R							12
2	257.17205	129.08966			268.14042	134.57385	Q	1067.45008	534.22868	1077.43443	539.22085	1078.41844	539.71286	11
3	328.20916	164.60822			339.17753	170.09240	A	939.39150	470.19939	949.37585	475.19156			10
4	415.24119	208.12423	425.22554	213.11641	426.20956	213.60842	S	868.35439	434.68083	878.33873	439.67301			9
5	472.26266	236.63497	482.24701	241.62714	483.23102	242.11915	G	781.32236	391.16482	791.30671	396.15699			8
6	529.28412	265.14570	539.26847	270.13787	540.25249	270.62988	G	724.30089	362.65408	734.28524	367.64626			7
7	586.30558	293.65643	596.28993	298.64861	597.27395	299.14061	G	667.27943	334.14335	677.26378	339.13553			6
8	643.32705	322.16716	653.31140	327.15934	654.29541	327.65134	G	610.25797	305.63262	620.24232	310.62480			5
9	730.35908	365.68318	740.34343	370.67535	741.32744	371.16736	S	553.23650	277.12189	563.22085	282.11406			4
10	787.38054	394.19391	797.36489	399.18608	798.34890	399.67809	G	466.20447	233.60588					3
11	844.40200	422.70464	854.38635	427.69681	855.37037	428.18882	G	409.18301	205.09514					2
12							Y-CUHK-H...	352.16155	176.58441					1

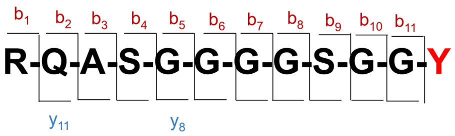

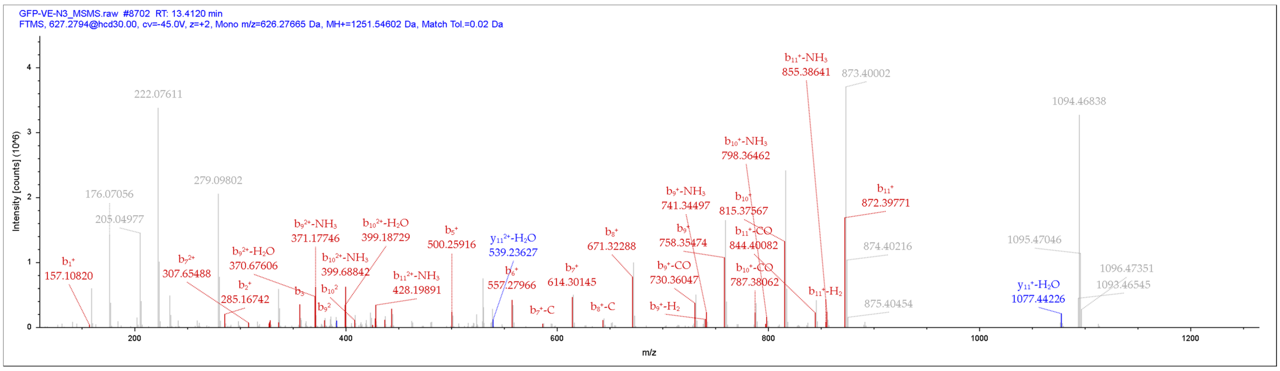

Figure S31. LC-MS/MS analysis of GFP-GGY-VE-N_3. **A)** Structure of GFP-GGY modification with vinyl ether azide. **B)** LC-MS/MS analysis of GFP-GGY-VE-N_3 suggests that the terminal Tyr is the reaction site.

A)

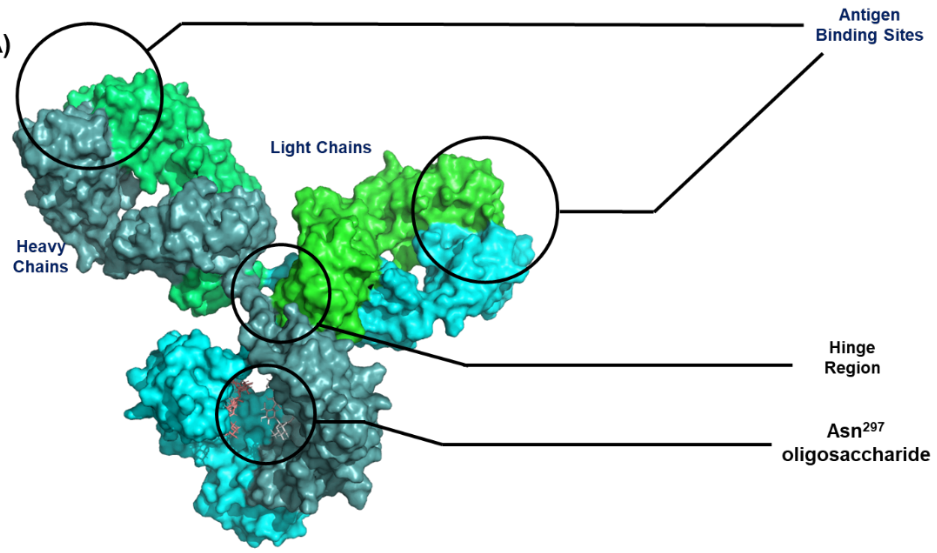

B)

Trastuzumab:

Heavy chain:

1 **60**
EVQLVESGGG LVQPGGSLRL SCAASGFNIK DTYIHWVRQA PGKGLEWVAR IYPTNGYTRY
61 **120**
ADSVKGRFTI SADTSKNTAY LQMNSLRAED TAVYYCSRWG GDGFYAMDYW GQGTLVTVSS
121 **180**
ASTKGPSVFP LAPSSKSTSG GTAALGCLVK DYFPEPVTVS WNSGALTSGV HTFPAVLQSS
181 **240**
GLYSLSSVVT VPSSSLGTQT YICNVNHKPS NTKVDKKVEP KSCDKTHTCP PCPAPELLGG
241 **300**
PSVFLFPPKP KDTLMISRTP EVTCVVVDVS HEDPEVKFNW YVDGVEVHNA KTKPREEQYN
301 **360**
STYRVVSVLT VLHQDWLNGK EYKCKVSNKA LPAPIEKTIS KAKGQPREPQ VYTLPPSREE
361 **420**
MTKNQVSLTC LVKGFYPSDI AVEWESNGQP ENNYKTTPPV LDSDGSFFLY SKLTVDKSRW
421
QQGNVFSCSV MHEALHNHYT QKSLSLSPG

C)

Atezolizumab:

Heavy chain:

1 **60**
EVQLVESGGG LVQPGGSLRL SCAASGFTFS DSWIHWVRQA PGKGLEWVAW ISPYGGSTYY
61 **120**
ADSVKGRFTI SADTSKNTAY LQMNSLRAED TAVYYCARRH WPGGFDYWGQ GTLVTVSSAS
121 **180**
TKGPSVFPLA PSSKSTSGGT AALGCLVKDY FPEPVTVSWN SGALTSGVHT FPAVLQSSGL
181 **240**
YSLSSVVTVP SSSLGTQTYI CNVNHKPSNT KVDKKVEPKS CDKTHTCPPC PAPELLGGPS
241 **300**
VFLFPPKPKD TLMISRTPEV TCVVVDVSHE DPEVKFNWYV DGVEVHNAKT KPREEQYAST
301 **360**
YRVVSVLTVL HQDWLNGKEY KCKVSNKALP APIEKTISKA KGQPREPQVY TLPPSREEMT
361 **420**
KNQVSLTCLV KGFYPSDIAV EWESNGQPEN NYKTTPPVLD SDGSFFLYSK LTVDKSRWQQ
421
GNVFSCSVMH EALHNHYTQK SLSLSPGK

D)

Daratumumab:

Heavy chain:

```
1                                                             60
EVQLLESGGG LVQPGGSLRL SCAVSGFTFN SFAMSWVRQA PGKGLEWVSA ISGSGGGTYY
61                                                           120
ADSVKGRFTI SRDNSKNTLY LQMNSLRAED TAVYFCAKDK ILWFGEPVFD YWGQGTLVTV
121                                                          180
SSASTKGPSV FPLAPSSKST SGGTAALGCL VKDYFPEPVT VSWNSGALTS GVHTFPAVLQ
181                                                          240
SSGLYSLSSV VTVPSSSLGT QTYICNVNHK PSNTKVDKRV EPKSCDKTHT CPPCPAPELL
241                                                          300
GGPSVFLFPP KPKDTLMISR TPEVTCVVVD VSHEDPEVKF NWYVDGVEVH NAKTKPREEQ
301                                                          360
YNSTYRVVSV LTVLHQDWLN GKEYKCKVSN KALPAPIEKT ISKAKGQPRE PQVYTLPPSR
361                                                          420
EEMTKNQVSL TCLVKGFYPS DIAVEWESNG QPENNYKTTP PVLDSDGSFF LYSKLTVDKS
421
RWQQGNVFSC SVMHEALHNH YTQKSLSLSP GK
```

E)

Cetuximab:

Heavy chain:

```
1                                                             60
QVQLKQSGPG LVQPSQSLSI TCTVSGFSLT NYGVHWVRQS PGKGLEWLGV IWSGGNTDYN
61                                                           120
TPFTSRLSIN KDNSKSQVFF KMNSLQSNDT AIYYCARALT YYDYEFAYWG QGTLVTVSAA
121                                                          180
STKGPSVFPL APSSKSTSGG TAALGCLVKDYFPEPVTVSW NSGALTSGVH TFPAVLQSSG
181                                                          240
LYSLSSVVTV PSSSLGTQTY ICNVNHKPSN TKVDKRVEPK SCDKTHTCPP CPAPELLGGP
241                                                          300
SVFLFPPKPK DTLMISRTPE VTCVVVDVSH EDPEVKFNWY VDGVEVHNAK TKPREEQYNS
301                                                          360
TYRVVSVLTV LHQDWLNGKE YKCKVSNKAL PAPIEKTISK AKGQPREPQV YTLPPSREEM
361                                                          420
TKNQVSLTCL VKGFYPSDIA VEWESNGQPE NNYKTTPPVL DSDGSFFLYS KLTVDKSRWQ
421
QGNVFSCSVM HEALHNHYTQ KSLSLSPGK
```

Figure S32. Sequence comparison of human IgG1 heavy chains. **A)** Structure of human IgG1. **B)** Heavy chain sequence of Trastuzumab. **C)** Heavy chain sequence of Atezolizumab. **D)** Heavy chain sequence of Daratumumab. **E)** Heavy chain sequence of Cetuximab.

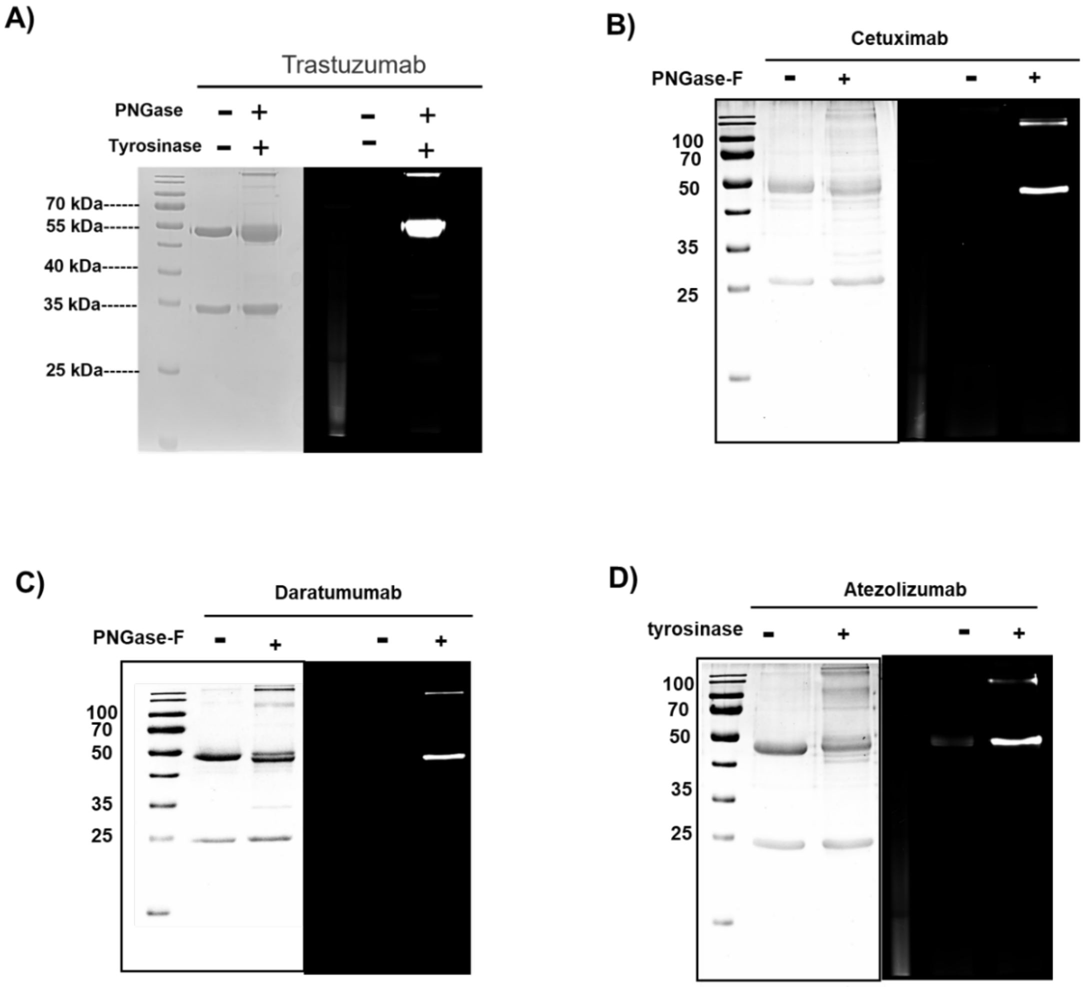
A)
Trastuzumab
PNGase
Tyrosinase
70 kDa
55 kDa
40 kDa
35 kDa
25 kDa
B)
Cetuximab
PNGase-F
100
70
50
35
25
C)
Daratumumab
PNGase-F
100
70
50
35
25
D)
Atezolizumab
tyrosinase
100
70
50
35
25

E)

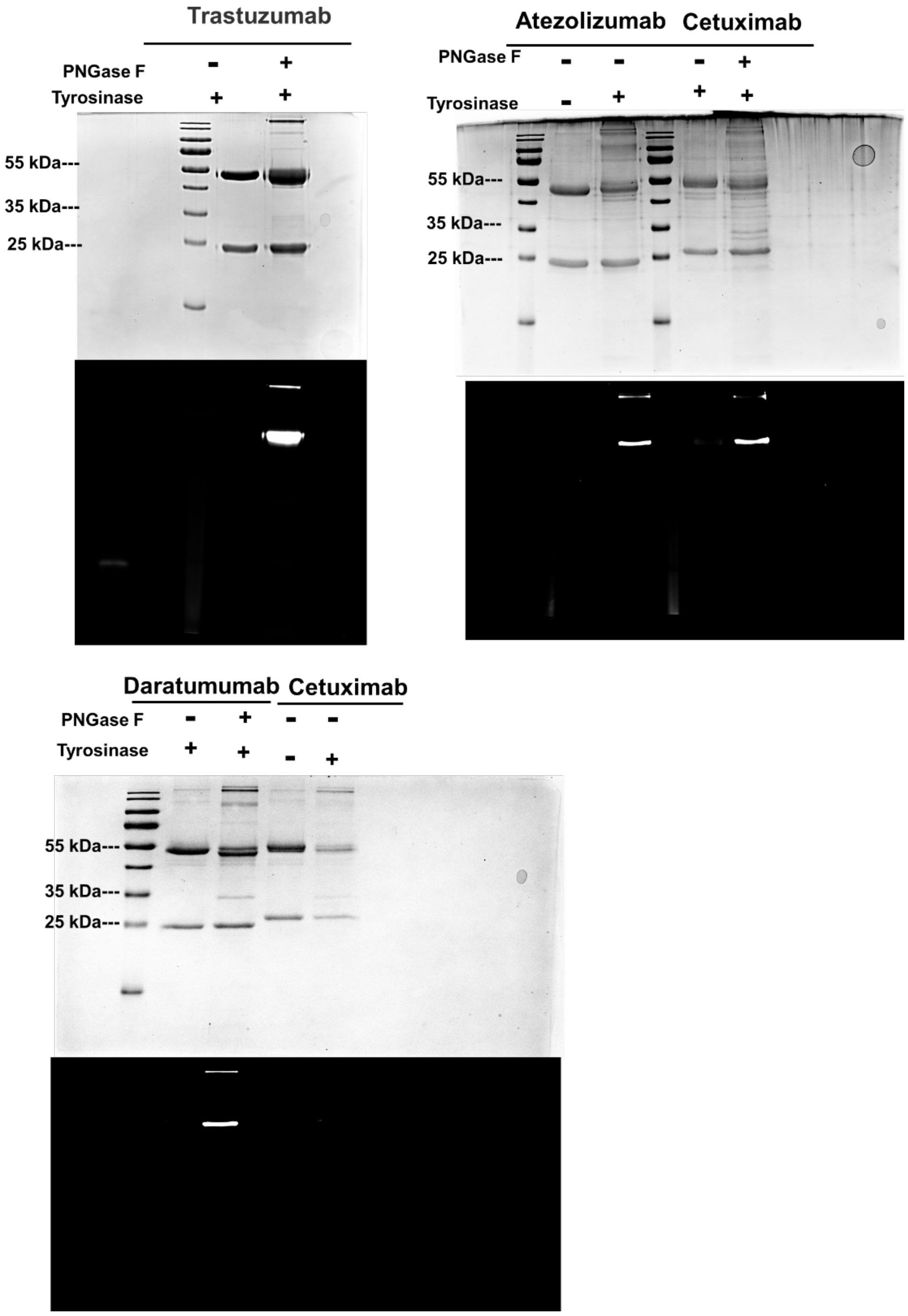

Figure S33. Heavy chain modification of Human IgG1. **A)** SDS-PAGE and gel-fluorescence analysis of the reaction of Trastuzumab. **B)** SDS-PAGE and gel-fluorescence analysis of the reaction of Cetuximab. **C)** SDS-PAGE and gel-fluorescence analysis of the reaction of Daratumumab. **D)** SDS-PAGE and gel-fluorescence analysis of the reaction of Atezolizumab. **E)** Raw SDS-PSGE and gel-fluorescence figures for modification of Trastuzumab, Cetuximab, Daratumumab and Atezolizumab.

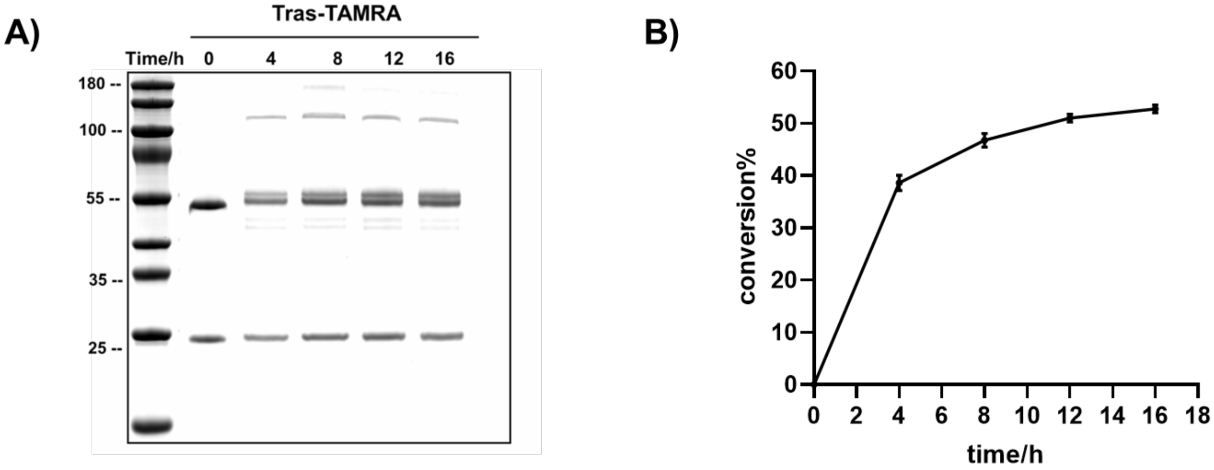

Figure S34. Kinetics of the antibody reaction. **A)** SDS-PAGE analysis of modified antibody. **B)** Reaction conversion analysis of antibody modification. Reaction condition: deglycosylated trastuzumab (5 µM) was diluted with 20 µL PB buffer pH 6.5 and incubated with VE-N_3 (500 µM) and mushroom tyrosinase (2 µM) and irradiated under 456 nm light (20 mW/cm^2) for different reaction periods at 4 °C. After completion, the product was concentrated, and small molecules were removed by 30 kDa MWCO filters. After that, DBCO-PEG$_4$-TAMRA (5 µL, 2 mM in DMSO) was added and incubated at room temperature for 1.5 h. Product conversion was quantified based on the band shift in the SDS-PAGE image. Data are presented as the mean ± s.d. of n = 3 independent experiments.

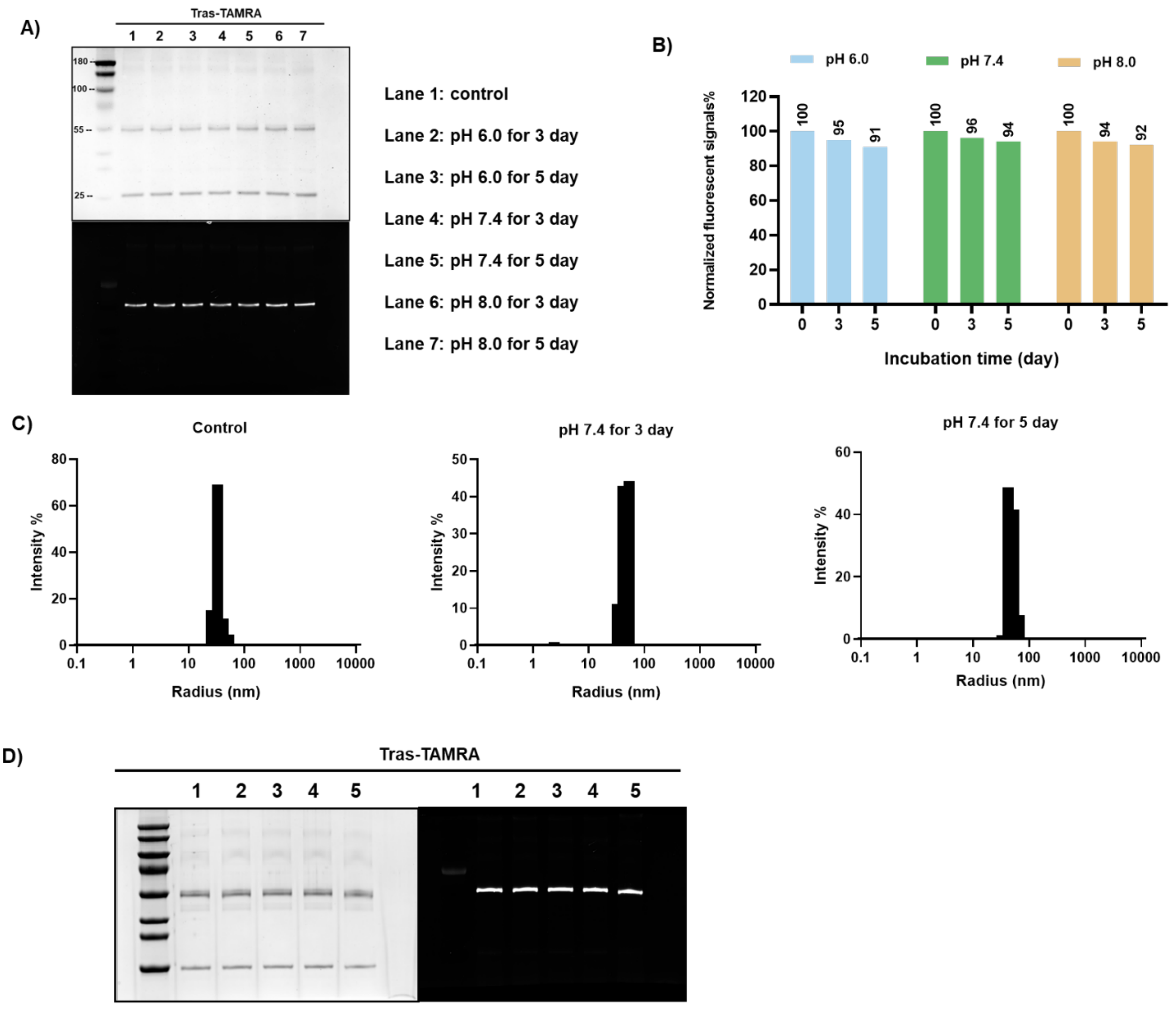

Lane 1: PBS Buffer (Phosphate buffered saline, pH 7.4, 137 mM NaCl, 2.7 mM KCl, 10 mM Na_2HPO_4, 1.8 mM KH_2PO_4)

Lane 2: Tris Buffer (pH 7.4 50 mM Tris-HCl)

Lane 3: HB (Homogenization Buffer, pH 7.4, 10 mM HEPES, 0.25 M Sucrose, 1 mM EDTA)

Lane 4: HEPES Buffer (pH 7.4, 10 mM HEPES, 1 mM EDTA)

Lane 5: PB buffer (Phosphate buffer, pH 7.4, 75 mM Na_2HPO_4, 25 mM NaH_2PO_4)

Figure S35. Chemical modification did not cause significant degradation or aggregation the antibody. **A)** SDS-PAGE of chemically modified antibody Tras-TAMRA incubated in buffers of different pH for three or five days. **B)** Normalized band signals of the fluorescent bands in (A) showing only a marginal decrease. **C)** DLS analyses of the modified antibody at 1.0 mg/ml in PBS (pH 7.4) showing only slight aggregation after five days. **D)** SDS-PAGE showing that different buffer conditions did not cause degradation of the modified antibody.

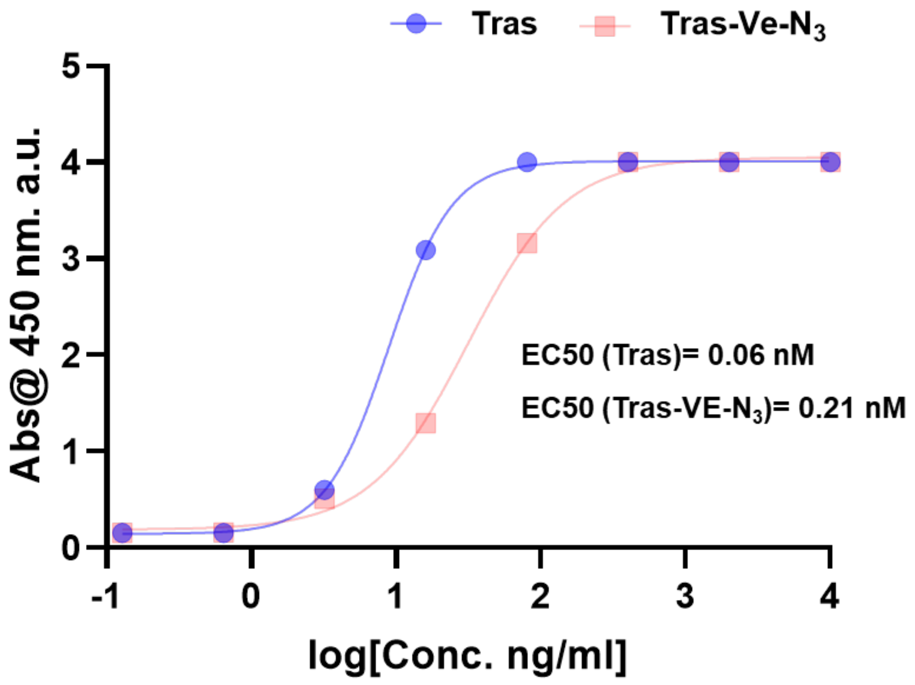

Figure S36. Antigen binding ability of antibodies by ELISA. Briefly, HER2 protein (from Sinobiological Inc., Cat: 10004-H08H4) was coated on the 96-well plate using an ELISA kit (Bethyl Laboratories, E101). Then, Trastuzumab (Tras) and modified Trastuzumab (Tras-VE-N_3) of different concentrations were incubated in the wells. Goat Anti-Human IgG conjugated with HRP (Abclonal, AS002) was used as a secondary antibody, and 3,3',5,5'-Tetramethylbenzidine (TMB) solution was added to indicate the binding efficiency. Data are presented as the mean ± s.d. of n = 3 independent experiments.

A) **Heavy chain of Trastuzumab:**

EVQLVESGGG	LVQPGGSLRL	SCAASGFNIK	DTYIHWVRQA	PGKGLEWVAR	IYPTNGYTRY
ADSVKGRFTI	SADTSKNTAY	LQMNSLRAED	TAVYYCSRWG	GDGFYAMDYW	GQGTLVTVSS
ASTKGPSVFP	LAPSSKSTSG	GTAALGCLVK	DYFPEPVTVS	WNSGALTSGV	HTFPAVLQSS
GLYSLSSVVT	VPSSSLGTQT	YICNVNHKPS	NTKVDKKVEP	KSCDKTHTCP	PCPAPELLGG
PSVFLFPPKP	KDTLMISRTP	EVTCVVVDVS	HEDPEVKFNW	YVDGVEVHNA	KTKPREEQYN
STYRVVSVLT	VLHQDWLNGK	EYKCKVSNKA	LPAPIEKTIS	KAKGQPREPQ	VYTLPPSREE
MTKNQVSLTC	LVKGFYPSDI	AVEWESNGQP	ENNYKTTPPV	LDSDGSFFLY	SKLTVDKSRW
QQGNVFSCSV	MHEALHNHYT	QKSLSLSPG			

B)

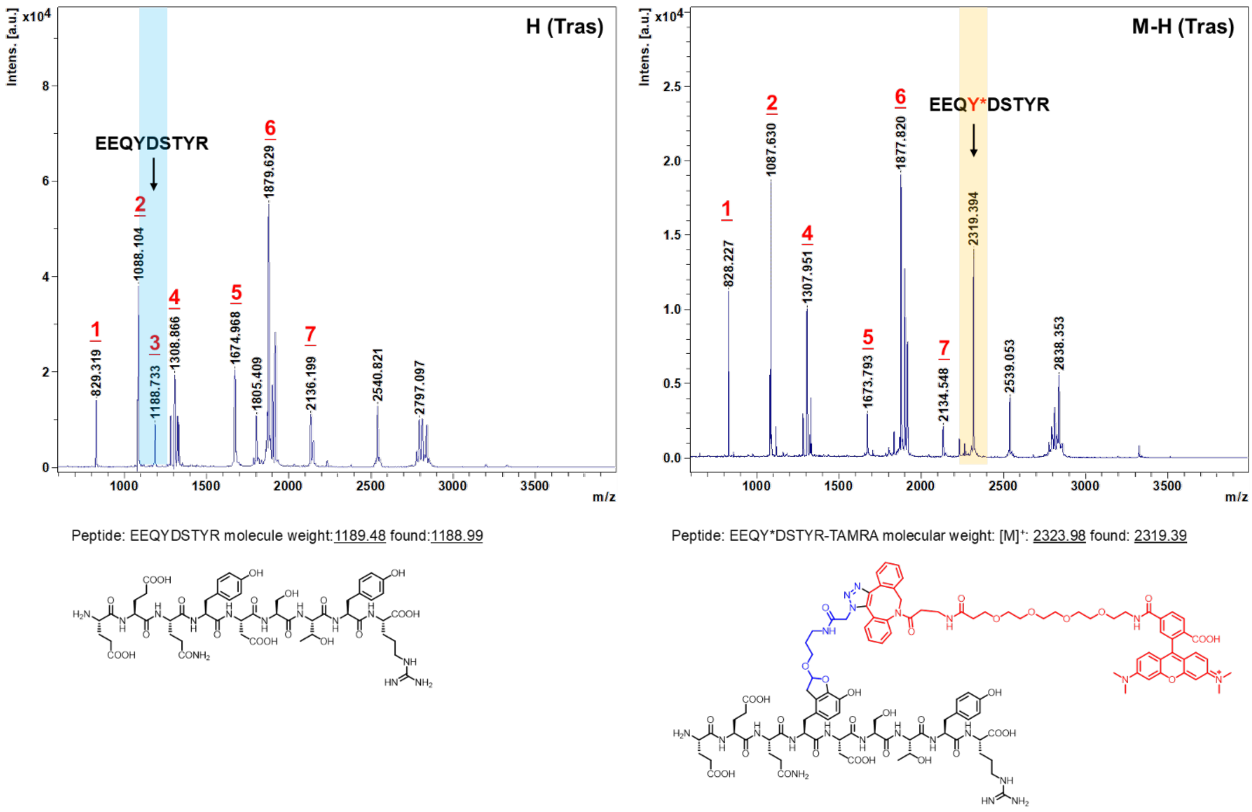

Figure S37. MALDI-TOF MS analysis of enzyme-digested TAMRA-labeled Trastuzumab (Tras-VE-TAMRA). **A)** Amino acid sequence of the heavy chain of Trastuzumab. **B)** MALDI-TOF MS analysis of digested antibody. Peptide fragments of unmodified Tras and Tras-VE-TAMRA were produced by tryptic digestion. In the high-m/z region, a new peak matching the molecular weight of EEQY*DSQYR (Y* in red represents Tyr 296 modified with DBCO-PEG$_4$-TAMRA) was found. The structures are shown above; the calculated exact mass of $[M]^+$: 2323.98, found: 2319.39.

A)

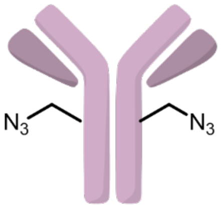

Atezo-VE-N$_3$

B)

Peptide Summary

Fragment Matches

Value Type: Theo. Mass [Da]

Ion Series | Neutral Losses | Precursor Ions | Internal Fragments

#1	b⁺	b²⁺	b³⁺	Seq.	y⁺	y²⁺	y³⁺	#2
1	130.04987	65.52857	44.02147	E				9
2	259.09246	130.04987	87.03567	E	1215.53889	608.27308	405.85115	8
3	387.15104	194.07916	129.72186	Q	1086.49630	543.75179	362.83695	7
4	748.28966	374.64847	250.10140	Y-CUHK-H...	958.43772	479.72250	320.15076	6
5	819.32677	410.16702	273.78044	A	597.29910	299.15319	199.77122	5
6	906.35880	453.68304	302.79112	S	526.26199	263.63463	176.09218	4
7	1007.40648	504.20688	336.47368	T	439.22996	220.11862	147.08150	3
8	1170.46981	585.73854	390.82812	Y	338.18228	169.59478	113.39894	2
9				R	175.11895	88.06311	59.04450	1

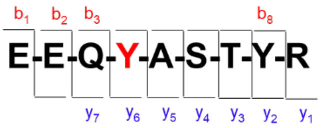

Tyr 296 of Atezolizumab is modified

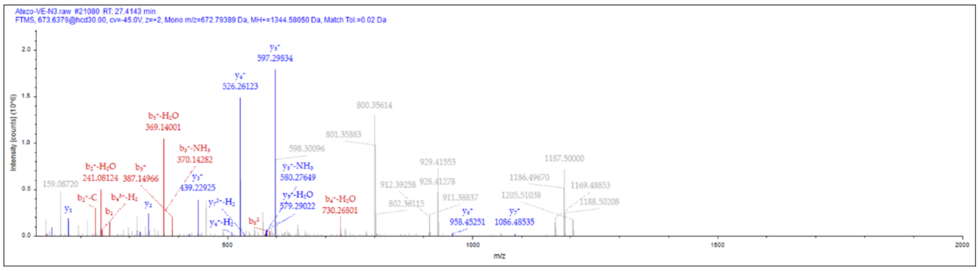

Figure S38. LC-MS/MS characterization of reaction site of Atezo-N$_3$. **A)** Structure of Atezolizumab (Atezo) modification with vinyl ether azide. **B)** LC-MS/MS analysis of modified Atezolizumab suggests that Tyr 296 is the reaction site.

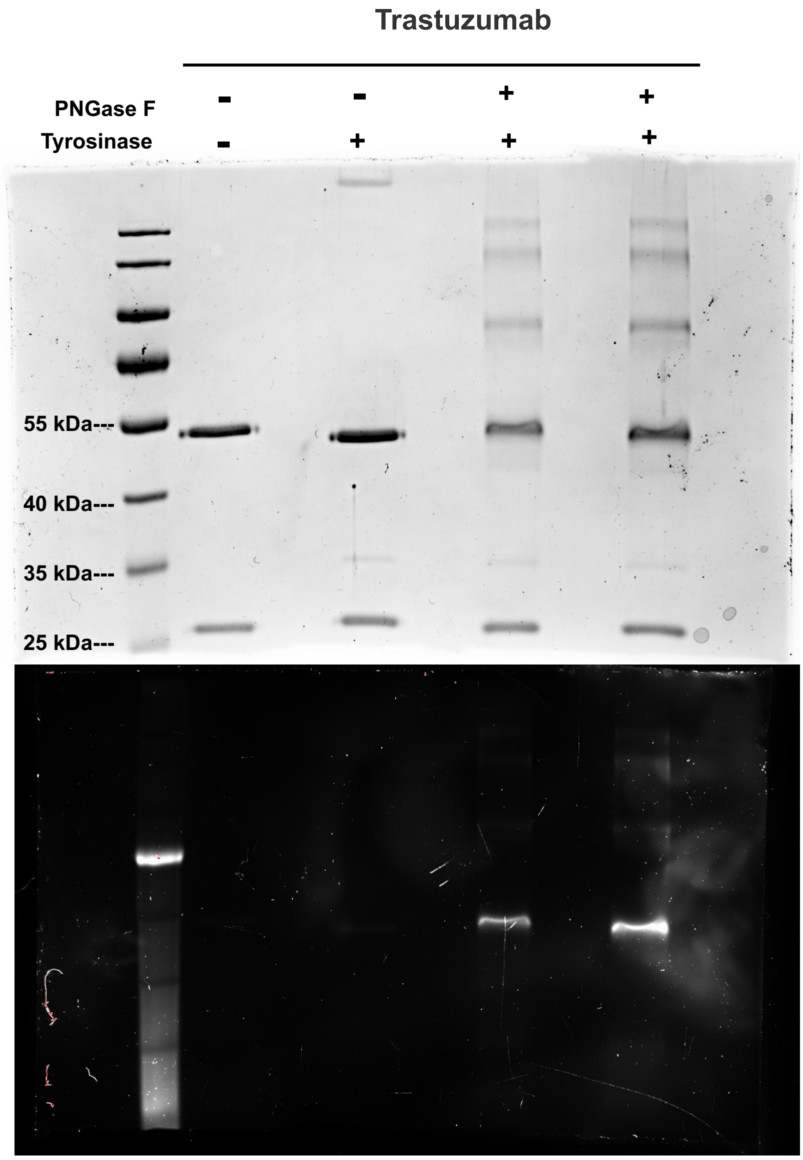

Figure S39. Raw gel figure of Figure 4B. SDS-PAGE analysis of labeled Tras-TAMRA by click reaction. Above, Coomassie stain; below, fluorescent image, Ex, 365 nm.

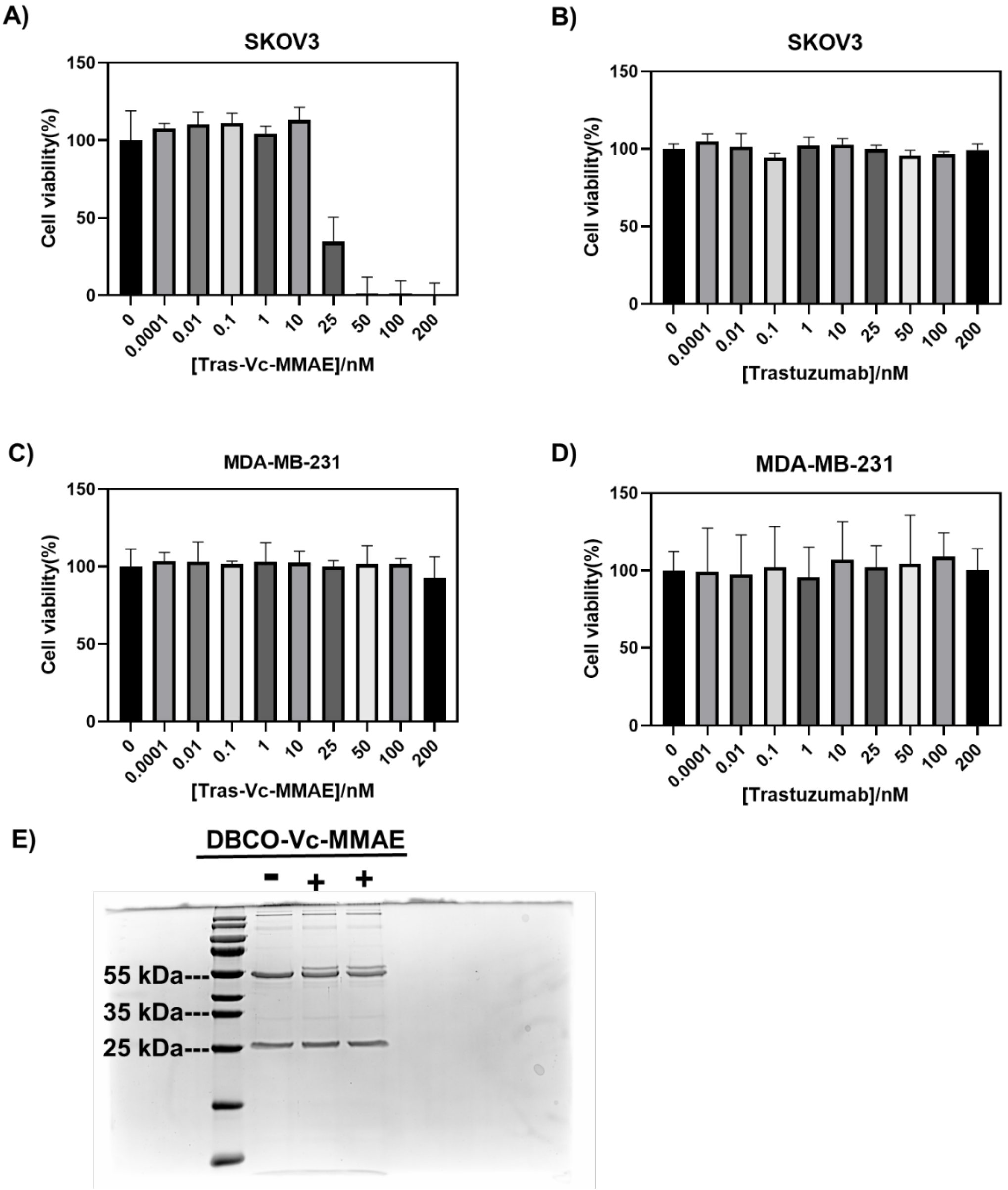

Figure S40. Concentration-dependent cellular cytotoxicity of the generated ADC and antibody. **A)** Cellular cytotoxicity of Tras-Vc-MMAE to SKOV3 cell (HER2+). **B)** Unmodified Trastuzumab showed no toxicity to to SKOV3 cell (HER2+). **C)** Tras-Vc-MMAE showed no toxicity to MDA-MB-231 cells (HER2-). **D)** Unmodified Trastuzumab showed no toxicity to MDA-MB-231 cells (HER2-). Note here [Tras-Vc-MMAE] represents the concentration of the total Tras in the mixture of antibodies, and Tras-Vc-MMAE is only a fraction of the total concentration. **E)** Raw SDS-PSGE figure for conjugation of Tras-N_3 with DBCO-VC-MMAE.

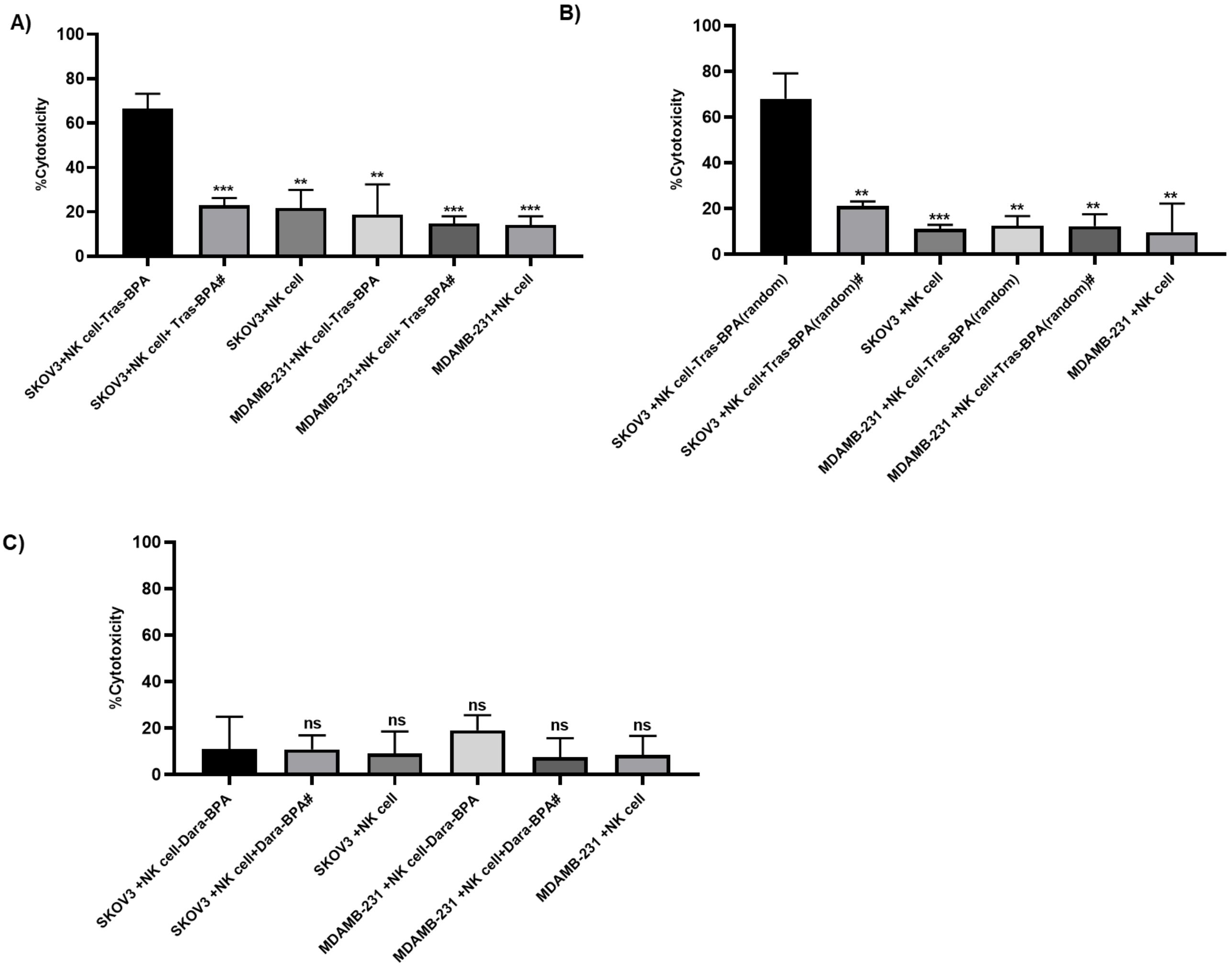

Figure S41. Cytotoxicity of antibodies with different modification methods**. A)** Cytotoxicity of the site-selectively labeled antibody Tras-BPA. **B)** Cytotoxicity of the randomly labeled antibody Tras-BPA(random). **C)** A control antibody Daratuzumab (Dara) showed no toxicity to HER2+ cells. Label # indicates groups that did not receive light irradiation. Data are presented as the mean ± s.d. of n = 3 independent experiments. *, P <0.05. **, P <0.01. ***, P < 0.001.****, P <0.0001.

The following figures (**Figures S42-S48**) show the original MS and NMR spectra of compounds synthesized in this work.

A)

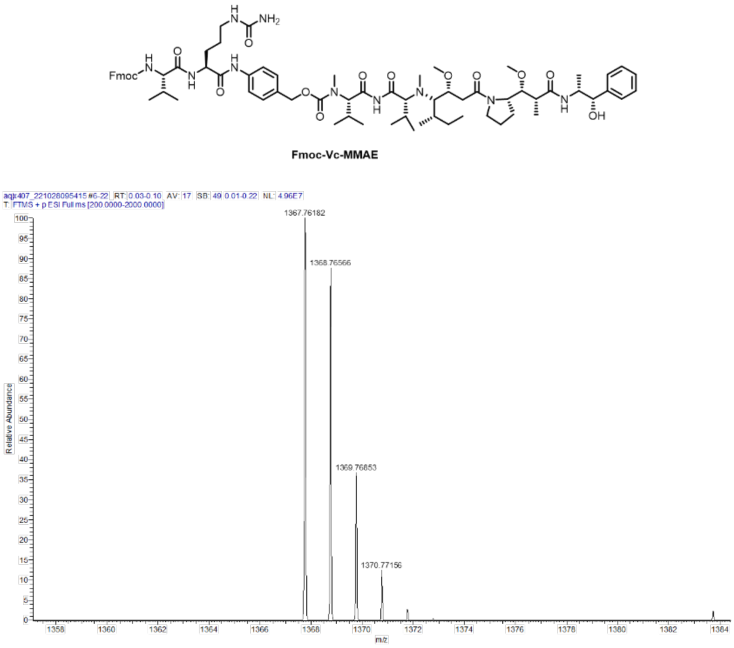

B)

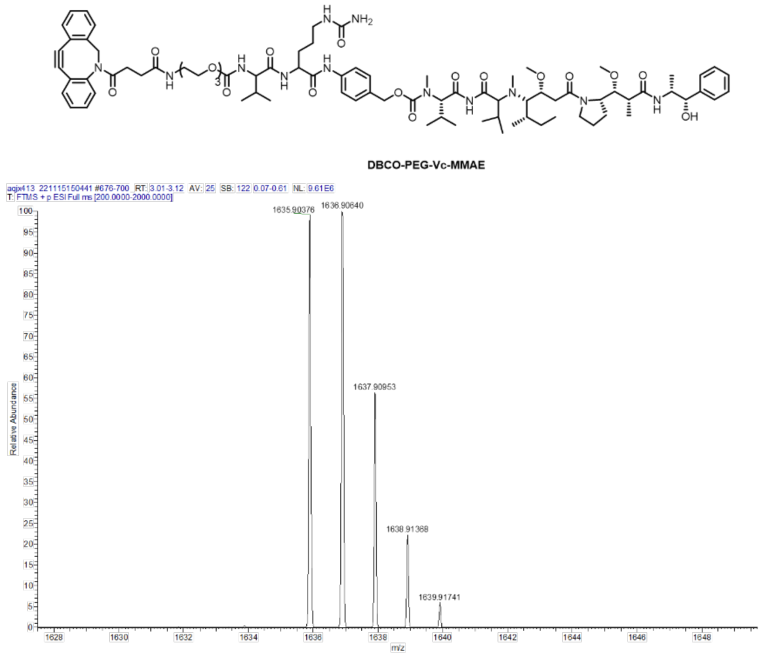

Figure S42. HR-ESI analysis of MMAE compounds. **A)** HR-ESI calculated for Fmoc-Vc-MMAE $C_{73}H_{104}N_{10}NaO_{14}^{+}$ [M+Na]$^{+}$ 1367.7626, found 1367.7618. **B)** HR-ESI calculated for DBCO-PEG-Vc-MMAE $C_{80}H_{112}N_{12}NaO_{16}^{+}$ [M+Na]$^{+}$ 1635.9049, found 1635.9064.

A)

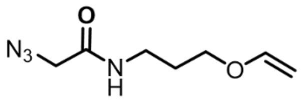

VE-azide

B)

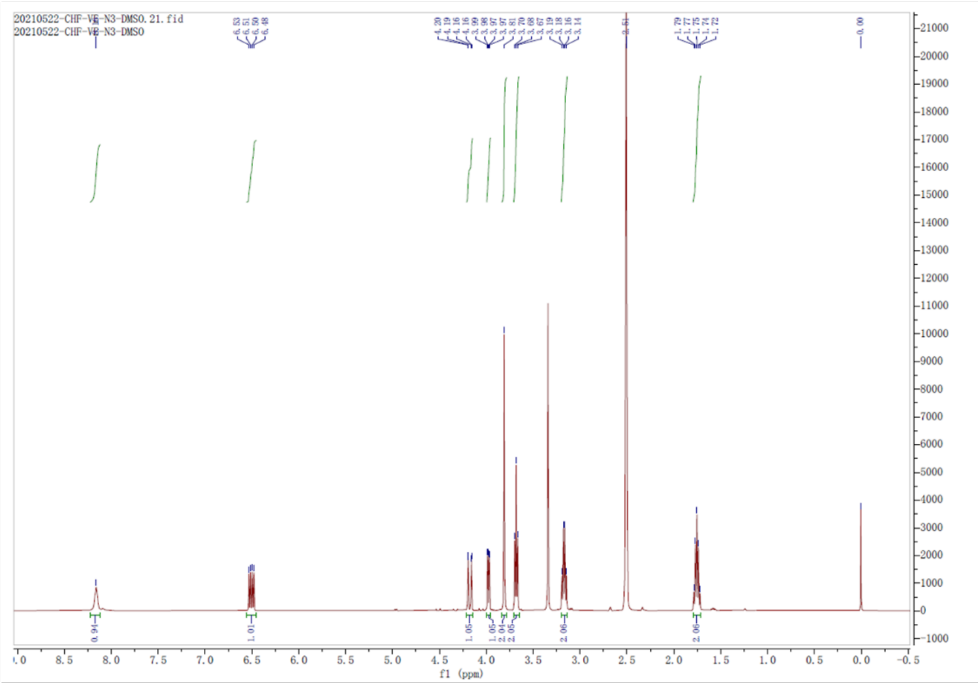

C)

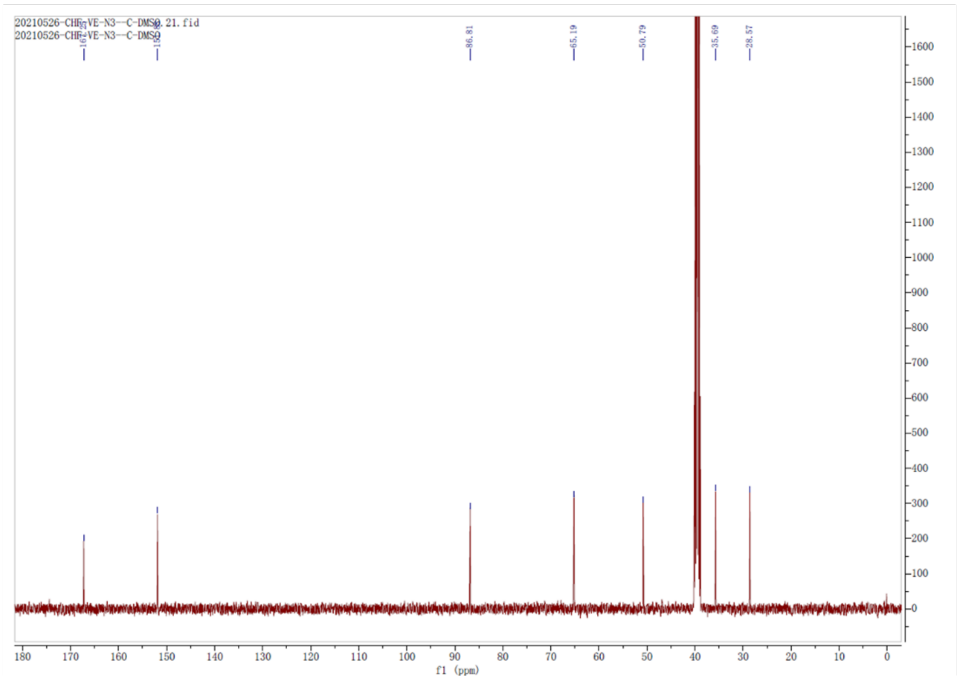

Figure S43. NMR spectra of VE-N_3. **A)** Structure of VE-N_3. **B)** H^1-NMR (400 MHz, DMSO-d_6) spectrum of VE-N_3. **C)** C^{13}-NMR (100 MHz, DMSO-d_6) spectrum of VE-N_3.

A)

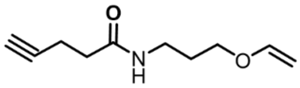

VE-alkyne

B)

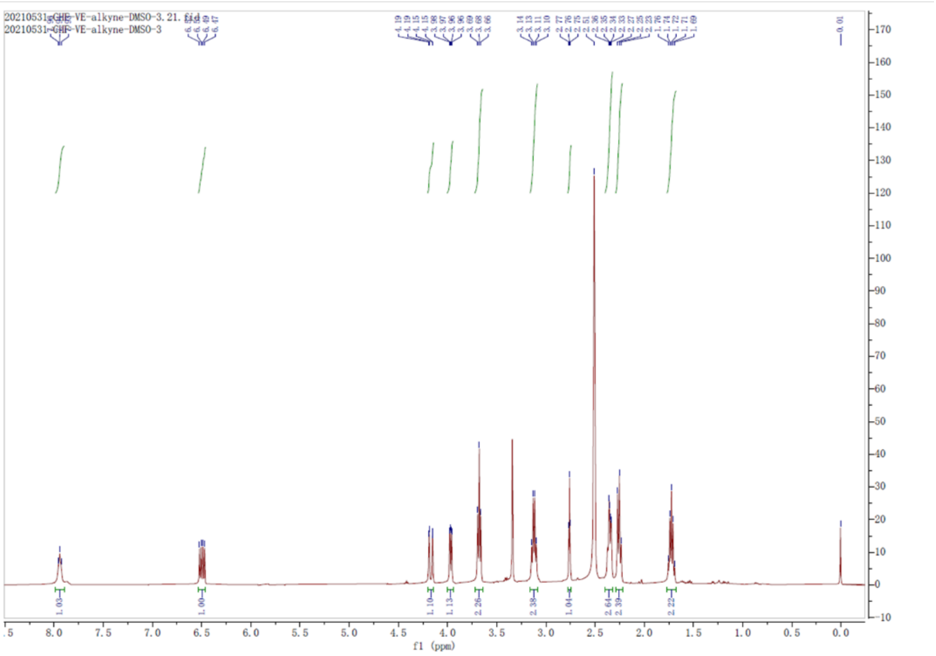

C)

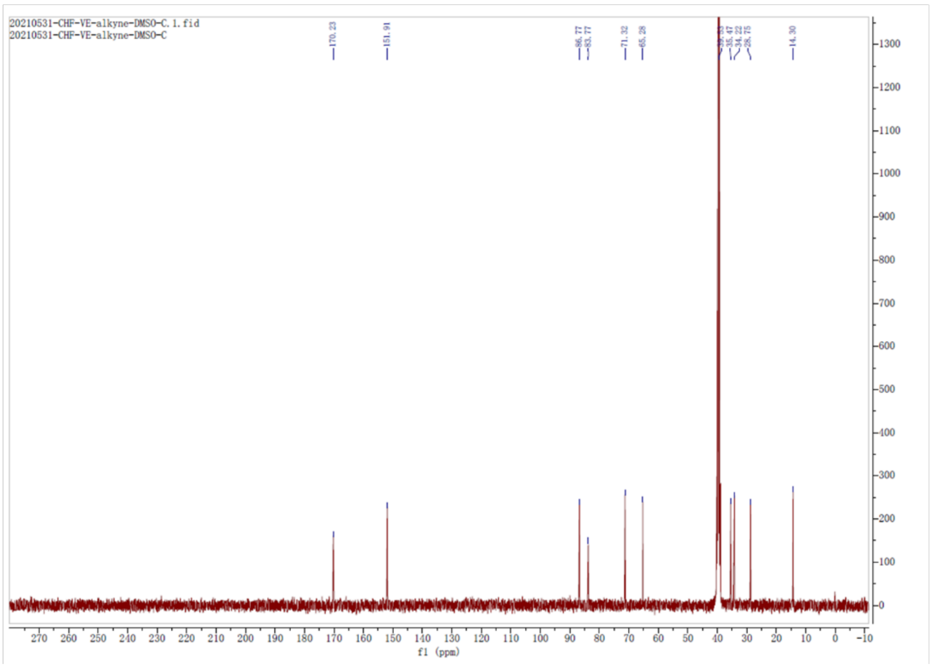

Figure S44. NMR spectrums of VE-alkyne. **A)** Structure of VE-alkyne. **B)** ^{1}H-NMR (400 MHz, DMSO-d_6) spectrum of VE-alkyne. **C)** ^{13}C-NMR (100 MHz, DMSO-d_6) spectrum of VE-alkyne.

A)

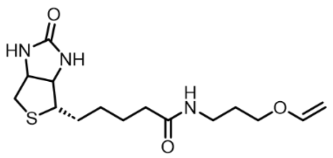

VE-Biotin

B)

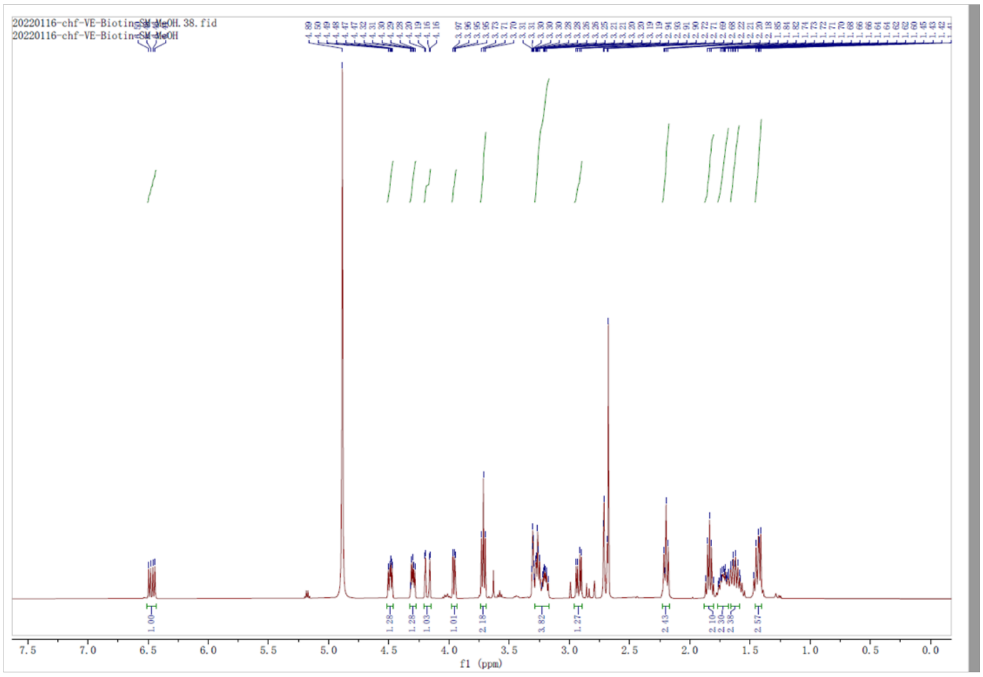

C)

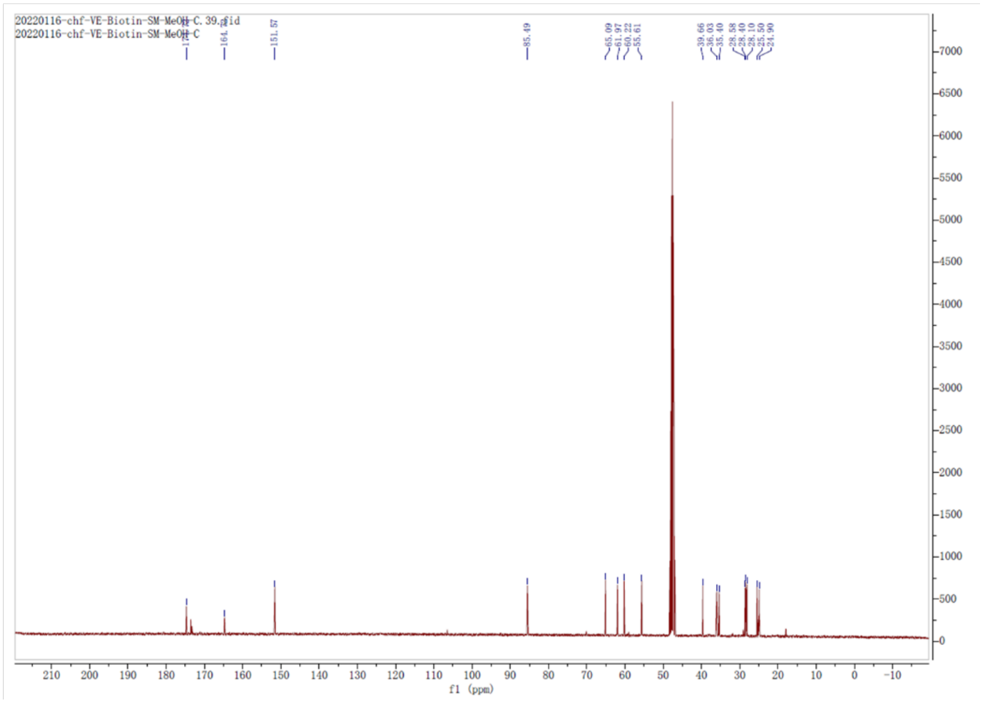

Figure S45. NMR spectrums of VE-Biotin. **A)** Structure of VE-Biotin. **B)** H^1-NMR (400 MHz, Methanol-d_4) spectrum of VE-Biotin. **C)** C^{13}-NMR (100 MHz, Methanol-d_4) spectrum of VE-Biotin.

A)

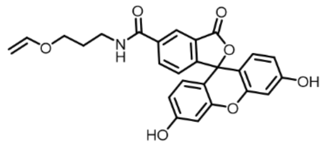

VE-FAM

B)

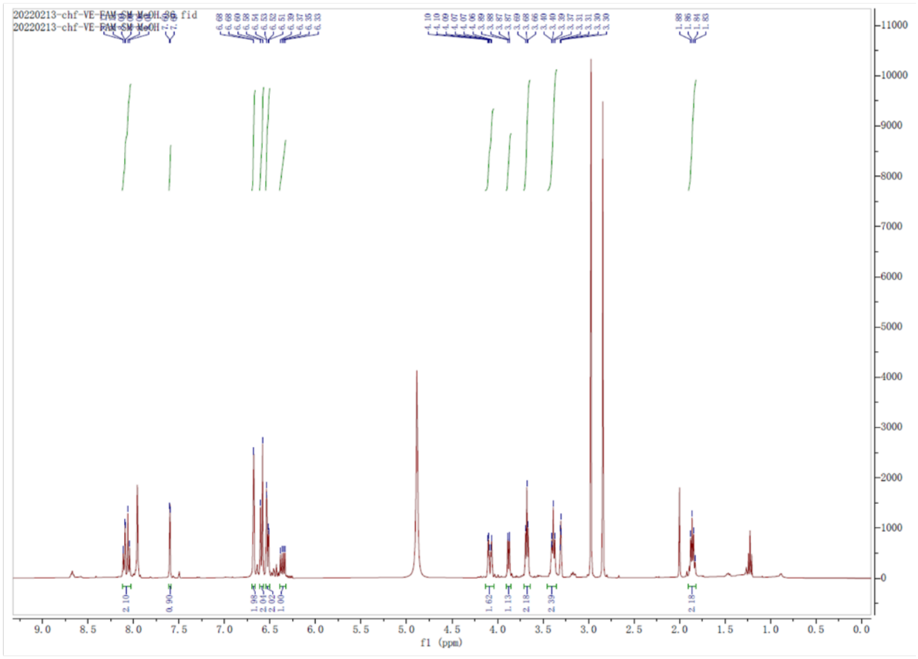

C)

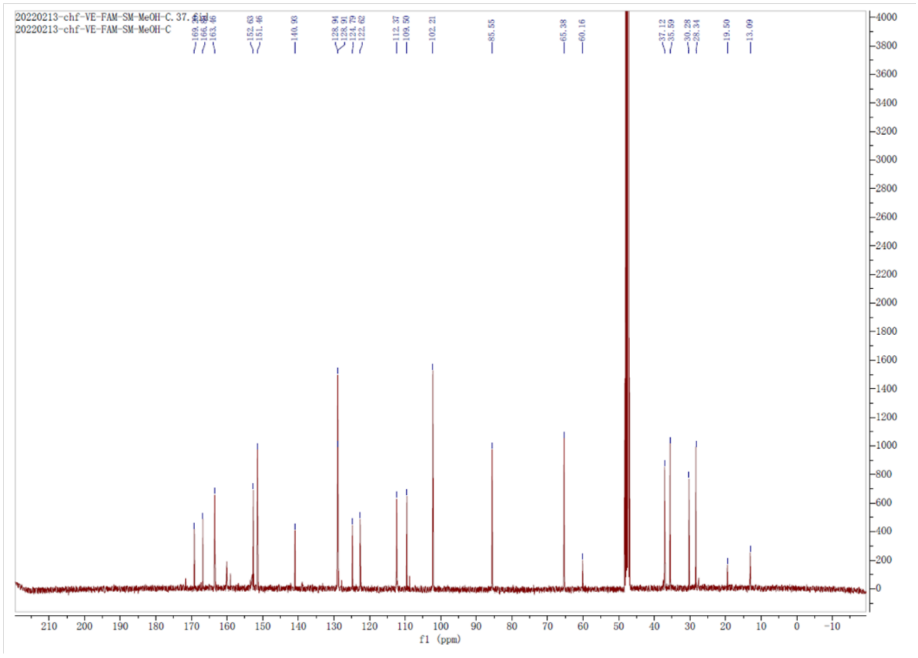

Figure S46. NMR spectrums of VE-FAM. **A)** Structure of VE-FAM. **B)** H^{1}-NMR (400 MHz, Methanol-d_4) spectrum of VE-FAM. **C)** C^{13}-NMR (100 MHz, Methanol-d_4) spectrum of VE-FAM.

A)

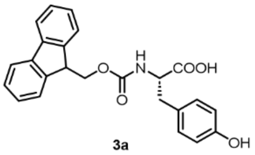

B)

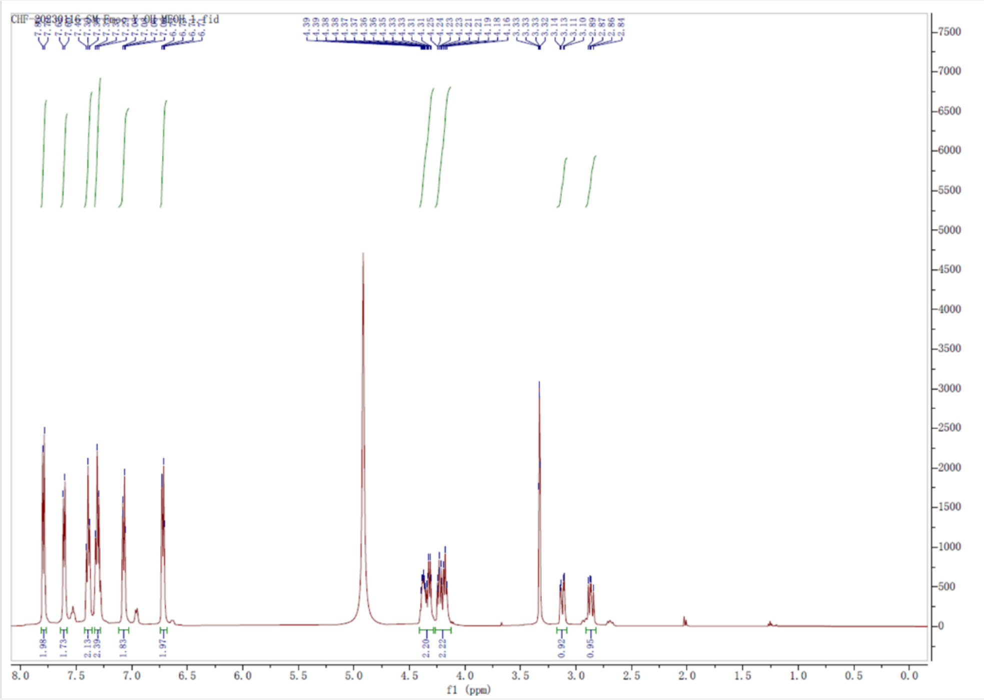

C)

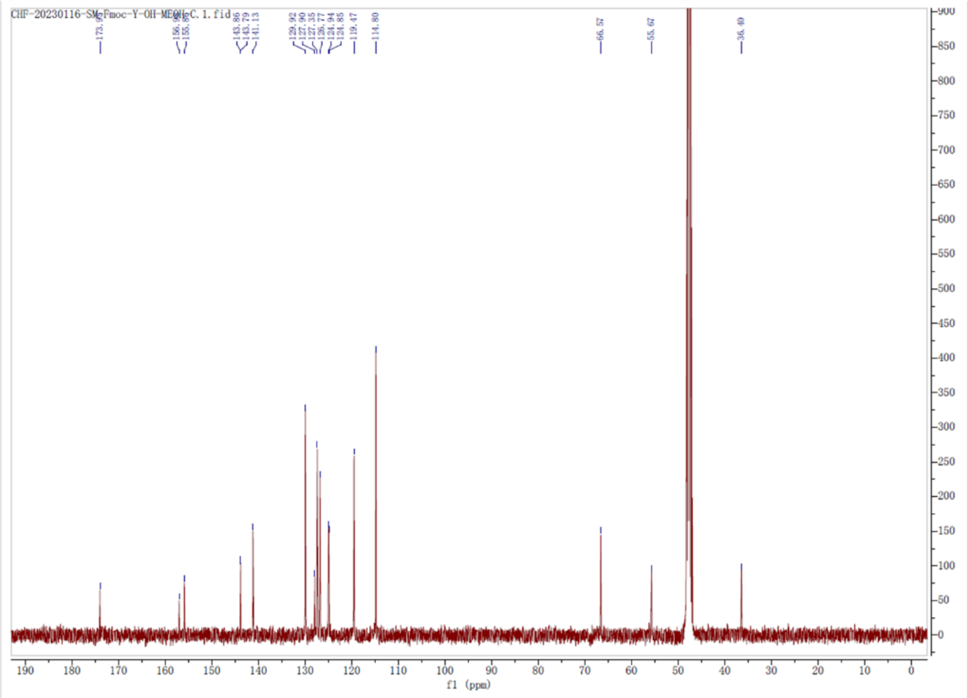

Figure S47. NMR spectrums of Fmoc-Tyr-OH 3a. **A)** Structure of 3a. **B)** H[1]-NMR (500 MHz, Methanol-*d*4) spectrum of 3a. **C)** C[13]-NMR (125 MHz, Methanol-*d*4) spectrum of 3a.

A)

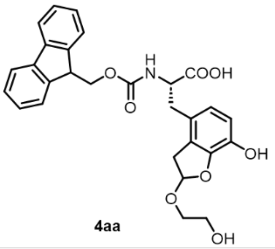

4aa

B)

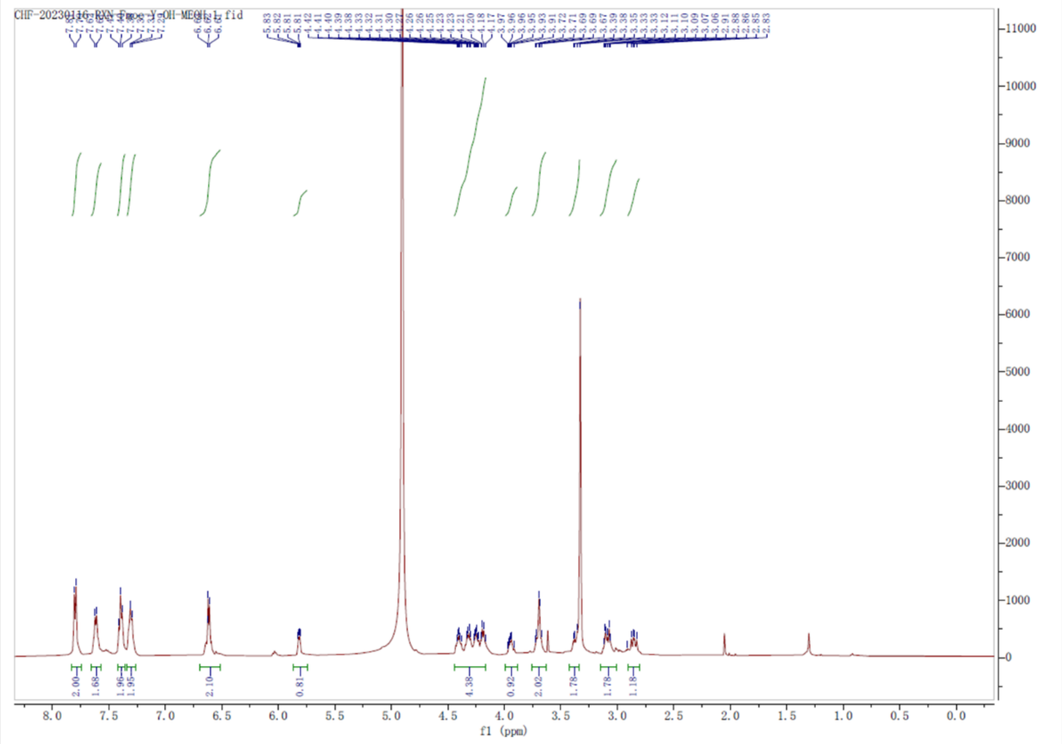

C)

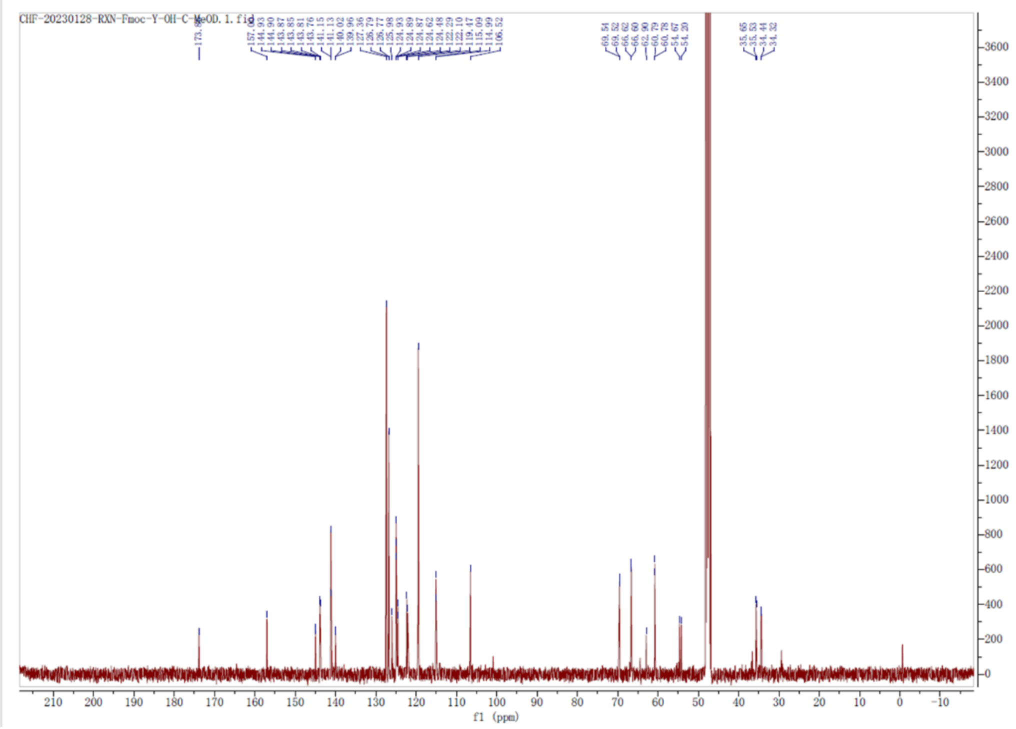

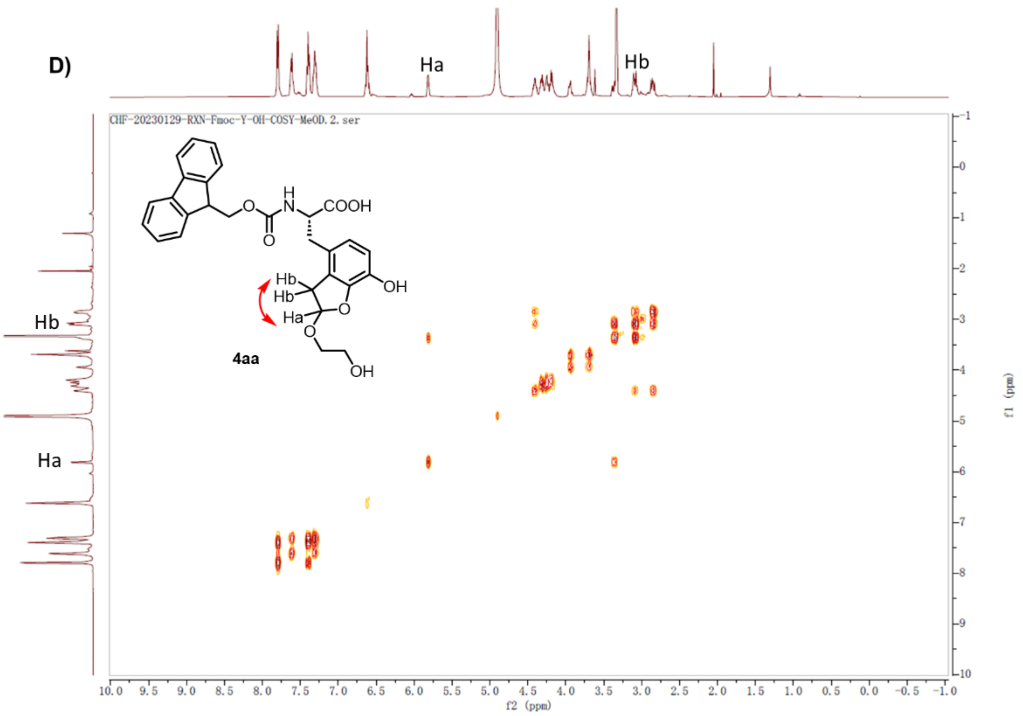

Figure S48. Structure confirmation of product 4aa. **A)** peptide reaction structure 4aa. **B)** H[1]-NMR spectrum of product 4aa. **C)** C[13]-NMR spectrum of product 4aa. **D)** H[1]-H[1] COSY spectrum of product 4aa.

References

1. Frisch, M. J.; Trucks, G. W.; Schlegel, H. B.; Scuseria, G. E.; Robb, M. A.; Cheeseman, J. R.; Scalmani, G.; Barone, V.; Petersson, G. A.; Nakatsuji, H.; Li, X.; Caricato, M.; Marenich, A. V.; Bloino, J.; Janesko, B. G.; Gomperts, R.; Mennucci, B.; Hratchian, H. P.; Ortiz, J. V.; Izmaylov, A. F.; Sonnenberg, J. L. W.; Ding, F.; Lipparini, F.; Egidi, F.; Goings, J.; Peng, B.; Petrone, A.; Henderson, T.; Ranasinghe, D.; Zakrzewski, V. G.; Gao, J.; Rega, N.; Zheng, G.; Liang, W.; Hada, M.; Ehara, M.; Toyota, K.; Fukuda, R.; Hasegawa, J.; Ishida, M.; Nakajima, T.; Honda, Y.; Kitao, O.; Nakai, H.; Vreven, T.; Throssell, K.; Montgomery Jr., J. A.; Peralta, J. E.; Ogliaro, F.; Bearpark, M. J.; Heyd, J. J.; Brothers, E. N.; Kudin, K. N.; Staroverov, V. N.; Keith, T. A.; Kobayashi, R.; Normand, J.; Raghavachari, K.; Rendell, A. P.; Burant, J. C.; Iyengar, S. S.; Tomasi, J.; Cossi, M.; Millam, J. M.; Klene, M.;Adamo, C.; Cammi, R.; Ochterski, J. W.; Martin, R. L.; Morokuma, K.; Farkas, O.; Foresman, J. B.; Fox, D. J. Gaussian 16, C.02.; Gaussian, Inc.: Wallingford, CT, **2016**.
2. Chai, J-D.; Head-Gordon, M. Long-range corrected hybrid density functionals with damped atom-atom dispersion corrections. *Phys. Chem. Chem. Phys.* **2008,** 10, 6615–6620.
3. Tomasi, J.; Mennucci, B.; Cammi, R. Quantum mechanical continuum solvation models. *Chem. Rev.* **2005,** 105, 8, 2999–3093.
4. Fukui, K. The path of chemical reactions - the IRC approach. *Acc. Chem. Res.* **1981,** 14, 12, 363–368.
5. Page, M.; McIver, J. W. On evaluating the reaction path Hamiltonian. *J. Chem. Phys.* **1988,** 88, 2, 922–935.

6. Zhao, Y.; Truhlar, D. G. The M06 suite of density functionals for main group thermochemistry, thermochemical kinetics, noncovalent interactions, excited states, and transition elements: Two new functionals and systematic testing of four M06-class functionals and 12 other functionals. *Theor. Chem. Acc.* **2008,** 120, 215–241.
7. Marenich, A. V.; Cramer, C. J.; Truhlar, D. G. Universal solvation model based on solute electron density and on a continuum model of the solvent defined by the bulk dielectric constant and atomic surface tensions. *J. Phys. Chem. B.* **2009,** 113, 6378–6396.
8. Marjoke F. Debets, S. S. v. B., Jan Dommerholt, A. (Ton) J. Dirks, Floris P. J. T. Rutjes, and Floris L. van Delft, Bioconjugation with Strained Alkenes and Alkynes. *Acc. Chem. Res.* **2011**, 44, 805-815.
9. Kong, H.; Huang, L.; Cheng, B.; Qin, K.; Zheng, M.; Yan, Z.; Zhang, Y., Visible Light-Initiated Bioorthogonal Photoclick Cycloaddition. *J. Am. Chem. Soc.* **2018**, 140 (44), 14542-14546.
10. Marmelstein, A. M.; Lobba, M. J.; Mogilevsky, C. S.; Maza, J. C.; Brauer, D. D.; Francis, M. B., Tyrosinase-Mediated Oxidative Coupling of Tyrosine Tags on Peptides and Proteins. *J. Am. Chem. Soc.* **2020**, 142 (11), 5078-5086.
